# Nanoarchitecturing of Bimetallic Metal‒Organic Frameworks for Emerging Applications in Quartz Crystal Microbalance Gas Sensors

**DOI:** 10.1002/smtd.202401808

**Published:** 2025-05-12

**Authors:** Silvia Chowdhury, Asep Sugih Nugraha, Brian Yuliarto, Yusuke Yamauchi, Yusuf Valentino Kaneti

**Affiliations:** ^1^ Australian Institute for Bioengineering and Nanotechnology (AIBN) The University of Queensland Brisbane QLD 4072 Australia; ^2^ Advanced Functional Materials Laboratory Faculty of Industrial Technology Institut Teknologi Bandung Bandung 40132 Indonesia; ^3^ Research Center for Nanosciences and Nanotechnology Institut Teknologi Bandung Bandung 40132 Indonesia; ^4^ Department of Materials Process Engineering Graduate School of Engineering Nagoya University Nagoya 464‐8603 Japan

**Keywords:** bimetallic MOFs, gas sensing, metal–organic frameworks, quartz crystal microbalance (QCM), sensors

## Abstract

Metal‒organic frameworks (MOFs) are promising materials for advanced sensors because of their large surface area, high porosity, and compositional and structural versatility. The incorporation of a secondary metal center to form bimetallic MOFs can significantly enhance sensor performance by increasing the number of adsorption sites for gas molecules, enhancing charge transfer, and improving structural stability. Additionally, the tunable structure, composition, and porosity of bimetallic MOFs allow for the design of highly selective sensors tailored to specific gases. However, their low conductivity and thermal stability limit their application in traditional chemiresistive sensors. Instead, bimetallic MOFs are well suited for mass‐sensitive gas sensors, such as quartz crystal microbalance (QCM) gas sensors, which operate at room temperature and rely on physical or chemical interactions. This review highlights recent advances in the exterior and interior nanoarchitectural control of bimetallic MOFs and their emerging applications in QCM sensors for various gas detection methods, along with the underlying sensing mechanisms. This study concludes with an overview of the challenges and future research directions in the synthesis and application of these materials for QCM gas sensors.

## Graphical abstract

This review summarizes recent progress in the design and nanoarchitectural control of bimetallic metal–organic frameworks (MOFs) and their applications in quartz crystal microbalance (QCM) sensors for the detection of various gases. It also discusses the underlying sensing mechanisms of bimetallic MOFs and concludes with challenges and future directions for advancing the synthesis and practical use of bimetallic MOFs in QCM‐based sensing technologies.

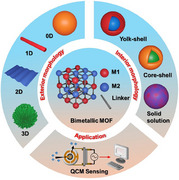

## 1 Introduction

**Figure 1:**
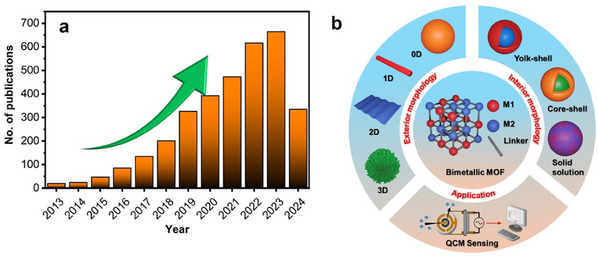
a) Trend in publications on bimetallic MOFs over the past decade according to Web of Science (October 2024). The results were obtained by searching the keyword 'Bimetallic MOF' in the Web of Science database, with the publication period set from 2013 to 2024. b) A schematic diagram summarizing the main topics covered in this review.

MOFs, also known as porous coordination polymers (PCPs), are highly crystalline porous materials formed by the coordination bonding between metal ions or clusters and organic ligands, leading to periodic multidimensional network structures.^[^ ^1^, ^2^ ^]^ These materials are characterized by their inherent porosity, large internal surface area (up to 10000 m^2^ g^−1^), tunable structure, and versatile composition, making them promising alternatives to traditional porous materials, such as zeolites and activated carbons.^[^ ^3^, ^4^, ^5^, ^6^, ^7^ ^]^ These attractive properties have enabled the application of MOFs in catalysis, adsorption, energy, sensing, gas storage and separation and drug delivery.^[^ ^8^, ^9^, ^10^, ^11^, ^12^, ^13^ ^]^ To increase the functional performance of MOFs in these applications, incorporating secondary metal ions or clusters into the frameworks of MOFs to generate bimetallic MOFs is desirable. Owing to their tunable compositions and structures and the synergistic advantages arising from having two metal centers, bimetallic MOFs have demonstrated enhanced properties compared with their monometallic counterparts, showing superior performance in various applications.^[^ ^10^, ^13^, ^14^, ^15^ ^]^ Consequently, the number of publications on bimetallic MOFs has increased exponentially in the past decade, as depicted in **Figure** **1**a.

One of the primary concerns for the welfare of human beings is air pollution, which causes 9–12 million deaths annually.^[^ ^16^ ^]^ Indoor air pollution has caused the deaths of 2.8 million individuals, whereas outdoor air pollution has been estimated to cause 4.2 million premature deaths globally.^[^ ^16^, ^17^ ^]^ Particulate matter (PM), toxic gases (e.g., nitric oxide (NO), nitrous oxide (NO_2_), ammonia (NH_3_), methane (CH_4_), sulphur dioxide (SO_2_), carbon monoxide (CO), ozone (O_3_), and hydrogen sulphide (H_2_S)), volatile organic compounds (VOCs), and radioactive and chemical warfare agents pose significant threats to human health.^[^ ^17^, ^18^, ^19^, ^20^, ^21^, ^22^ ^]^ Toxic gases are frequently emitted by factories, power plants, and residences. For example, gases, such as SO_2_, NO_2_, O_3_, and H_2_S, are highly harmful air pollutants. Long‐term exposure to these gases can lead to chronic diseases and permanent lung damage, making air quality monitoring and pollution control essential for public health.^[^ ^23^, ^24^, ^25^, ^26^ ^]^ VOCs are often defined as organic compounds with high vapor pressures and low boiling points.^[^ ^27^ ^]^ As such, they readily evaporate and produce vapors at room temperature. VOCs mainly originate from two main sources: natural sources (e.g., vegetation, wildfires, and microbial activity) and anthropogenic (human‐made) sources (e.g., household products, industrial activities, vehicle emissions, and agricultural activities). Common examples of VOCs include alcohols, aldehydes, aromatic hydrocarbons, aliphatics, ethers, and ketones.^[^ ^27^ ^]^ These VOCs typically contain diverse functional groups, such as oxygen, nitrogen, sulphur, phosphorus, or halogens (e.g., chlorine, fluorine, bromine, and iodine). Short‐term exposure to VOCs can cause irritation to the eyes, nose, and throat, along with dizziness, nausea, vomiting, and difficulty breathing.^[^ ^28^ ^]^ However, long‐term exposure may result in respiratory illnesses, neurological impairments, liver and kidney damage, and even cancer. According to a recent study, an estimated 36.4% to 39.7% of the global population has been exposed to harmful levels of VOCs, significantly increasing their risk of cancer.^[^ ^29^ ^]^ Therefore, it is imperative to develop highly sensitive, selective, and stable sensors for detecting hazardous gases, as these pollutants pose significant risks to both human health and the environment.

Gas sensors are devices that can detect the presence of gases in the environment. They typically operate by responding to a physical or chemical change induced by the adsorption and desorption of gas molecules.^[^ ^18^ ^]^ Gas sensors can be implemented in a variety of climatic conditions, depending on the target application. In this regard, humidity and temperature can significantly affect the sensor's response toward the gas or vapor under investigation. Common gas sensors include electrochemical gas sensors, chemiresistive gas sensors, optical gas sensors, and mass‐sensitive gas sensors.^[^ ^30^, ^31^, ^32^, ^33^, ^34^ ^]^ Electrochemical gas sensors operate by measuring the current resulting from the oxidation or reduction of the target gas at the electrode.^[^ ^35^ ^]^ Their advantages include high sensitivity, good selectivity, fast response, and low power consumption. Chemiresistive gas sensors detect gases by measuring the change in electrical resistance of a sensing material when it interacts with gas molecules.^[^ ^36^ ^]^ These sensors present several advantages, including easy fabrication, low cost, high sensitivity, fast response, and miniaturization potential. However, electrochemical and chemiresistive sensors require conductive sensing materials and good thermal and/or chemical stability. MOFs are generally insulators and may change in composition when exposed to high temperatures (200–500 °C in chemiresistive gas sensors) or harsh chemicals (e.g., acidic or basic electrolytes in electrochemical gas sensors), making them rather unsuitable for these types of sensors. Optical gas sensors operate by measuring changes in light properties (such as absorption, emission, or scattering) when they interact with a sensing material.^[^ ^37^ ^]^ Common optical gas sensors include fluorescence and luminescence gas sensors, surface plasmon resonance (SPR) gas sensors, and surface‐enhanced Raman scattering (SERS) gas sensors. These sensors exhibit high sensitivity, enabling real‐time, selective, and noninvasive gas detection. However, they require expensive instruments and trained operators. Furthermore, the lack of distinct optical properties (e.g., lack of fluorescence, luminescence, and Raman scattering properties) in many MOFs renders them ineffective for optical sensors.

In contrast, MOFs represent promising materials for mass‐sensitive gas sensors, as they operate at room temperature and do not require heating.^[^ ^38^ ^]^ Furthermore, these sensors function by detecting mass arising from interactions between the target gas and the sensing material.^[^ ^39^ ^]^ The presence of diverse functional groups within MOFs enhances their ability to promote physical or chemical interactions with the target, making them highly effective for direct use in mass‐sensitive gas sensors. There are numerous classifications of mass‐sensitive gas sensors, with resonant microcantilever and QCM gas sensors being the most prevalent.^[^ ^18^, ^40^, ^41^ ^]^ Among mass‐sensitive gas sensors, QCM sensors are particularly attractive because of their room‐temperature operation, low cost, simple fabrication, and easy miniaturization. These sensors work by detecting frequency shifts caused by mass changes in the sensing material as a result of gas adsorption or desorption. As the target gas adsorbs, the sensor frequency decreases because of the added mass.^[^ ^10^, ^42^ ^]^ When the gas desorbs, the frequency gradually returns to its initial value.

In the past, QCM sensors for monitoring hazardous gases have relied on organic polymers, carbon, and metal oxides.^[^ ^43^, ^44^ ^]^ Recently, MOFs have gained increasing attention as sensing materials for hazardous gas monitoring via QCM technology because of their large surface area, rich functional groups, versatile structure and composition, and inherent porosity. The extensive surface area and inherent porosity of MOFs provide rich adsorption sites for gas molecules and increase accessibility to these sites. Furthermore, the controllable composition, morphology, and porosity of MOFs enable precise tuning of their properties to optimize gas detection. For example, gas molecules differ in size, and the use of MOFs with appropriately sized pores can enhance the diffusion of these molecules in and out of the framework, thereby improving the sensing performance. Additionally, the diverse functional groups in MOFs can promote strong interactions with the target gas through hydrogen bonding, π–π interactions or van der Waals forces.^[^ ^30^ ^]^ To date, most studies on MOF‐based QCM gas sensors have focused primarily on pure MOFs and MOF‐derived carbons.^[^ ^42^, ^44^, ^45^ ^]^ They have been explored as sensing layers for QCM gas sensors, particularly for detecting various toxic gases and VOCs.^[^ ^46^, ^47^, ^48^, ^49^, ^50^ ^]^ In contrast, research on the development of bimetallic MOF‐based QCM gas sensors for hazardous gas monitoring has been limited but has increased in recent years. The introduction of secondary metal nodes in bimetallic MOFs provides significant advantages for gas‐sensing applications, including enhanced charge transfer and stronger interactions with target gases.^[^ ^51^, ^52^, ^53^ ^]^ Moreover, the selection of the secondary metal and the metal ratio can be tailored to enhance selectivity, as different metals exhibit varying interactions with gas molecules.

In the past, several reviews have covered the applications of MOF‐based materials for chemiresistive,^[^ ^36^, ^54^, ^55^ ^]^ capacitive,^[^ ^56^ ^]^ luminescence,^[^ ^57^, ^58^ ^]^ fluorescence,^[^ ^59^, ^60^ ^]^ and optical gas sensors.^[^ ^37^, ^61^ ^]^ In comparison, relatively few reviews have focused on MOF‐based QCM gas sensors, with existing reviews concentrating on MOF‐derived carbons and various types of monometallic MOFs.^[^ ^30^, ^45^ ^]^ Notably, limited attention has been given to the use of bimetallic MOFs in QCM gas sensors, despite their potential to offer enhanced structural stability, tunable electronic properties, and superior adsorption capabilities. Furthermore, these reviews often provide only a limited discussion on the sensing mechanisms of MOF‐based QCM gas sensors in detecting various gases.

Unlike previous reviews, this paper provides a comprehensive analysis of strategies for achieving both exterior and interior nanoarchitectural control in bimetallic MOFs. Exterior nanoarchitecturing involves the dimensional tuning of bimetallic MOFs into 0D, 1D, 2D, and 3D structures (Figure 1b), whereas interior nanoarchitecturing focuses on tuning the internal structures into hollow, core‒shell, and yolk‒shell configurations (Figure 1b). Furthermore, this review highlights recent advancements and the expanding applications of bimetallic MOFs in QCM sensors for hazardous gas detection. This review also provides a more detailed and focused discussion on sensing mechanisms than previous reviews do, providing a detailed analysis of how different metals influence the sensitivity and selectivity of bimetallic MOFs, which is supported by both experimental and theoretical studies. Finally, the review discusses current challenges and outlines future research directions for advancing the use of bimetallic MOFs in the practical monitoring of hazardous gases. This review is expected to provide valuable insights into the design and optimization of bimetallic MOFs for the development of highly sensitive and selective MOF‐based QCM sensors.

## 2 Metal‒Organic Frameworks (MOFs)

### 2.1 Definition and Properties of MOFs

**Figure 2:**
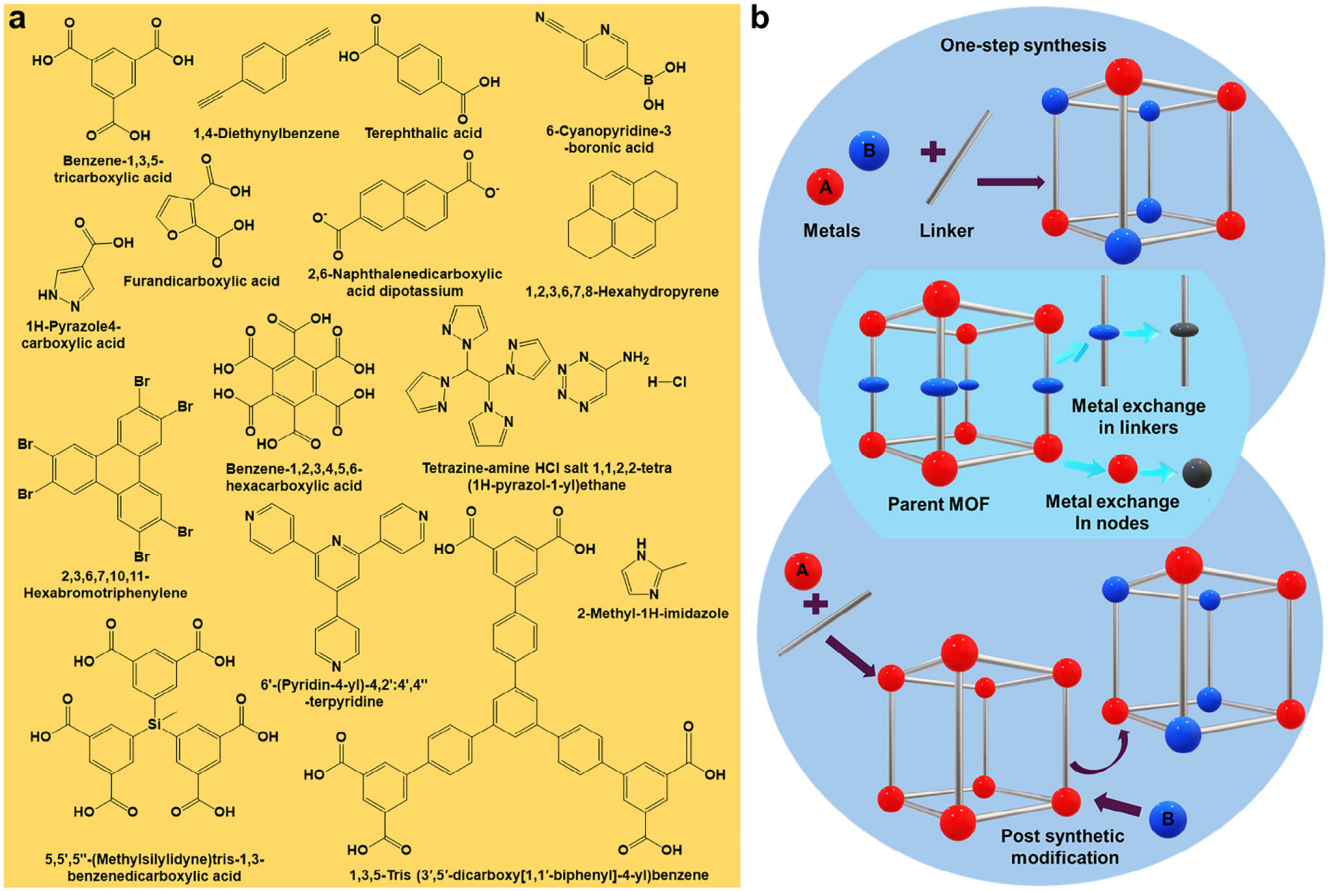
a) Examples of coordination geometries for organic ligands commonly used in MOF synthesis. b) Schematic representation of bimetallic MOF fabrication through one‐pot synthesis and PSME. Metal exchange can occur either in the metal‐containing ligands or by partial or complete metal replacement at the nodes.

MOFs are crystalline porous materials formed by linking organic ligands (such as carboxylate ligands and other negatively charged ligands) with metal components (such as metal ions or clusters).^[^ ^62^, ^63^ ^]^ Their composition and structure are highly tunable, as they can be prepared by combining different combinations of metal ions or clusters and organic ligands. To date, more than 90 000 MOFs have been experimentally synthesized, and more than 500000 different MOF structures have been theoretically simulated.^[^ ^64^, ^65^ ^]^ The metal nodes in MOFs are highly diverse and can include rare earth metals (e.g., La^3+^, Eu^3+^, Tb^3+^, Ce^4+^), alkali metals (e.g., Na^+^, K^+^, Li^+^), transition metals (e.g., Zn^2+^, Fe^3+^, Cr^3+^, and Cu^2+^) or alkaline earth metals (e.g., Mg^2+^, Ca^2+^, Ba^2+^).^[^ ^66^, ^67^, ^68^ ^]^ These metal ions provide a versatile coordination environment that can accommodate various geometries, including trigonal, tetrahedral, bipyramidal square, and pyramidal octahedral geometries. The reversible formation of coordination bonds between metal ions and organic ligands is facilitated by the inherent ability of metal‒ligand bonds, allowing MOFs to possess well‐organized framework structures.^[^ ^69^, ^70^ ^]^ Additional factors to consider include the coordination geometry, oxidation state, and coordination number (2‐7).^[^ ^66^ ^]^ The metal coordination geometry can assume various configurations, including Y‐ or T‐shaped, square‐planar, tetrahedral, cubic, square‐pyramidal or octahedral configurations (**Figure** **2**a). The organic ligands in MOFs are also highly varied, with the most common types including imidazolates and carboxylic acid‐based ligands (including di‐, tri‐, and tetracarboxylic acid‐based ligands). The highly versatile composition of MOFs gives them advantages over traditional porous materials, such as zeolites, which are limited to aluminosilicate minerals.

Another key characteristic of MOFs is their inherent porosity, which can be tuned by combining different metal ions and organic ligands to create different pore sizes and shapes or by incorporating pore‐expanding agents or templates.^[^ ^64^, ^71^, ^72^ ^]^ MOFs are also known for their large surface areas, which originate from their large internal and void spaces within the framework. The surface area of MOFs can reach up to 10000 m^2^ g^−1^, significantly surpassing that of other microporous materials, such as zeolites (600–800 m^2^ g^−1^) and activated carbon (1000–3000 m^2^ g^−1^).^[^ ^73^, ^74^ ^]^ The high surface area and tunable porosity of MOFs render them useful for gas‐sensing applications, as they can provide many adsorption sites for gas molecules and promote selective adsorption. Although MOFs outperform traditional porous materials such as zeolites in terms of porosity, surface area, and versatility, they generally have lower chemical and thermal stability.^[^ ^75^ ^]^ Most MOFs decompose at temperatures between 300–500 °C in air, whereas zeolites are thermally stable up to 800 °C.^[^ ^76^ ^]^ Additionally, MOFs are generally less stable than zeolites in harsh environments, such as acidic or basic environments. To address these limitations, various strategies, such as multimetallic composition,^[^ ^14^, ^77^ ^]^ hybridization,^[^ ^78^, ^79^ ^]^ and phase conversion,^[^ ^80^, ^81^ ^]^ have been proposed to increase their stability.

## 3 Bimetallic MOFs

### 3.1 Definition, Properties, and Advantages of Bimetallic MOFs for Gas Sensing

Bimetallic MOFs are crystalline porous materials composed of two different metal ions or clusters coordinated to organic ligands (Figure 2b).^[^ ^82^, ^83^ ^]^ This dual‐metal composition offers several advantages for sensing applications.^[^ ^84^ ^]^ The presence of two metals in the framework of bimetallic MOFs can promote synergistic interactions due to the unique electronic, structural, and catalytic properties induced by combining two different metals.^[^ ^85^ ^]^ In terms of electronic properties, the addition of a secondary metal can modify the electronic structure of the MOF and enhance charge transfer between gas molecules and the bimetallic framework.^[^ ^86^ ^]^ Furthermore, it can lower the energy barrier for adsorption and desorption,^[^ ^51^, ^53^ ^]^ leading to increased sensitivity and improved response‐recovery properties. Additionally, the presence of a secondary metal can improve the structural integrity of the MOF, enhancing its chemical and thermal stability. The thermal and chemical stability of bimetallic MOFs strongly depends on the metal distribution and composition.^[^ ^14^, ^87^ ^]^ When two metals are homogeneously mixed or when a metal with higher thermal and chemical stability is dominant, the resulting bimetallic MOFs generally possess superior stability to their monometallic counterparts.

Bimetallic MOFs can exhibit higher specific surface areas (SSAs) than their monometallic counterparts with proper modulation or optimization of the metal composition, leading to more adsorption sites for gas molecules.^[^ ^10^, ^13^ ^]^ For example, the SSAs of Co*_x_* Mn_1‐_ *_x_*‐MOF‐74 samples (≈490–519 m^2^ g^−1^) exceeded those of Co‐MOF‐74 (477.3 m^2^ g^−1^) and Mn‐MOF‐74 (481.3 m^2^ g^−1^) samples.^[^ ^88^ ^]^ However, this is not always the case, as some studies have reported bimetallic MOFs with lower SSAs than their monometallic counterparts.^[^ ^86^, ^89^ ^]^ Therefore, careful tuning of the metal ratio is essential for achieving bimetallic MOFs with higher SSAs than their monometallic counterparts. Moreover, the appropriate selection of the secondary metal and precise tuning of the metal ratio are critical for optimizing the sensitivity and selectivity of bimetallic MOFs toward specific gases, as different metals exhibit varying binding affinities for various gases.^[^ ^90^, ^91^ ^]^ Additionally, the pore size of bimetallic MOFs can be tailored to further increase their adsorption of specific gases, increasing their selectivity.^[^ ^12^ ^]^ In summary, the enhanced electronic, structural, and catalytic properties of bimetallic MOFs render them more promising than their monometallic counterparts for practical gas‐sensing (including QCM gas sensing) applications.

### 3.2 Classification of Bimetallic MOFs

On the basis of the distribution of the metal components, bimetallic MOFs can be classified into “solid solution” MOFs and core‒shell MOFs. In “solid solution” bimetallic MOFs, the two different metal ions are uniformly distributed throughout the framework.^[^ ^14^ ^]^ This homogeneous mixing at the atomic level ensures that each metal ion can interact synergistically with the other. Additionally, the uniform distribution of metals gives rise to consistent and uniform active or adsorption sites, as well as improved structural stability and resistance to degradation. In “solid solution” bimetallic MOFs, there are two possible ways in which the metals are spatially arranged: (1) two different metals are present in the same secondary building unit (SBU), and mixed metal SBUs exist throughout the MOF structure, and (2) each SBU is made up of the same type of metal, and the two different SBUs are well mixed within the MOF structure.^[^ ^83^ ^]^ Generally, the mixing of two metals with similar ionic radii and coordination numbers is preferred to increase the likelihood of having the two metals present in the same SBU. However, determining the exact arrangement of the metal ions in bimetallic MOFs remains a challenging issue. Owing to their uniformity, “solid solution” bimetallic MOFs are especially useful for sensing applications, where consistent active (adsorption) sites and a uniform pore environment are desired.

Unlike “solid solution” bimetallic MOFs, in core‐shell (including yolk‐shell) bimetallic MOFs, one metal forms the core (yolk) of the framework, whereas the other metal forms a shell around this core (yolk). This core (yolk)‐shell arrangement clearly separates the two metals, with one being predominantly at the surface and the other being in the interior.^[^ ^14^ ^]^ In core (yolk)‐shell bimetallic MOFs, synergistic effects usually arise from the contact between the inner metal and the outer metal at the interface. Furthermore, the MOF shell can protect the MOF core from harsh environments (e.g., corrosive and oxidative conditions), leading to good long‐term stability.

### 3.3 General Methods for Fabricating Bimetallic MOFs

In terms of synthesis methods, two general methods can be used to fabricate bimetallic MOFs: (i) one‐pot synthesis and (ii) postsynthetic modification methods. Each method has its own advantages and disadvantages, which will be discussed below.

#### 3.3.1 One‐Pot (Direct) Synthesis Method

In the one‐pot synthesis method, two metal precursors are directly mixed with the organic ligand to form the bimetallic MOF (Figure 2b). This synthesis can be performed via various techniques: (i) at ambient temperature and pressure through aging/precipitation, ultrasonication, or electrochemical or mechanochemical methods; (ii) at elevated temperatures via the microwave method; or (iii) at elevated temperatures and pressures via the hydrothermal (aqueous) or solvothermal (nonaqueous) method in stainless‐steel autoclaves.^[^ ^92^, ^93^ ^]^ The aging or precipitation method is facile, rapid, scalable and energy efficient. However, it often requires long synthesis times and provides less control over the crystal size and phase purity of the bimetallic MOFs.^[^ ^94^ ^]^ The ultrasonication method employs high‐frequency sound waves to produce cavitation bubbles in the liquid reaction medium. These bubbles possess high local temperatures (≈4727 °C) and pressures (≈1000 bar), and they undergo very fast heating and cooling (>734 °C s^−1^) upon the application and removal of ultrasound, respectively.^[^ ^95^ ^]^ As a result, the nucleation and growth of bimetallic MOF crystals are rapidly accelerated. This method is rapid, scalable, and nonintensive and produces monodisperse bimetallic MOF particles. However, the resulting bimetallic MOF products tend to be very small in size, exhibit poor crystallinity, and have many structural defects due to the extreme heating and cooling rates. Hence, this method is not suitable for MOFs that require long crystallization times. The electrochemical method involves the application of an electric potential to the metal electrode (anode) to promote the dissolution of metal ions and their subsequent reaction with organic ligands.^[^ ^96^ ^]^ This method is particularly useful for fabricating bimetallic MOF thin films and growing bimetallic MOFs on conductive substrates. However, it has several limitations, as it requires conductive electrodes/substrates and expensive equipment, and it does not work well for MOFs with nonelectroactive metal centers. Additionally, this method typically offers less precise control over the crystallization process of bimetallic MOFs than do the aging or hydro/solvothermal methods, which require careful optimization of the current, voltage, and electrolyte composition to obtain a uniform morphology. The mechanochemical synthesis of bimetallic MOFs involves grinding two metal precursors and organic ligands together via ball milling, a mortar and pestle, or other mechanical forces to induce a reaction.^[^ ^97^ ^]^ This method is scalable and environmentally friendly, as it does not require the use of solvents or high temperatures. However, it offers limited control over the particle size and morphology of the resulting bimetallic MOF products.

Compared with low‐temperature methods, high‐temperature methods, such as microwave and hydro/solvothermal methods, generally offer more precise control over the particle size and morphology of bimetallic MOF products. The microwave method employs microwave radiation to rapidly heat the reaction mixture, enabling the rapid formation of bimetallic MOFs.^[^ ^98^ ^]^ Compared with the conventional hydro/solvothermal method, this method offers faster and more uniform heating, resulting in more crystalline MOF products. However, it has several drawbacks, including the need for expensive microwave reactors and challenges in scalability, as it requires specialized high‐power reactors with controlled heating zones to achieve consistent heating. The hydrothermal/solvothermal method involves heating a mixture of metal salts and organic ligands in water (hydrothermal) or organic solvents (solvothermal) within a sealed autoclave at elevated temperatures (typically 100–200 °C) and pressures (≤3 MPa or 30 bar).^[^ ^99^ ^]^ This method enables the synthesis of bimetallic MOFs with high crystallinity and purity and fewer defects because of slow and controlled growth. Additionally, adjusting the reaction temperature, time, and solvent enables more precise control over the size, shape, and porosity of the resulting bimetallic MOFs. However, this method uses high‐pressure sealed autoclaves, which can be costly and hazardous due to the risk of explosion if not handled properly.

**Figure 3:**
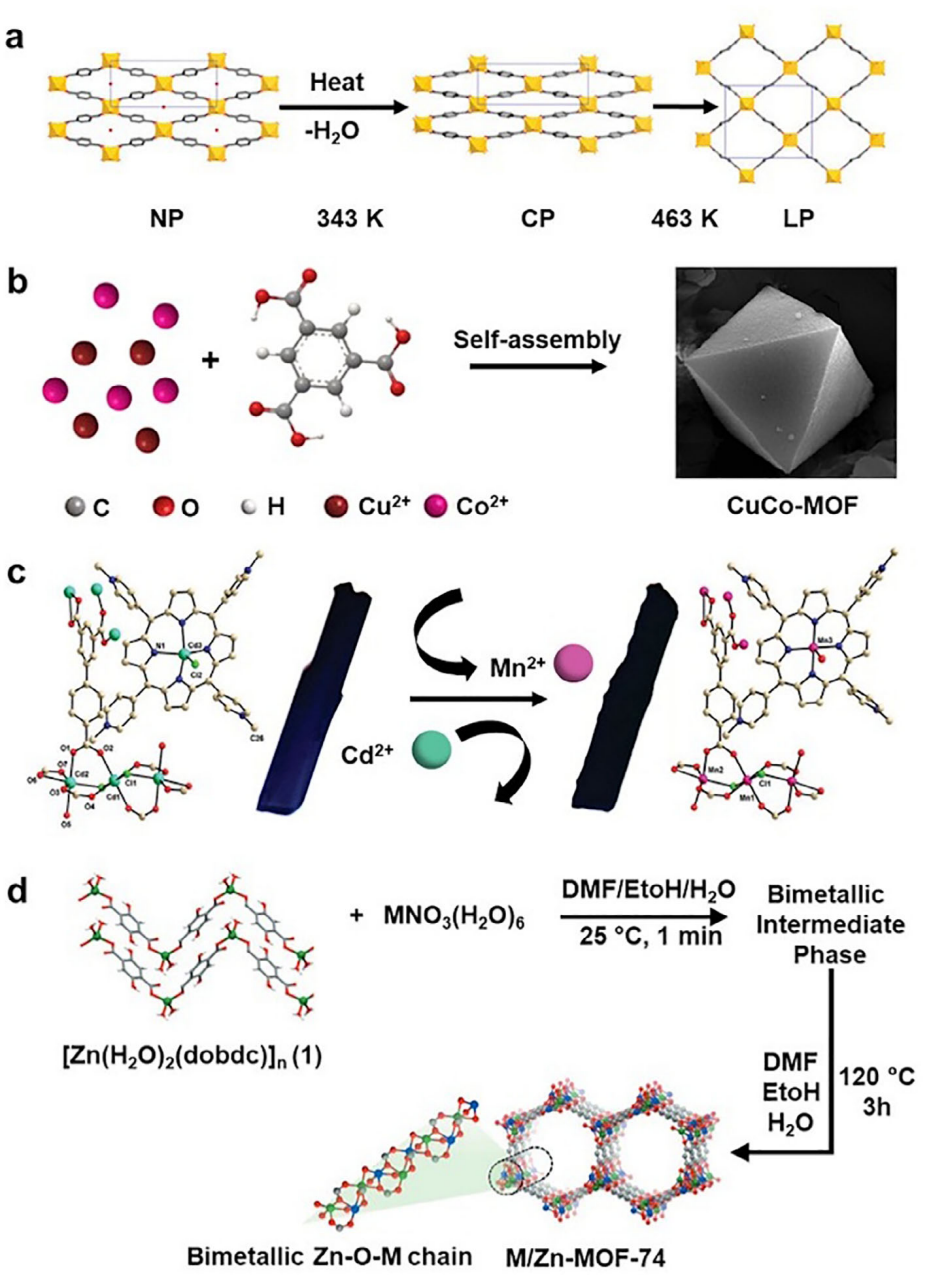
a) Schematic illustration showing the formation of two distinct phases of MIL‐53(Cr/Fe) MOFs during one‐pot synthesis. Reproduced with permission.^[^ ^100^ ^]^ Copyright 2012, Royal Society of Chemistry. b) Schematic diagram showing the one‐pot synthesis of CuCo‐MOF. Reproduced with permission.^[^ ^104^ ^]^ Copyright. 2020, Springer Nature. Template‐directed postsynthetic modification methods for creating bimetallic c) MnCd MOFs. (Reproduced with permission.^[^ ^109^ ^]^ Copyright 2012, American Chemical Society) and d) M/Zn‐MOF‐74 (Reproduced with permission.^[^ ^113^ ^]^ Copyright. 2017, Wiley‐VCH).

The one‐pot synthesis method is generally advantageous because of its rapid, low cost, and simple nature. However, it has several drawbacks, including difficulties in controlling the metal distribution and introducing defects.^[^ ^83^ ^]^ Furthermore, it is important to note that the utilization of the one‐pot synthesis method does not always guarantee a uniform distribution of the two metals in the MOF structure. This is due to various factors, such as differences in the kinetics, solubility, reactivity, ionic radius, and coordination number of the two metal ions. To successfully incorporate the second metal into the MOF framework, careful control of the synthesis conditions is essential to prevent the formation of mixed MOF phases. Moreover, factors such as solvent selection (which influences solubility), the molar ratio of the two metal precursors, the reaction time, and the pH must also be carefully optimized.^[^ ^14^ ^]^ Generally, if the second metal ions possess a relatively similar coordination geometry and ionic radius as the first metal ions do, a uniform distribution of the second metal ions throughout the MOF structure is more likely. However, if these conditions are not met, mixed MOF phases or amorphous MOFs may form. In addition to these factors, ensuring that the introduced metal cations can react concurrently to achieve successful incorporation and to avoid the formation of mixed phases of MOFs is crucial. It has been suggested that the utilization of less reactive cations can help reduce the formation of separate phases of MOFs (**Figure** **3**a), such as in the fabrication of bimetallic MIL‐53(Cr/Fe) (MIL = Materials of Institute Lavoisier), where the substitution of Fe^3+^ cations with Fe^0^ prevents the formation of mixed phases of MIL‐53.^[^ ^100^ ^]^ To date, a wide variety of bimetallic MOFs have been synthesized via this approach. Some notable examples include MnCo‐BTC,^[^ ^10^ ^]^ NiCo‐BTC,^[^ ^13^ ^]^ Zn/Cu‐BTC (BTC = 1,3,5‐benzenetricarboxylate),^[^ ^101^ ^]^ Co/MOF‐5 (Zn_4_O(BDC)_3_; BDC = 1,4‐benzenedicarboxylate),^[^ ^102^ ^]^ MIL‐101(Al/Fe)‐NH_2,_^[^ ^103^ ^]^ and CuCo‐MOF^[^ ^104^ ^]^ (Figure 3b).

#### 3.3.2 Post‐Synthetic Metal Exchange (PSME)

Postsynthetic metal exchange (PSME) or transmetalation can be employed to produce bimetallic MOFs, which cannot be directly synthesized via the one‐pot method (Figure 2b).^[^ ^105^ ^]^ This process involves breaking the coordination bonds between organic ligands and metal ions and the subsequent formation of new bonds with the introduced metal ions. Several factors influence the degree of transmetalation, including i) the ease of replacing the original metal ions, ii) the valence and coordination tendencies of the incoming metal, iii) the solvent used in the exchange process, and iv) the overall chemical stability of the resulting bimetallic MOF.^[^ ^106^ ^]^ In most cases, metal exchange is only partial, with a rapid initial phase that gradually slows as equilibrium is approached. To optimize metal exchange, solutions containing new metal ions often need to be refreshed multiple times. However, complete exchange is rarely achieved. The stability of bimetallic MOFs synthesized through this method is primarily determined by the ease with which metal ions can be exchanged.

In terms of exchangeability, Cu^2+^ ions tend to replace most second‐row transition metals (e.g., Zn^2+^, Cd^2+^, and Mn^2+^) owing to their high electronegativity (Figure 3c), which enables them to establish a greater number of covalent bonds while maintaining thermodynamic stability.^[^ ^107^, ^108^ ^]^ In contrast, Cd^2+^ and Pb^2+^ exhibit higher exchange rates than Cu^2+^ because of their lower electronegativity, which enables them to form more labile ionic bonds.^[^ ^109^ ^]^ The exchange rate can be further accelerated by using solvents with low ligand field strength and small molecular sizes, such as methanol, instead of larger solvents such as dimethylsulfoxide (DMSO) or N,N‐dimethylformamide (DMF).^[^ ^110^ ^]^

However, PSME does not always lead to complete replacement within the MOF subunits. Instead, the incoming metal ions may graft onto ligand vacancy sites or precipitate as nanoscale metal oxides on the MOF surface.^[^ ^111^, ^112^ ^]^ To confirm successful metal ion exchange within the MOF subunits, a combination of advanced analytical techniques is needed. Additionally, template‐based methods have been explored for achieving more accurate control over the metal ion distribution in bimetallic MOFs. For example, a metal‒organic polymer with well‐defined binding sites was successfully used as both a precursor and structural template for synthesizing bimetallic MOF‐74 (Figure 3d), allowing for controlled anchoring of a secondary metal.^[^ ^113^ ^]^

Covalent PSME offers an efficient pathway for achieving bimetallic MOFs that are challenging or nearly impossible to obtain via the one‐pot synthesis method. The speed of the metal exchange process can be very rapid or sluggish (>1 week), depending on the size, reactivity, electronegativity, and other characteristics of the injected metal ions. Despite its importance, only a few studies have systematically examined the variables that govern the kinetics and thermodynamics of this process. A notable study by Song et al. identified these variables.^[^ ^114^ ^]^ They investigated the impact of solvent selection, immersion solution concentration, and reaction temperature on the metal exchange of Cu^2+^ by Zn^2+^ at HKUST‐1 (Hongkong University of Science and Technology‐1). The Zn counterpart of HKUST‐1 was synthesized and then submerged in a methanol solution containing Cu(NO_3_)_2·_2.5H_2_O at room temperature. While successful metal exchange was observed in methanol, no exchange occurred when the methanol was replaced with DMF, likely due to sluggish reaction kinetics at room temperature. However, transmetalation proceeded in DMF at elevated temperatures. The study also revealed that the flexibility of the skeletal metal center and the ligand's chemical environment influenced the metal transfer kinetics. Variations in site reactivity led to the formation of core–shell heterostructures. Notably, while Cu^2+^ was successfully exchanged with Zn^2+^, the reverse transmetalation process did not occur, highlighting the directional nature of the metal exchange in this system.

Das and co‐workers reported a complete and reversible exchange between Cd^2+^ and Pb^2+^ ions in the skeleton of a Cd‐MOF in aqueous solution at room temperature, demonstrating a single‐crystal‐to‐single‐crystal transition that preserved both the crystallinity and structural integrity of the framework.^[^ ^115^ ^]^ Over the course of one week, Pb^2+^ fully replaced Cd^2+^ in the metal exchange process. Inductively coupled plasma‐atomic emission spectroscopy (ICP‒AES) analysis revealed that ≈50% of the Cd^2+^ ions were exchanged within the first day, whereas the complete reversal process took more than three weeks.

In general, MOFs with unstable metal‒ligand bonds are the primary targets for PSME.^[^ ^109^, ^116^ ^]^ However, metal exchange has also been observed in structurally robust MOFs, such as ZIF‐, UiO‐, and MIL‐based MOFs (ZIF = Zeolitic imidazolate framework; UiO = Universitetet i Oslo). This finding indicates the versatility of postsynthetic modifications for obtaining bimetallic MOFs. Despite this potential, precise control over the reaction parameters and extended reaction times are often required to achieve successful and complete exchange of metal ions.

### 3.4 Exterior Nanoarchitecturing of Bimetallic MOFs

Exterior nanoarchitecturing typically involves the morphological tuning of bimetallic MOFs into low‐dimensional (0D to 2D) or 3D structures. This type of morphology control is important because it directly affects their physical and chemical properties, as well as their sensing performance. Low‐dimensional bimetallic MOFs integrate the inherent advantages of bimetallic MOFs (e.g., large surface area, high porosity, and tunable electronic structure) with the unique characteristics of low‐dimensional nanomaterials (e.g., high aspect ratio, increased surface area, and abundant accessible active sites). These features make low‐dimensional bimetallic MOFs superior to bulk MOF crystals for sensing applications. Specifically, their larger surface area offers more sites for gas molecule adsorption, whereas their reduced size minimizes diffusion distances, enabling faster response and recovery times.^[^ ^117^ ^]^

Low‐dimensional bimetallic MOFs can be categorized into three classes, namely, 0D, 1D, and 2D bimetallic MOFs, on the basis of their nanoscale confinement. In addition, bimetallic MOFs with 3D structures, ranging from octahedral structures to complex hierarchical structures, have also been reported.^[^ ^118^, ^119^ ^]^ Hierarchical 3D MOFs often self‐assemble from 1D or 2D structures, resulting in a high surface area, increased interparticle connectivity, and improved structural stability.

#### 3.4.1 Zero‐Dimensional (0D) Bimetallic MOFs

**Figure 4:**
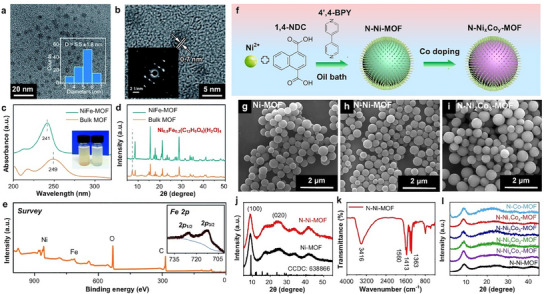
a,b) Low‐ (inset: size distribution histogram) and b) high‐magnification (inset: selected area electron diffraction pattern) transmission electron microscopy (TEM) images of NiFe‐MOF nanodots obtained through mechanical grinding and ultrasonication of bulk NiFe‐MOF in an aqueous solution. Comparison of c) UV‒vis spectra and d) XRD patterns of bulk NiFe‐MOF and NiFe‐MOF nanodots. e) XPS survey spectrum of NiFe‐MOF nanodots. Reproduced with permission.^[^ ^120^ ^]^ Copyright 2020, Royal Society of Chemistry. f) Schematic illustration of the preparation process for N‐Ni*_x_* Co*_y_*_‐_MOF nanospheres. Scanning electron microscopy (SEM) images of g) Ni‐MOF, h) N‐Ni‐MOF, and i) N‐Ni_2_Co_3_‐MOF. j) XRD patterns of Ni‐MOF and N‐Ni‐MOF. k) FTIR spectrum of N‐Ni‐MOF. l) XRD patterns of N‐Ni*_x_* Co*_y_*‐MOF obtained using various Ni/Co ratios. Reproduced with permission.^[^ ^123^ ^]^ Copyright 2023, Springer Nature.

0D nanostructures refer to nanostructures that are confined in all three spatial dimensions, leading to structures with nanometer‐scale dimensions in all directions. They typically include quantum dots, nanoparticles (NPs), and nanospheres. 0D nanostructures exhibit unique electronic and optical properties owing to quantum confinement effects. Although 0D structures are relatively rare in bimetallic MOFs, some researchers have successfully developed synthetic methods to achieve them. For example, Zhao et al. fabricated bimetallic NiFe‐MOF nanodots via a two‐step method. In the first step, Ni and Fe salts were combined with 2,6‐naphthalenedicarboxylic acid dipotassium (as the organic ligand) in an aqueous solution.^[^ ^120^ ^]^ Next, the bulk NiFe‐MOF was converted into NiFe‐MOF nanodots via mechanical grinding and ultrasonication. During ultrasonication, bubble generation and collapse (“cavitation”) facilitate the breakdown of organic and inorganic layers, yielding nanodots with a controlled size and morphology. The resulting NiFe‐MOF nanodots had an average diameter of ≈5.5 nm (**Figure** **4**a,b) and a surface area five times greater than that of bulk NiFe‐MOF (21 vs 4.0 m^2^ g^−1^). Notably, these nanodots featured both intrinsic micropores (0.7 and 1.2 nm) and mesopores (9–38 nm), whereas the bulk NiFe‐MOF was predominantly microporous (<2 nm). The significantly smaller size of these NiFe‐MOF nanodots shortened the charge transport pathway through quantum confinement effects, as evidenced by an 8 cm^−1^ downshift in the ultraviolet‒visible (UV‒vis) absorption peak relative to that of the bulk counterpart (Figure 4c). X‐ray diffraction (XRD) analysis of these nanodots revealed the disappearance of the peak at 7.2° (Figure 4d), indicating structural downsizing and the breakdown of alternating metal‒organic carbon layers. Moreover, X‐ray photoelectron spectroscopy (XPS) confirmed the successful incorporation of Fe into the Ni‐MOF framework, as evidenced by the appearance of Fe 2p peaks in the NiFe‐MOF nanodots (Figure 4e). In addition, the transition from bulk NiFe‐MOF to nanodots resulted in improved solubility in aqueous solutions, further underscoring the benefits of size reduction.

Bimetallic Ni–Co MOF nanospheres with an average diameter of 400 nm were previously prepared through a one‐pot solvothermal method.^[^ ^121^ ^]^ This process involved mixing Ni and Co salts with the BTC ligand and polyvinylpyrrolidone (PVP), followed by a reaction at 150 °C for 24 h. While nanosphere formation was successfully achieved, the exact role of PVP in controlling the 0D structure remains unclear. This contrasts with the findings of Young et al., where PVP was identified as a crucial shape‐controlling agent in the synthesis of NiCo‐MOF‐74 nanospheres.^[^ ^122^ ^]^ In their study, Ni and Co precursors were combined with 2,5‐dihydroxyterephthalic acid (as the organic ligand) and PVP in DMF, followed by solvothermal treatment at 100 °C for 24 h. The obtained NiCo‐MOF‐74 product was isostructural to Co‐MOF‐74, with similar diffraction peaks and relative intensities. Moreover, the NiCo‐MOF‐74 nanospheres presented a greater SSA (729.5 m^2^ g^−1^) than did the pure Co‐MOF‐74 nanorods (600.6 m^2^ g^−1^) because of their smaller particle size and increased microporosity. Lin and co‐workers reported the synthesis of uniform nitrogen‐doped Ni*_x_* Co*_y_*‐MOF (N‐Ni*_x_* Co*_y_*‐MOF) nanospheres via a two‐step process, as illustrated in Figure 4f.^[^ ^123^ ^]^ In the first step, nitrogen‐doped Ni‐MOF (N‐Ni‐MOF) nanospheres were prepared by reacting a solution containing Ni^2+^ (as the metal node), 1,4‐naphthalenedicarboxylate (1,4‐NDC) (as the organic ligand), and 4′,4‐bipyridine (4′,4‐bpy) in an oil bath at 120 °C for 12 h. In the second step, N‐Ni*_x_* Co*_y_*‐MOF nanospheres with different Ni/Co ratios were prepared by varying the amount of the Co precursor incorporated into the reaction. Compared with those of the Ni‐MOF and N‐Ni‐MOF nanospheres (Figure 4g,h), the bimetallic N‐Ni_2_Co_3_‐MOF nanospheres were relatively larger in size, with an average diameter of ≈400 nm (Figure 4i). Despite the increase in size, nitrogen doping into the Ni‐MOF framework did not disrupt its crystal structure, as depicted in Figure 4j. Further validation was provided by Fourier transform infrared (FTIR) spectroscopy analysis of the N‐Ni‐MOF nanospheres, which revealed successful coordination bonding between Ni^2+^ and the carboxyl groups within the 1,4‐NDC ligand (Figure 4k). However, the introduction of Co into the N‐Ni‐MOF led to a gradual shift in the (100) peak at ≈8.7°, particularly with increasing Co content (Figure 4l).

Although bimetallic MOFs with 0D structures (e.g., MOF NPs and quantum dots) exhibit some unique physicochemical properties, they are generally less favorable than 1D and 2D structures because of their lower surface contact, weaker particle connectivity, poorer stability, and greater aggregation tendency. To mitigate these issues, researchers have explored alternative low‐dimensional bimetallic MOFs, including 1D and 2D bimetallic MOFs.

#### 3.4.2 One‐Dimensional (1D) Bimetallic MOFs

One‐dimensional nanostructures are defined as nanostructures that are confined in two spatial dimensions, allowing free movement along one axis. Common examples include nanorods, nanofibers, nanowires, nanotubes, and nanobelts. These structures are typically designed at the nanoscale but can extend to micron‐sized lengths while maintaining nanometer‐scale widths. Typically, two criteria are used to classify a nanostructure as 1D: i) two dimensions are below 100 nm, and ii) one dimension exceeds 100 nm. Bimetallic MOFs with 1D nanostructures provide improved signal transduction due to their higher aspect ratio and good mechanical flexibility, making them ideal candidates for wearable or flexible sensors.^[^ ^124^, ^125^ ^]^ Additionally, their elongated morphology enhances the contact area between the sensing material and gas molecules, improving gas adsorption and overall sensing performance.

To date, 1D bimetallic MOFs have been prepared through both template‐free (typically one‐pot synthesis methods) and template‐assisted approaches. Among these methods, the one‐pot synthesis method is the most widely used because of its simplicity and straightforward process. Bimetallic Co*_x_* Ni*_y_*‐MOFs with various Co/Ni ratios (*x*/*y* = 1:1, 1:5, 1:10, 1:15, and 1:20) were successfully fabricated by directly mixing Ni and Co salts with 3,5‐pyridinedicarboxylic acid (3,5‐H_2_pdc) and potassium hydroxide at ambient temperature.^[^ ^126^ ^]^ The Co/Ni ratio had only a minor effect on the morphology, as the resulting Co*_x_* Ni*_y_*‐MOFs consistently maintained a rod‐like shape across all ratios. However, the diameter range of the rods decreased from 700–800 nm to 300–400 nm as the Co/Ni ratio increased. Notably, the prepared bimetallic Co*_x_* Ni*_y_*‐MOFs remained isostructural to Co‐MOF at all tested Co/Ni ratios, suggesting that Co^2+^ ions were partially replaced by Ni^2+^ ions. This partial substitution was driven by the relatively similar ionic radii of Co^2+^ (72 pm) and Ni^2+^ (78 pm) ions.

**Figure 5:**
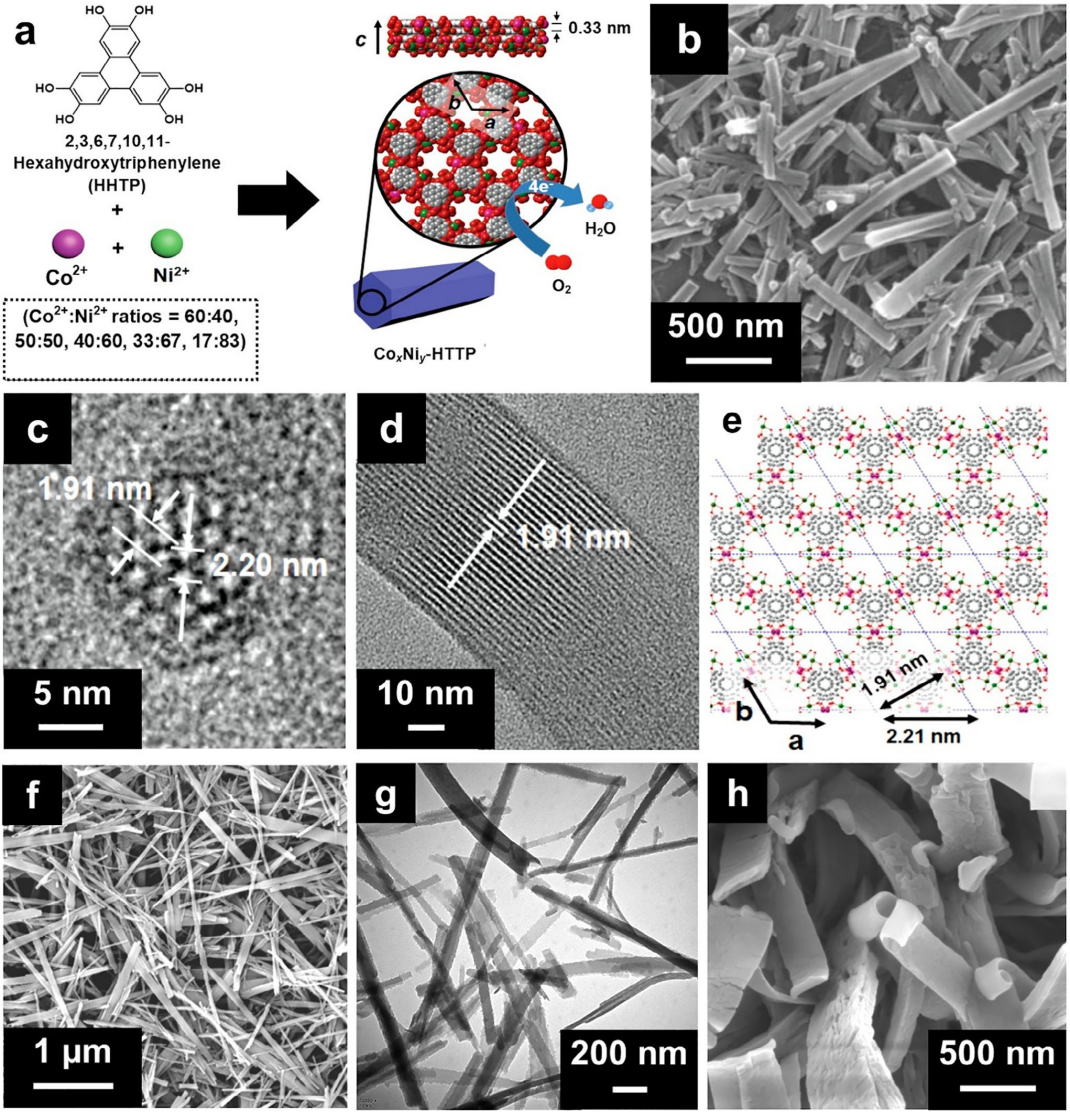
a) Schematic illustration of the synthesis process for bimetallic Co*_x_* Ni_y_‐HHTP nanorods. b) SEM and c,d) HRTEM images of Co_0.27_Ni_0.73_‐HHTP and e) the related chemical structure. Reproduced with permission.^[^ ^127^ ^]^ Copyright 2019, Wiley‐VCH. f) SEM and g) TEM images of NiCo‐BPDC nanorods obtained through a direct hydrothermal reaction between Ni and Co salts and the BPDC ligand at 170 °C for 12 h. Reproduced with permission.^[^ ^129^ ^]^ Copyright 2019, Elsevier. h) SEM image of NiCo‐BTC nanobelts achieved by aging a water/ethanol mixture containing Ni and Co precursors and the BTC ligand at ambient temperature for 2 h. Reproduced with permission.^[^ ^13^ ^]^ Copyright 2022, Elsevier.

Yoon et al. demonstrated the synthesis of a series of bimetallic Co*_x_* Ni*_y_*‐HHTP nanorods (HHTP = 2,3,6,7,10,11‐hexahydroxytriphenyl) with varying Co^2+^/Ni^2+^ ratios through a hydrothermal reaction of HHTP with different amounts of cobalt acetylacetonate [Co(OAc)_2_] and nickel acetylacetonate [Ni(OAc)_2_] at 85 °C for 12 h.^[^ ^127^ ^]^ These Co*_x_* Ni*_y_*‐HHTP MOFs exhibited a 2D layered hexagonal structure with well‐developed pores along the *c*‐axis, as illustrated in **Figure** **5**a. Furthermore, the Co_0.27_Ni_0.73_‐HHTP product displayed a hexagonal rod‐like morphology with lengths of ≈0.8–1 µm (Figure 5b). High‐resolution transmission electron microscopy (HRTEM) analysis of this bimetallic MOF revealed a hexagonal pattern in the *ab* plane and linear patterns aligned with the *c*‐axis, both of which are characteristic features of metal‐HHTP MOFs (Figure 5c). Additionally, the Co_0.27_Ni_0.73_‐HHTP MOF exhibited *d*‐spacing values of 2.20 and 1.91 nm (Figure 5c,d), with 2.20 nm *d*‐spacing corresponding to the distance between two hexagonal pores and *a* cell parameter, as represented by the chemical structures shown in Figure 5e. CuCo‐MOF‐74 hexagonal rod‐like particles were previously obtained from the direct solvothermal reaction of Cu and Co salts (Cu:Co = 1:1) with 2,5‐dihydroxyterephthalic acid (DHTP) in a DMF/ethanol mixture at 150 °C for 24 h.^[^ ^128^ ^]^ These particles had lengths ranging from 1.0–7.0 µm and thicknesses of 0.5–3.0 µm, along with a high SSA of 478.1 m^2^ g^−1^, with mesopores as the dominant type of porosity. In contrast, the CuCo‐MOF‐74 products synthesized at 110 °C and 130 °C had significantly lower SSA values of 3.98 and 6.90 m^2^ g^−1^, respectively, highlighting the importance of temperature control for optimizing the SSA and porosity of bimetallic MOFs. This substantial decrease in SSA resulted from a dramatic increase in pore diameter (from 2.53 nm to 14.78 and 7.77 nm, respectively) and a reduction in pore volume (from 0.3026 cm^3^ g^−1^ to 0.0147 and 0.0134 cm g^−1^, respectively). The bimetallic CuCo‐MOF‐74 remained isostructural to Co‐MOF‐74, and the incorporation of Cu^2+^ ions did not alter the crystal structure of Co‐MOF‐74. Similarly, NiCo‐BPDC (BPDC = 4,4′‐biphenyldicarboxylate) nanorods (Figure 5f,g) prepared via a direct hydrothermal reaction of Ni and Co salts with the BPDC ligand at 170 °C for 12 h presented a diffraction pattern identical to that of pure Co‐BPDC, indicating the minimal influence of Ni^2+^ on the crystal structure of the MOF.^[^ ^129^ ^]^

In another study, NiFe‐MOF nanorods were fabricated through the direct hydrothermal reaction of Ni and Fe precursors with a 2‐aminoterephthalic acid (NH_2_‐BDC) ligand in a water/ethanol mixture at 130 °C for 10 h.^[^ ^130^ ^]^ Ni^2+^ ions were uniformly incorporated into the Ni/Fe framework owing to the relatively similar ionic radii of the two metals. These NiFe‐MOF nanorods had an SSA of 78.2 m^2^ g^−1^ and relatively uniform mesoporosity, with a narrow pore size distribution in the range of 4–6 nm. The rich mesopores coupled with the high surface area of this bimetallic MOF provided an increased number of active sites and facilitated the effective diffusion of target gas molecules. Ultrathin NiCo‐MOF nanobelts were also synthesized via a direct solvothermal reaction between Ni and Co precursors with glutaric acid in a water/ethanol mixture at 180 °C for 48 h.^[^ ^131^ ^]^ The Ni/Co ratio plays a key role in determining the morphology of the NiCo‐MOF products. A higher Ni content promoted the formation of thinner and longer nanobelts; however, when the Ni content was too low, the nanobelts disintegrated. The NiCo‐MOF nanobelts synthesized with Ni/Co ratios of 3:1 (NiCo‐MOF‐31), 4:1 (NiCo‐MOF‐41), 1:1 (NiCo‐MOF‐11), and 2:1 (NiCo‐MOF‐21) presented SSAs of 73, 44, 41, and 12 m^2^ g^−1^, respectively, with average pore diameters of 3.71, 3.13, 3.31, and 2.97 nm. In contrast to these studies, Nguyen and colleagues demonstrated a microwave‐assisted approach (with a power of 300 W and a temperature of 100 °C) for synthesizing spindle‐like NiFe‐MOF particles with Ni^2+^/Fe^3+^ molar ratios ranging from 0–50%.^[^ ^132^ ^]^ Their findings revealed that nearly all NiFe‐MOF samples displayed diffraction patterns identical to those of pure Fe‐MOF (suggesting an isostructural nature), except for the 50% Ni/Fe‐MOF sample. Furthermore, a possible substitution of the Fe^3+^ nodes in the Fe‐MOF backbone by Ni^2+^ was observed with increasing Ni^2+^/Fe^3+^ ratio, as evidenced by a leftward shift in the main XRD peak at 9°. In addition, the symmetric vibration of the carboxyl group shifted to a lower wavenumber and split into two peaks for the 50%Ni/Fe‐MOF sample, likely due to differences in the nuclear charge and ionic radius between Ni^2+^ and Fe^3+^.

Notably, many researchers have relied on elevated temperatures (80–200 °C) and/or high pressures (typically using hydrothermal or solvothermal methods) to synthesize 1D bimetallic MOFs. As such, the development of low‐temperature methods is highly desirable for enabling the scalable production of these materials. Chowdhury and co‐workers successfully fabricated 1D bimetallic NiCo‐BTC nanobelts (Figure 5h) by aging a solution containing Ni and Co salts with the BTC ligand at room temperature.^[^ ^13^ ^]^ The Ni/Co ratio plays a key role in determining the morphology of the resulting NiCo‐BTC products, with a relatively high Ni content favoring the formation of NiCo‐BTC nanobelts (≈1–4 µm in length) over nanoplates. Bimetallic AgCu‐BTC nanorods were prepared by combining Cu and Ag precursors with the BTC ligand in a DMF/water mixture and allowing the mixture to age at room temperature for 24 h.^[^ ^133^ ^]^ The successful coordination between Cu‐Ag and the BTC ligand was confirmed by a 0.3 eV shift in the peak of the carboxyl group in the high‐resolution C 1 s spectrum of the AgCu‐BTC nanorods compared with that of the free BTC ligand. These nanorods exhibited an SSA of 27.2 m^2^ g^−1^ with an average pore diameter of 3.8 nm and a pore volume of 0.082 cm^3^ g^−1^.

In addition to the aging method, bimetallic MOFs with 1D structures have also been prepared via other low‐temperature methods. For example, bimetallic NiCo‐HHTP nanorods were previously synthesized via an electrochemical approach, where a Ni sheet served as the anode and the electrolyte was formed by dissolving HHTP in an ethanol/water mixture, followed by the addition of Co(NO_3_)_2_·6H_2_O and ammonia water (NH_3_·H_2_O).^[^ ^134^ ^]^ Various parameters, including the amount of NH_3_·H_2_O, Co^2+^ dosage, and deposition potential, strongly affect the formation of this bimetallic MOF. An optimal NH_3_·H_2_O volume of 4.5 mL was necessary to achieve highly crystalline NiCo‐HHTP MOF. In contrast, excessive NH_3_·H_2_O (≥6.5 mL) accelerated ligand deprotonation, hindering coordination with the metal ions and limiting crystal growth. Increasing the Co^2+^ dose from 20 to 80 µmol caused a slight decrease in the crystallinity of the NiCo‐HHTP MOF, although the XRD peak positions remained similar. Moreover, a deposition potential of 5 V yielded the most crystalline product. Increasing the deposition potential above 6 V reduced the crystallinity due to the excessive release of metal ions, which caused overly fast coordination with the HHTP ligand. In a separate study, Oh's group utilized a ball‐milling technique for the scalable production of bimetallic Co_0.23_Ni_0.77_‐HHTP nanorods.^[^ ^127^ ^]^ Co(OAc)_2_, Ni(OAc)_2_, and HHTP powders were mixed with a small amount of water in a stainless steel ball‐mill reactor containing five balls operated at 20 Hz for 4 h. The resulting nanorods, measuring ≈200–500 nm in length, exhibited diffraction peaks similar to those of Co‐HHTP, confirming their isostructural nature.

In addition to template‐free methods, a template‐based approach was developed by Shen et al. to fabricate bimetallic CoFe‐MOF nanoneedles on Ni foam using cobalt hydroxide (Co(OH)_2)_ nanoneedles as a template.^[^ ^135^ ^]^ Co(OH)_2_ nanoneedles were initially prepared through a hydrothermal reaction involving a Co salt, urea, and ammonium fluoride in water at 120 °C for 8 h. These nanoneedles were then subjected to a solvothermal reaction with iron nitrate and 4,5‐imidazoledicarboxylic acid (H_3_IMDC) at 140 °C for 24 h to convert them into CoFe‐MOF nanoneedles. Compared with the pristine Fe‐MOF (5.7 m^2^ g^−1^), the formed CoFe‐MOF nanoneedles presented a significantly greater SSA (67.8 m^2^ g^−1^). While template‐based methods can yield bimetallic MOFs with well‐defined 1D structures and tailored compositions, they are typically complex and multistep because of the requirement for template removal, which limits their scalability.

#### 3.4.3 Two‐Dimensional (2D) Bimetallic MOFs

2D bimetallic MOFs exhibit unique physicochemical characteristics originating from electronic effects induced by their ultrathin structure, large surface area, and high surface‐to‐volume ratio.^[^ ^136^ ^]^ In sensing applications, this high surface‐to‐volume ratio offers an increased number of adsorption sites for gas molecules. Furthermore, the highly exposed surfaces of 2D bimetallic MOFs improve access to interior active sites, thereby enhancing interactions with gas molecules.^[^ ^137^ ^]^ These features collectively lead to improved sensitivity in gas‐sensing applications.

However, synthesizing 2D bimetallic MOFs is typically challenging, as it requires restricting crystal growth to the nanometer scale in the vertical direction while maintaining unrestricted growth in lateral dimensions.^[^ ^137^, ^138^, ^139^ ^]^ Additionally, the incorporation of new secondary metal nodes can result in brittle frameworks with unpredictable topologies and functionalities, thus necessitating precise control over synthetic parameters. Top‐down methods, such as delamination, mechanical exfoliation, sonication exfoliation, and chemical exfoliation, can yield high‐quality single‐ or few‐layer nanosheets but are usually time‐consuming and yield‐limited.^[^ ^140^, ^141^ ^]^ Conversely, bottom‐up approaches, such as interfacial synthesis, three‐layer synthesis, surfactant‐assisted synthesis, modulated synthesis, and sonication synthesis, are more scalable and straightforward but often face challenges in achieving precise morphology control.^[^ ^142^, ^143^ ^]^

To date, numerous studies have reported one‐pot methods for achieving bimetallic MOFs with 2D structures and various bimetal centers, including Ni‐Co, Zn‐Co, Ni‐Mn, and Co‐Fe systems.^[^ ^13^, ^144^, ^145^ ^]^ For example, bimetallic CoZn(n)‐MOF nanosheets were prepared via a one‐pot method involving the aging of an aqueous mixture containing Co(NO_3_)_2_, Zn(NO_3_)_2_, and 2‐methylimidazole (2‐MeIM) at room temperature.^[^ ^144^ ^]^ Additionally, 2D bimetallic Ni_1‐_ *_x_* Fe*_x_* BDC MOFs with different Ni/Co ratios, namely, Ni_0.75_Fe_0.25_ BDC, Ni_0.5_Fe_0.5_ BDC, and Ni_0.25_Fe_0.75_ BDC, have been fabricated by refluxing a solution containing the BDC ligand and nickel and ferric chlorides for 24 h.^[^ ^146^ ^]^ In this process, triethylamine (TEA) serves as an acid‐binding agent to positively influence chemical equilibrium. Notably, the addition of Fe greatly influenced the crystal structure and morphology of the resulting NiFe‐BDC MOFs with higher Fe contents, leading to poorer crystallinity and the formation of irregular particles. In another study, a 2D bimetallic Zn/Fe‐MOF was synthesized at low temperature by reacting Fe and Zn precursors with 1,4,5,8‐naphthalenetetracarboxylic acid at 60 °C in an oil bath.^[^ ^147^ ^]^ The resulting Zn/Fe‐MOF nanosheets displayed a lateral size of <1 µm and an average thickness below 25 nm, with an SSA of 47 m^2^ g^−1^. The identical diffraction patterns of Zn/Fe‐MOF and pure Zn‐MOF implied that the incorporation of Fe had a minimal impact on the crystal structure of the Zn‐based framework.

Two‐dimensional bimetallic MOFs have also been produced via other low‐temperature methods. For example, 2D Co_3_Fe‐MOF nanosheets were obtained by direct ultrasonication of a DMF/ethanol/water mixed solution containing Co and Fe salts, an NH_2_‐BDC ligand, and TEA for 8 h.^[^ ^145^ ^]^ The obtained nanosheets had a lateral size of ≈100 nm, and their thickness increased significantly with increasing Co/Fe ratio. Furthermore, the IR spectrum of the bimetallic Co_3_Fe‐MOF resembled that of pure Co‐MOF, indicating its isostructural nature. In these bimetallic MOFs, Co and Fe atoms are coordinated octahedrally by six oxygen atoms, forming parallel, edge‐sharing chains linked by μ3‐OH groups along the [010] direction in the (200) lattice plane. These chains are interspersed with NH_2_‐BDC ligands, creating a layered structure. XPS analysis revealed that the incorporation of Fe^2+^ modified the valence state of Co^2+^, thereby affecting the electron‐accepting capability of the bimetallic MOF. NiFe‐MOF nanosheets were fabricated via a simple two‐step ultrasonication method at room temperature.^[^ ^148^ ^]^ In this process, a lamellar‐structured 2D Ni‐MOF was first prepared via a top‐down ultrasonic‐assisted exfoliation method. Following this, Fe^3^⁺ ions were introduced as secondary metal nodes, along with the BDC ligand, leading to the generation of bimetallic NiFe‐MOF. The crystallinity of the resulting 2D NiFe‐MOF nanosheets gradually decreased with increasing Fe content due to the amorphous nature of the in situ‐formed Fe‐MOF. However, the incorporation of Fe^3^⁺ ions had little effect on the coordination of the Ni‐MOF, as evidenced by the unchanged Raman peaks after modification. These 2D NiFe‐MOF nanosheets displayed a significantly greater SSA and pore volume (75.31 m^2^ g^−1^ and 0.19 cm^3^ g^−1^) than both pure Ni‐MOF (17.95 m^2^ g^−1^ and 0.11 cm^3^ g^−1^) and Fe‐MOF (10.68 m^2^ g^−1^ and 0.041 cm^3^ g^−1^). The higher SSA, larger pore volume, and narrow mesopore distribution of the 2D NiFe‐MOF provided more accessible active sites and increased the contact area, offering clear advantages over its monometallic counterparts.

**Figure 6:**
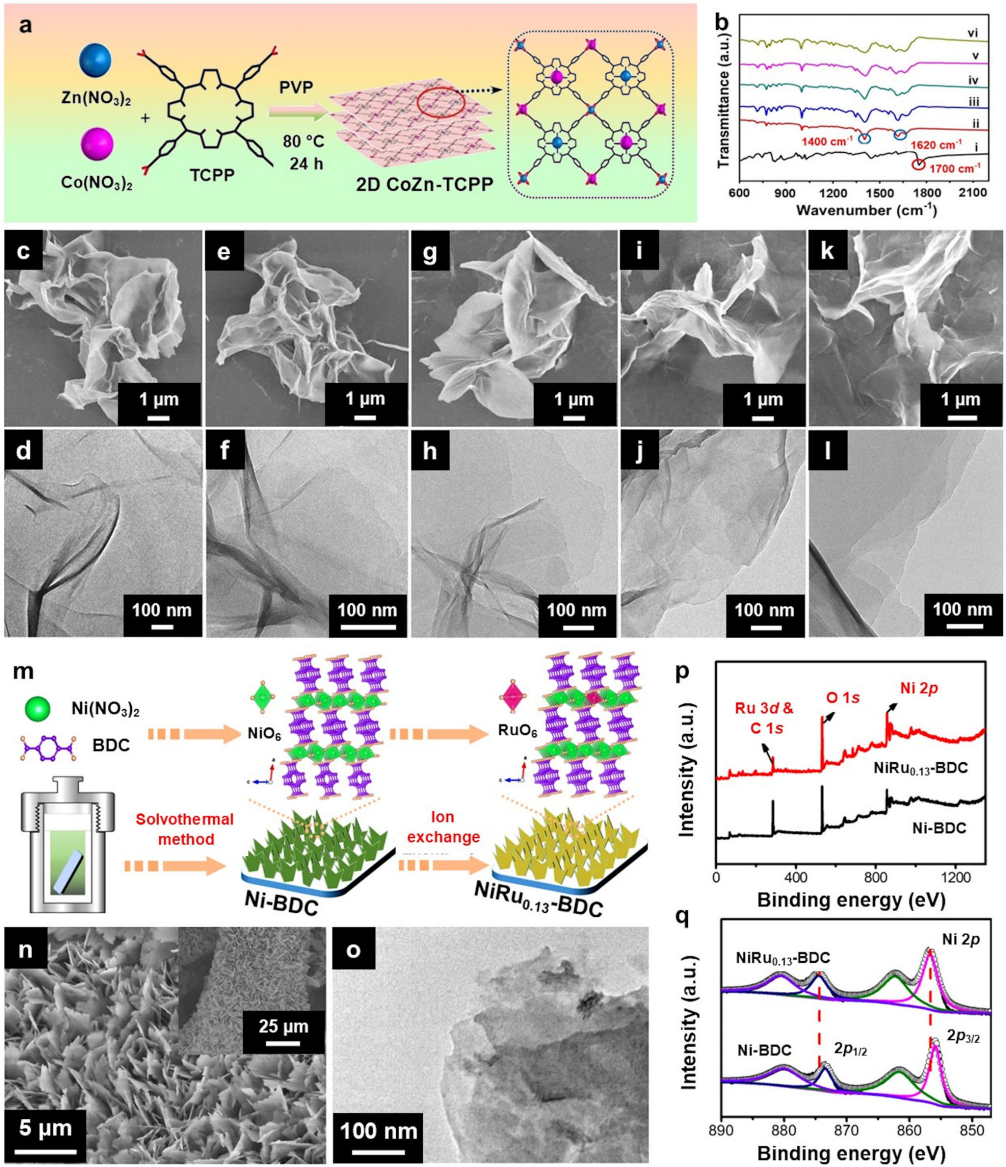
a) Schematic illustration of the synthesis of 2D CoZn‐TCPP nanosheets with varying Co/Zn ratios. b) FTIR spectra of i) TCPP, ii) Co‐TCPP, iii) Co_75_Zn_25_‐TCPP, iv) Co_50_Zn_50_‐TCPP, v) Co_25_Zn_75_‐TCPP, and vi) Zn‐TCPP. SEM and TEM images of c,d) Co‐TCPP, e,f), Co_75_Zn_25_‐TCPP, g,h) Co_50_Zn_50_‐TCPP, i,j) Co_25_Zn_75_‐TCPP, and k,l) Zn‐TCPP. Reproduced with permission.^[^ ^149^ ^]^ Copyright 2021, Springer Nature. m) Schematic illustration depicting the synthesis process of NiRu_0.13_‐BDC nanosheets and the corresponding n) SEM and o) TEM images. p) XPS survey spectra and q) high‐resolution Ni 2p spectra of Ni‐BDC and NiRu_0.13_‐BDC. Reproduced with permission.^[^ ^156^ ^]^ Copyright 2021, Springer Nature.

Although several low‐temperature methods have been reported, the one‐pot synthesis of 2D bimetallic MOFs often requires high temperatures (above 80 °C) and/or high pressure. For example, Ma and co‐workers synthesized a series of 2D Co*x*Zn_100‐_ *_x_*‐TCPP MOFs (TCPP = tetrakis(4‐carboxyphenyl)porphyrin) by preparing DMF/ethanol solutions with varying molar ratios of Co(NO_3_)_2_·6H_2_O and Zn(NO_3_)_2_·6H_2_O (*x*  =  100, 75, 50, 25, 0), followed by the addition of PVP and TCPP and subsequent heating at 80 °C for 24 h (**Figure** **6**a).^[^ ^149^ ^]^ The formation of the Co*_x_* Zn_100‐_ *_x_* TCPP MOF was verified via FTIR spectroscopy, as supported by the absence of the C = O peak at 1700 cm^−1^ and the emergence of two new peaks at 1620 and 1400 cm^−1^ (Figure 6b), indicating successful coordination of the carboxyl groups in TCPP with the metal centers. The resulting Co*_x_* Zn_100‐_ *_x_*‐TCPP MOFs exhibited a wrinkled sheet‐like morphology (Figure 6c–l), with an average thickness of ≈4–5 nm for Co_25_Zn_75_‐TCPP. This ultrathin structure was promoted by PVP, which restricted the vertical stacking of the Co*_x_* Zn_100‐_ *_x_* TCPP layers. In a related study, CuMn‐TCPP nanosheets were fabricated by heating a mixture of MnCl_2_·4H_2_O and Cu‐TCPP in DMF at 90 °C overnight.^[^ ^150^ ^]^ The Cu content in the bimetallic CuMn‐TCPP nanosheets (11.83%) was lower than that in the Cu‐TCPP nanosheets (14.13%), suggesting the partial replacement of Cu^2+^ by Mn^2+^. Extended fine X‐ray absorption fine spectroscopy (EXAFS) analysis revealed that the Mn atoms were tetrahedrally coordinated to four oxygen atoms (Mn–O_4_).

Bimetallic ZnPd‐TCPP nanosheets were synthesized through a solvothermal reaction between Pd‐TCPP and Zn(NO_3_)_2_·6H_2_O in a mixed solvent of DMF and ethanol at 80 °C for 24 h.^[^ ^151^ ^]^ In this 2D bimetallic MOF, each Zn node is coordinated to two hydroxyl oxygen atoms and two carbonyl oxygen atoms derived from the carboxyl groups of four TCPP(Pd) ligands. The obtained ZnPd‐TCPP nanosheets displayed a relatively high SSA of 125.6 m^2^ g^−1,^ with an average pore size of 2.3 nm and a pore volume of 0.08 cm^3^ g^−1^. They were predominantly microporous, with micropore peaks at 0.8 and 1.2 nm. Unlike these reports, Ji et al. fabricated TbEu‐TCPP nanosheets via a microwave‐assisted method.^[^ ^152^ ^]^ The procedure involved preparing a solution of Tb(NO_3_)_3_·6H_2_O, Eu(NO_3_)_3_·6H_2_O, TCPP, and acetic acid in DMF, followed by heating in a microwave oven at ≈231 W. Structurally, Tb and Eu ions coordinated exclusively with the oxygen atoms of the carboxyl groups, forming Tb/Eu–O bonds, whereas no bonding occurred between Tb/Eu and the nitrogen atoms at the center of the porphyrin ring. Compared with the bulk TbEu‐TCPP prepared via a solvothermal approach (313.86 m^2^ g^−1^), the microwave‐synthesized TbEu‐TCPP nanosheets presented a larger SSA (332.13 m^2^ g^−1^), highlighting the advantages of the 2D structure.

Moreover, 2D bimetallic MOFs can be directly grown on substrates to reduce their fabrication complexity, increase their stability, and improve their scalability and processability.^[^ ^153^, ^154^, ^155^ ^]^ In particular, the direct growth of MOFs on conductive substrates provides a simple and effective strategy to address their low electrical conductivity and reinforce structural integrity. For example, MIL‐53 (FeNi) nanosheets were grown on Ni foam by immersing the foam in a DMF/ethanol/water solution containing FeCl_2_ and BDC, followed by heating at 125 °C for 12 h.^[^ ^155^ ^]^ During this process, the BDC ligand coordinated with externally supplied Fe^2+^ ions and Ni^2+^ ions liberated from the Ni foam, leading to the formation of MIL‐53 (FeNi). This method eliminates the need to introduce Ni^2+^ ions externally via dissolved nickel salts. Sun and co‐workers fabricated a NiRu_0.13_‐BDC nanosheet array on Ni foam via a combination of solvothermal and ion exchange methods (Figure 6m–o).^[^ ^156^ ^]^ The synthesis process involved initially growing a Ni‐BDC nanosheet array on Ni foam via a solvothermal reaction between Ni(NO_3_)_2_·6H_2_O and BDC at 100 °C for 15 h, followed by a second solvothermal reaction with RuCl_3_ in ethanol at 80 °C for 12 h. Successful incorporation of Ru was evidenced by the appearance of the Ru 3d peak in the XPS survey spectrum of NiRu_0.13_‐BDC (Figure 6p). Additionally, shifts in the Ni 2p_1/2_ and Ni 2p_3/2_ peaks to higher binding energies (Figure 6q) suggested strong electron interactions between Ni and Ru atoms, along with electron depletion on Ni. These examples highlight the versatility and effectiveness of substrate‐supported strategies for constructing 2D bimetallic MOFs with better conductivity, enhanced structural stability, and improved processability.

#### 3.4.4 Three‐Dimensional (3D) Bimetallic MOFs

3D bimetallic MOFs exhibit significant structural diversity, adopting a wide range of morphologies, from simple cubic structures to complex hierarchical architectures. In particular, 3D hierarchical bimetallic MOFs, composed of 1D or 2D subunits, offer high SSAs, strong interparticle connectivity, excellent mechanical properties, and superior structural stability. These features increase the exposure and availability of active sites for gas adsorption, improve long‐term stability, and facilitate gas molecule diffusion during sensing.

To date, several studies have demonstrated the synthesis of bimetallic MOFs with diverse 3D nanostructures via hydrothermal or solvothermal methods. For example, a MnLa‐MOF with an octahedral morphology was previously prepared through a direct hydrothermal reaction of lanthanide(III) chloride hydrates and manganese metal (as the metal precursors) with 3,5‐pyrazole dicarboxylic acid monohydrate (as the organic ligand) in water at 180 °C.^[^ ^118^ ^]^ The resulting MnLa‐MOF exhibited a three‐node 4,6,6 connected 3D spatial network with a highly symmetrical cubic crystal structure. This method was also applicable for the synthesis of bimetallic MnCe‐ and MnPr‐MOFs. The MnLa‐MOF octahedral particles ranged in size from 20 to 40 µm. Despite the method's simplicity, it has several limitations, including (i) the need for excess Mn metal to achieve high product purity and (ii) the requirement of lithium hydroxide to obtain bimetallic MnLa‐MOFs with good crystallinity. In a related report, octahedral FeCo‐MOF particles were synthesized via a hydrothermal reaction of Fe and Co powders with the BTC ligand in an acidic medium at 150 °C for 12 h.^[^ ^157^ ^]^ The resulting FeCo‐MOF particles displayed a diffraction pattern identical to that of MIL‐100(Fe), suggesting that Co incorporation had a minimal effect on the crystal structure. Additionally, they displayed a broad particle size distribution ranging from ≈200 nm to 20 µm.

Dandelion‐like NiCo‐MOFs were previously fabricated through a solvothermal approach in which a mixture of Ni and Co precursors, the BTC ligand, and PVP in a DMF/water/ethanol solvent system was heated at 150 °C for 10 h.^[^ ^52^ ^]^ In this method, the addition of Co^2+^ ions increased the density of rod‐like subunits on the surface of the dandelion‐like Ni‐Co MOFs compared with that of the pure Ni‐MOF. Notably, this incorporation also induced a shift in the crystallinity of the Ni‐MOF toward a more amorphous state, suggesting a possible substitution of Ni^2+^ by Co^2+^. A similar solvothermal approach has been employed for the in situ synthesis of 3D hierarchical hollow microspheres of NiZn‐MOF (NZMF) on carbon foam.^[^ ^158^ ^]^ The formation of these structures is governed by the Ostwald ripening process, during which nanoshells develop an internal density gradient within the solid aggregate. This transformation, driven by continuous or multistep Ostwald maturation, involves a directional growth phase regulated by the same mechanism. During the solvothermal process, the outer nanoshell layer evolves into rod‐like subunits, which subsequently assemble into a complex hollow hierarchical structure through both inward and outward Ostwald ripening.

**Figure 7:**
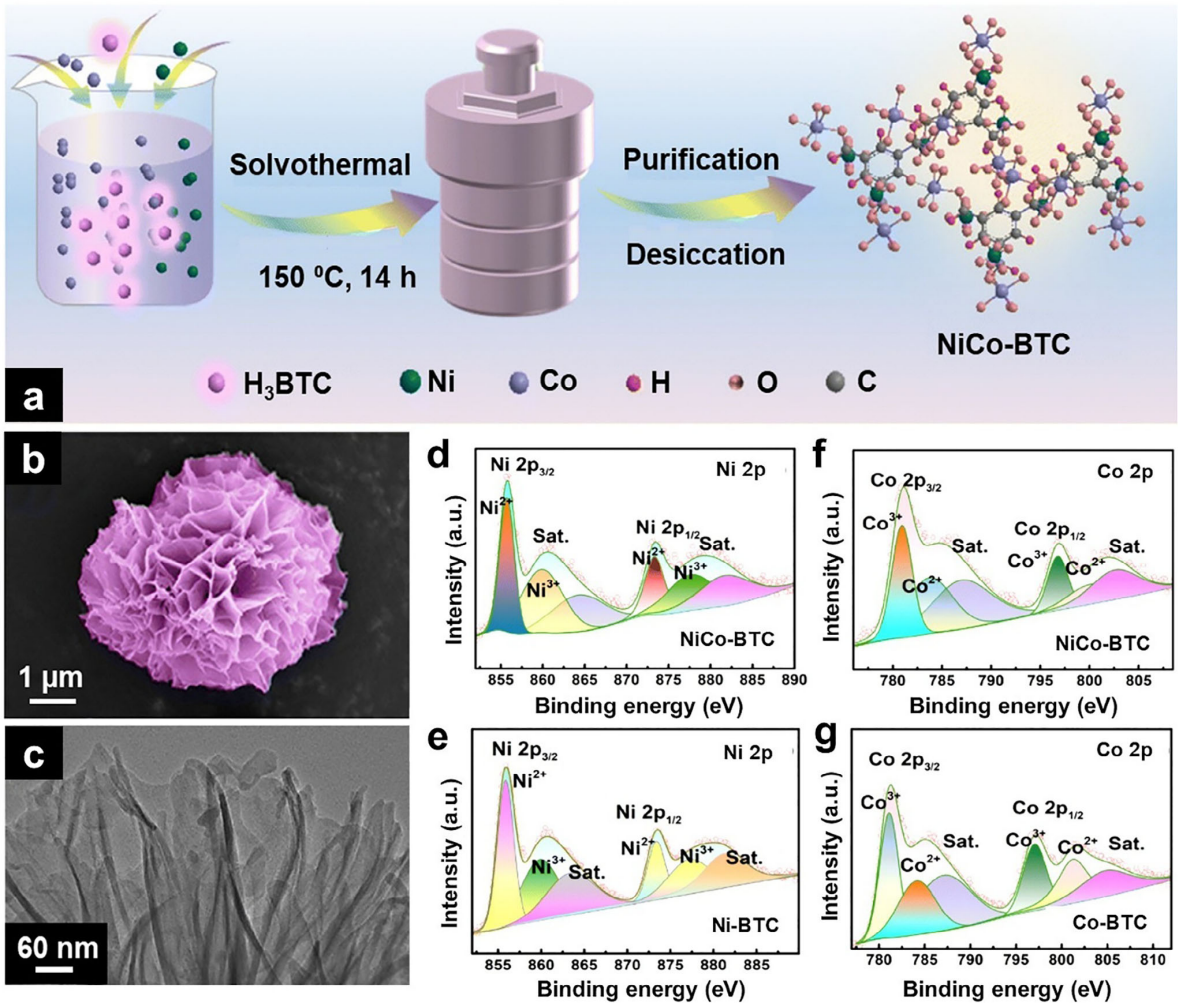
a) Schematic diagram depicting the solvothermal synthesis of a 3D flower‐like NiCo‐BTC MOF. b) SEM and c) TEM images of the hierarchical flower‐like NiCo‐BTC MOF. High‐resolution Ni 2p spectra of d) NiCo‐BTC and e) Ni‐BTC. High‐resolution Co 2p spectra of f) NiCo‐BTC and g) Co‐BTC. Reproduced with permission.^[^ ^119^ ^]^ Copyright 2023, Royal Society of Chemistry.

Hierarchical NiCo‐BTC flower‐like particles (**Figure** **7**a) were fabricated by the simple solvothermal reaction of Ni and Co salts with the BTC ligand at 150 °C for 14 h.^[^ ^119^ ^]^ These hierarchical flower‐like particles are composed of ultrathin, interconnected nanosheets with a uniform distribution of Ni and Co (Figure 7b,c). The resulting NiCo‐BTC product exhibited poorer crystallinity than pure Ni‐BTC owing to the faster coordination and crystallization rates of Co^2+^ ions relative to those of Ni^2+^ ions. Interestingly, the incorporation of Co^2+^ increased the Ni^3+^/(Ni^2+^ + Ni^3+^) ratio in the NiCo‐BTC MOF compared with that in Ni‐BTC (Figure 7d,e), which enhanced its ability to oxidize the target analyte. Furthermore, the Co species in this bimetallic MOF existed as Co^2+^ and Co^3+^ (Figure 7f,g), similar to those in pure Co‐BTC. Additionally, a facile PVP‐assisted synthesis method has been developed for obtaining bimetallic Ni‐based MOFs (FeNi‐MOFs and CoNi‐MOFs) with hierarchical flower‐like structures.^[^ ^159^ ^]^ In the synthesis process, Ni and secondary metal (Co or Fe) precursor solutions were mixed with a water/ethanol mixture containing pyrazine and PVP. The subsequent addition of K_2_[Ni(CN)_4_] to this mixture led to the precipitation of the bimetallic Ni‐based MOFs. In this case, PVP acted as a shape‐directing agent to promote the formation of hierarchical flower‐like structures with an average diameter of ≈600 nm. These hierarchical particles are composed of radially aligned 2D nanosheets with thicknesses ranging from 20 nm to 60 nm.

Unlike previous studies employing hydrothermal or solvothermal methods at elevated temperatures and pressures, Chowdhury and co‐workers successfully prepared hierarchical bimetallic MnCo‐BTC MOFs with tunable subunits and interior architectures under ambient conditions.^[^ ^10^ ^]^ These hierarchical 3D MnCo‐BTC MOFs were synthesized by directly mixing manganese and cobalt precursors with the BTC ligand in a water/ethanol solution, followed by brief aging for 2 h. By adjusting the Mn/Co ratio, the subunit morphology within the hierarchical structures could be controlled. Generally, a relatively high Mn content promoted the formation of 1D rod subunits, whereas an increased Co content favored the generation of 2D plate subunits. Moreover, compared with those of pure Mn‐BTC and Co‐BTC, the UV‒vis absorption peaks of the bimetallic MnCo‐BTC MOFs redshifted, likely due to changes in the electronic transition energy levels of the BTC ligand resulting from the incorporation of a secondary metal. In a related work, hierarchical CoNi‐MOF flower‐like particles were synthesized through a simple aging process at room temperature involving a mixed solution of cobalt acetate tetrahydrate and the BDC ligand in acetonitrile and DMF.^[^ ^160^ ^]^ The addition of Ni^2+^ ions was crucial for promoting the gradual assembly of the nanosheets into hierarchical flower‐like particles. As the Ni content increased, the intercalation of this flower‐like MOF became more confined, ultimately resulting in a compact flower‐like structure. Additionally, the introduction of Ni changed the chemical environment of Co in this bimetallic MOF, causing electron deficiency at Co centers. The SSA of the optimized CoNi‐MOF sample (Co_7_Ni_3_‐MOF) was 48.4 m^2^ g^−1^, with mesopores as the dominant pore type. However, one disadvantage of this method is the use of toxic chloroform to remove impurities.

A summary of the synthetic conditions and methods used to prepare bimetallic MOFs with various compositions and morphologies is provided in **Table** **1**.^[^ ^10^, ^11^, ^13^, ^120^, ^122^, ^132^, ^133^, ^134^, ^146^, ^151^, ^152^, ^160^, ^161^, ^162^, ^163^, ^164^, ^165^, ^166^, ^167^, ^168^, ^169^, ^170^, ^171^, ^172^, ^173^, ^174^, ^175^, ^176^, ^177^, ^178^, ^179^ ^]^

**Table 1:** Summary of the synthetic methods and conditions used for preparing bimetallic MOFs with controlled morphologies.

MOF	Organic ligand	Synthesis method	Synthesis conditions	SSA [m^2^ g^−1^]	Pore volume [cm^3^ g^−1]^	Refs.
Hierarchical MnCo‐BTC spheres	BTC	Aging	RT for 2 h	12.8	N/A	[10]
ZnCo‐BTC microrods	BTC	Aging	RT for 24 h	N/A	N/A	[11]
Hollow NiCo‐BTC nanoplates	BTC	Aging	RT for 2 h	10.7	0.072	[13]
Flower‐like CoNi‐MOF	BDC	Aging	RT for 24 h	48.4	N/A	[160]
Cu/Ce‐MOF nanorods	BTC	Aging	RT, 24 h	10.7	N/A	[161]
AgCu‐MOF microrods	BTC	Aging	RT for 24 h	27.2	0.082	[133]
Chalk‐like ZnCu‐MOF‐74	DHTP	Aging	RT for 20 h	1142	0.81	[162]
ZnCo‐ZIF (1:1) rhombic dodecahedrons	2‐MeIM	Aging	RT for 24 h	1414	0.124	[163]
Cubic CoCd‐BTC (MOF‐11)	BTC	Hydrothermal	120 °C for 48 h	584	N/A	[164]
NiFe‐MOF nanodots	2,6‐Naphthalenedicarboxylic acid dipotassium	Solvothermal	60 °C for 20 h	21.0	N/A	[120]
NiCo‐MOF 74 nanospheres	BDC	Solvothermal	100 °C for 24 h	≈730	0.05	[122]
Ni_0.25_Fe_0.75_‐MOF nanosheets	BDC	Solvothermal	Refluxed for 24 h	101	N/A	[146]
Spindle‐like Co/NH_2_‐MIL‐88B(Fe)	BDC	Solvothermal	110 °C for 18 h	31.0	0.106	[165]
Hierarchical NiCo‐BTC spheres	BTC	Solvothermal	160 °C for 10 h	90.2	0.35	[166]
Rectangular‐like NiCo‐BDC nanosheets	BDC	Solvothermal	160 °C for 10 h	9.50	0.08	[166]
Coral‐like NiCo‐MOF	Furandicarboxylic acid	Solvothermal	150 °C for 12 h	58.6	N/A	[167]
Diamond‐like FeCo‐MOF	BDC	Solvothermal	120 °C for 1.5 h	56.0	0.11	[168]
Columnar‐structured FeCu‐BDC	BDC	Solvothermal	100 °C for 12 h	14.6	0.18	[169]
CoCu‐MOF‐74 nanorods	2,5‐Dihydroxybenzene	Solvothermal	100 °C for 24 h	907	0.448	[170]
In_1_Zn_2_‐MIL‐68 microrods	BDC	Solvothermal	130 °C for 1 h	235	0.17	[171]
Ni‐MOF‐801 octahedrons	Fumaric acid	Solvothermal	130 °C for 6 h	974	0.45	[172]
Co‐MOF‐801 octahedrons	Fumaric acid	Solvothermal	130 °C for 6 h	998	0.45	[172]
8%Ti‐MOF‐808(Zr) octahedrons	BTC	Solvothermal	110 °C for 8 h	1406	0.913	[173]
NiCu‐BTC (2:8) polyhedrons	BTC	Solvothermal	120 °C for 12 h	1301	0.536	[174]
Zn‐TCPP(Pd) nanosheets	TCPP	Solvothermal	80 °C for 24 h	125.6	0.08	[151]
Tb‐Eu‐TCPP nanosheets	TCPP	Microwave	231 W for 10 min	332.1	N/A	[152]
Spindle‐like 10%Ni/Fe‐MOF	BDC	Microwave	300 W for 1 h	152	N/A	[132]
NiCo‐MOF long sheets	BDC	Microwave	200 W for 10 min	50.8	0.183	[175]
UTSA‐16 (Zn, Mg)‐(7:3) NPs	Citric acid	Microwave	300 W for 4 h	772.3	0.969	[176]
UTSA‐16 (Zn, Mn)‐(7:3) NPs	Citric acid	Microwave	300 W for 4 h	758.5	0.923	[176]
NiCo‐HHTP nanorods	HHTP	Electrochemical	5 V, 1–3 h	N/A	N/A	[134]
Crystalline ZnCu‐MOF‐74	DHTP	Mechanochemical	RT for 1.5 h	660	0.56	[177]
Co_0.5_Zn_0.5_‐ZIF NPs	2‐MeIM	Mechanochemical	RT for 1 h	1680	1.08	[178]
ZnCo‐MOF‐74 NPs	DHTP	Mechanochemical	RT for 70 min	1130	N/A	[179]
ZnMg‐MOF‐74 NPs	DHTP	Mechanochemical	RT for 70 min	1080	N/A	[179]

*RT, room temperature; UTSA, University of Texas at San Antonio.

### 3.5 Interior Nanoarchitecturing of Bimetallic MOFs

In addition to controlling the external morphology, designing and tuning the internal architecture of bimetallic MOFs is also essential. A well‐defined interior structure can provide more sites for gas adsorption by increasing the surface area and enhancing gas diffusion during the sensing process. Accordingly, numerous studies have explored bimetallic MOFs with hollow and core‒shell structures, which are discussed in detail below.

#### 3.5.1 Hollow Bimetallic MOFs

Hollow bimetallic MOFs are generally fabricated through two main methods, namely, the self‐templating method and the exterior‐templating method.^[^ ^14^ ^]^ The self‐templating method typically involves dissolution‐regrowth and ion exchange processes, resulting in hollow bimetallic MOFs. This method is straightforward and efficient since it eliminates the need for template approval. In contrast, the exterior‐templating method involves the initial creation of a core‒shell intermediate using a sacrificial template, followed by subsequent removal of the template to yield hollow bimetallic MOFs.

**Figure 8:**
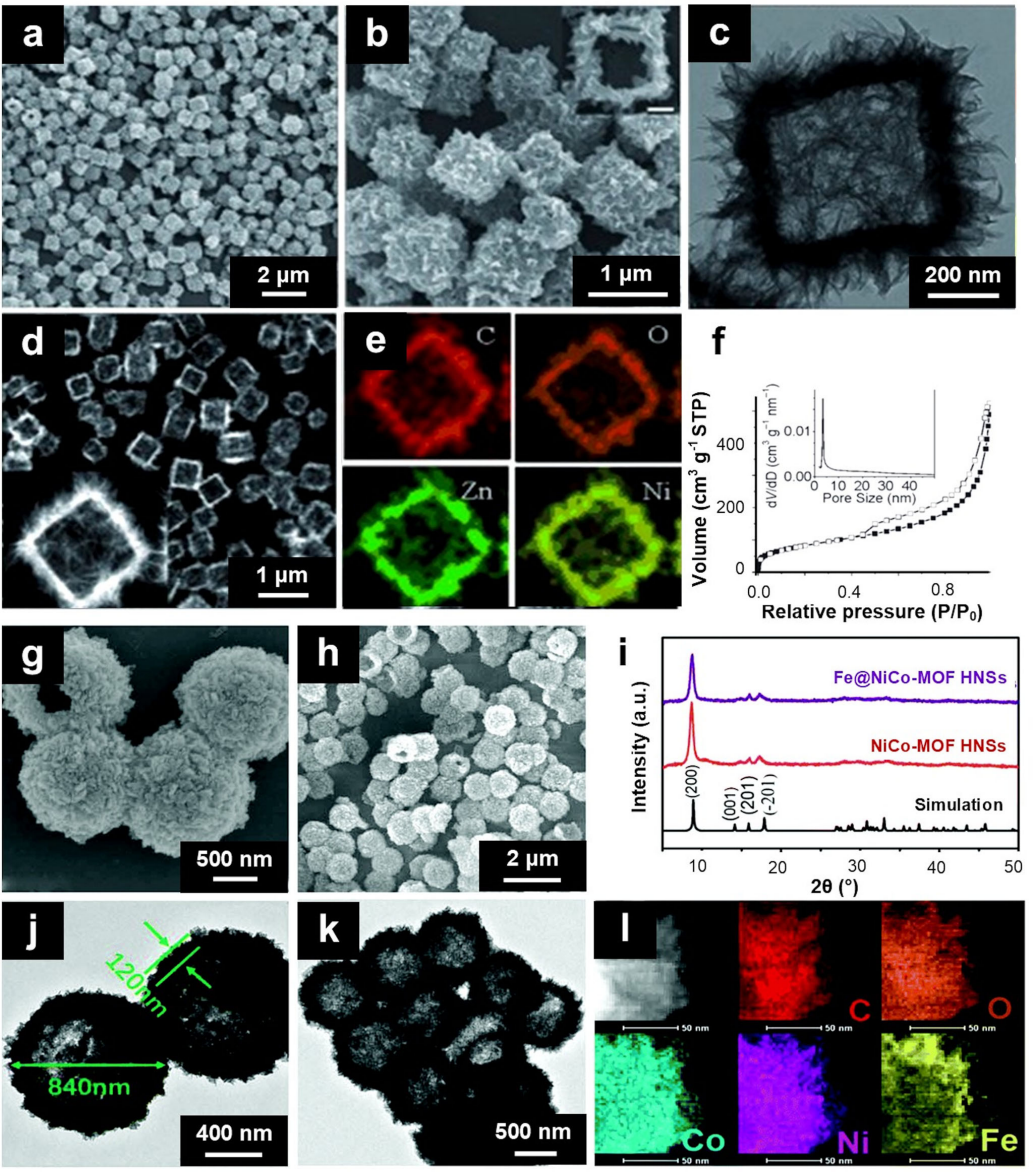
a,b) SEM images of hierarchical Zn/Ni‐MOF‐2 nanosheet‐assembled hollow nanocubes (NAHNs). c) TEM and d) high‐angle annular dark field‐scanning transmission electron microscopy (HAADF‐STEM) images of the NAHNs. e) EDS elemental mapping and f) nitrogen adsorption‒desorption isotherms of NAHNs (inset: the corresponding pore size distribution). Reproduced with permission.^[^ ^180^ ^]^ Copyright. 2014, Wiley‐VCH. g,h) SEM images of NiCo‐MOF hollow nanospheres (HNSs). i) XRD patterns of NiCo‐MOF HNSs and Fe@NiCo‐MOF HNSs. j,k) TEM images of Fe@NiCo‐MOF HNSs and l) the corresponding EDS elemental mapping. Reproduced with permission.^[^ ^181^ ^]^ Copyright 2020, Royal Society of Chemistry.

Hierarchical Zn/Ni‐MOF‐2 hollow nanocubes (**Figure** **8**a–e) were previously synthesized through a solvothermal reaction involving Ni^2+^ and Zn^2+^ ions and the BDC ligand in a mixture of N,N‐dimethylacetamide (DMAC) and ethanol.^[^ ^180^ ^]^ Initially, solid nanocubes with smooth surfaces formed, but they transformed into hollow nanocubes with sheet‐like shells with increasing Ni^2+^ content. The hollow structure originated from the faster outward diffusion of Zn^2^⁺ than from the inward diffusion of Ni^2^⁺, driven by the Kirkendall effect. These hierarchical hollow nanocubes exhibited a high SSA of 309 m^2^ g^−1^ with a pore diameter centered at 3.4 nm, as shown in Figure 8f. NiCo‐MOF hollow nanospheres (HNSs) were prepared by the direct solvothermal reaction of Ni and Co precursors with the BDC ligand in a mixture of 2‐propanol and DMAC at 150 °C for 3 h (Figure 8g,h).^[^ ^181^ ^]^ These hollow nanospheres, assembled from ultrathin nanosheets, could be made even more hollow through ion exchange with Fe^3+^ to achieve Fe‐doped NiCo‐MOF HNSs (Figure 8i–k). The Fe‐doped NiCo‐MOF HNSs exhibited uniformly distributed Ni and Co elements (Figure 8l) and a high SSA of 257 m^2^ g^−1^ with a narrow pore size distribution centered at ≈0.45 nm.

Hollow Cu‐DHTP/ZIF‐67 particles containing both Co and Cu were previously synthesized by combining etching and ligand exchange methods.^[^ ^182^ ^]^ In the first step, ZIF‐67 particles were prepared, after which Cu^2+^ and DHTP were simultaneously introduced into a methanolic solution of ZIF‐67. When only Cu^2+^ was added, Cu/ZIF‐67 dodecahedrons with slightly smoothed edges and smaller particle sizes were obtained. Conversely, the introduction of DHTP alone resulted in rough‐surface DHTP/ZIF‐67 particles, with the edges of the original ZIF‐67 dodecahedrons disappearing. The formation mechanism involved a cation exchange reaction between ZIF‐67 crystals and Cu^2+^ ions, followed by a dissolution‒regrowth process driven by the Kirkendall effect. Initially, ZIF‐67 was etched, releasing Co^2+^ ions. As dissolution proceeded faster than regeneration, the dodecahedrons were progressively degraded. Moreover, DHTP coordinated with Cu^2+^ ions, competing with the 2‐MeIM ligand. The simultaneous crystallization of metal ions and DHTP ligands occurred on the particle surface, while the ZIF‐67 crystal structure dissolved internally, ultimately forming hollow structures. The resulting hollow Cu‐DHTP/ZIF‐67 product displayed a much lower SSA (255.94 m^2^ g^−1^) than did pure ZIF‐67 (1587.45 m^2^ g^−1^), owing to the significantly enlarged pore size (from 1.98 to 8.09 nm) following Cu‐DHTP incorporation.

In another study, hollow octahedral W‐Zr‐MOF‐NH_2_ particles were obtained by subjecting Zr‐MOF‐NH_2_ to a metal‐acid assisted etching process.^[^ ^183^ ^]^ When Zr‐MOF was dispersed in an acidic solution containing NaWO_4_, WO_4_^2^⁻ anions initially diffused into the crystal lattice of Zr‐MOF‐NH₂ before elongated channels began to form. Zr‐MOF‐NH_2_ contains acid centers [Zr_6_O_4_(OH)_4_] that can react with alkaline WO_4_^2^⁻ ions via an acid‒base neutralization reaction. This interaction caused etching through hydrolysis of WO_4_^2^⁻ under acidic conditions. The porous nature of the (100) faces in this bimetallic MOF facilitated etching at the six (100) faces located at the vertices of Zr‐MOF‐NH_2_, ultimately leading to the formation of elongated channels within the W‐Zr‐MOF‐NH₂ structure. The channel width could be expanded from 20 to 220 nm by increasing the particle size. The formation of these elongated channels significantly reduced the crystallinity and changed the preferential orientation of W‐Zr‐MOF‐NH_2_ over the original Zr‐MOF‐NH_2_.

More recently, a combined metal‐ and ligand‐exchange method was developed to obtain hollow bimetallic ZnCo‐HHTP MOFs from a monometallic MOF template (ZIF‐8).^[^ ^184^ ^]^ To achieve this conversion, ZIF‐8 nanocubes were first reacted with tannic acid (TA) through an etching‐coordination process to form hollow TA‐chelated Zn network nanoboxes (TA‐Zn NBs). These TA‐Zn NBs subsequently underwent cation exchange with the newly introduced Co^2+^ ions, generating Comodified TA‐Zn NBs (TA‐ZnCo NBs). Finally, a ligand exchange reaction with HHTP, driven by its strong coordination capability, converted TA‐ZnCo NBs into hollow ZnCo‐HHTP NBs. The resulting hollow ZnCo‐HHTP NBs presented a higher SSA (76.1 m^2^ g^−1^) than did the pure Zn‐HHTP NBs (58.9 m^2^ g^−1^), highlighting the benefits of the bimetallic composition.

#### 3.5.2 Core–Shell Bimetallic MOFs

Bimetallic MOFs with a core‒shell architecture combine the benefits of two different metals into one structure. Typically, the core provides structural integrity and a large surface area, whereas the shell imparts improved chemical stability and can be chemically tailored to improve sensitivity and selectivity toward specific target gases. The synergistic integration of the core and shell components improves the sensitivity by enabling the detection of target gases even at lower concentrations. Additionally, the controllable nature of both the core and shell materials facilitates the design of highly selective sensors. These features render core‒shell bimetallic MOFs highly attractive for sensing applications. In general, three common methods can be used to synthesize core‒shell bimetallic MOFs: one‐pot (direct) synthesis, seed‐induced growth, and PSME methods.^[^ ^14^ ^]^

##### Seed‐Induced Growth

The seed‐induced growth method is a common approach for creating core‒shell bimetallic MOFs by leveraging preformed MOF seed particles as the foundation for the growth of additional MOF layers.^[^ ^77^ ^]^ The process typically begins by fabricating a monometallic MOF core or seed, which is then introduced into a solution containing the precursors for the MOF shell (usually different metal ions and organic ligands).^[^ ^14^ ^]^ Subsequently, the MOF shell nucleates and grows uniformly around the MOF core, resulting in a distinct core‒shell structure. In the seed‐induced growth process, controlling the assembly of the MOF shell onto a MOF core is critical to prevent unwanted self‐nucleation. This precise control can be achieved by carefully adjusting the reaction parameters, such as the rate of precursor addition, the concentration of precursors, and the reaction temperature and time. This method has several main advantages, including i) the production of well‐defined core‒shell structures with clear, separate phases for the core and shell components; ii) superior control over the thickness and composition of the shell; and iii) possible individual functionalization of the core and shell MOFs, thereby enabling tailored properties suitable for specific applications, including sensing.

Seed‐induced growth methods, such as epitaxial growth, provide an effective approach for creating bimetallic MOFs with core‒shell structures from isostructural MOFs. For instance, our group first reported the synthesis of ZIF‐8@ZIF‐67 core‒shell crystals via a seed‐mediated method.^[^ ^185^ ^]^ In this process, ZIF‐8 seed crystals with an average size of 500 nm were initially fabricated by simply aging a methanolic solution containing a zinc precursor and 2‐MeIM at room temperature. Subsequently, the ZIF‐67 shell was epitaxially grown on the ZIF‐8 core through coordinative interactions between Co^2+^ and 2‐MeIM on the surface of the ZIF‐8 seed crystals. The thickness of the ZIF‐67 shell could be precisely controlled by varying the Co^2+^/Zn^2+^ feeding ratio. Interestingly, the same method failed to produce ZIF‐67@ZIF‐8 core‒shell particles because of the rapid nucleation of ZIF‐8, which prevented heterogeneous nucleation on the ZIF‐67 core. The resulting ZIF‐8@ZIF‐67(0.26) crystals (obtained with a Co^2+^/Zn^2+^ ratio of 0.26) had higher SSA and pore volume (1910 m^2^ g^−1^ and 0.854 cm^3^ g^−1^) than did the pure ZIF‐8 (1727 m^2^ g^−1^ and 0.758 cm^3^ g^−1^) and ZIF‐67 (1738 m^2^ g^−1^ and 0.727 cm^3^ g^−1^) crystals, highlighting the advantages of the bimetallic composition and core‒shell architecture. Furthermore, this study revealed the possibility of tuning the SSA of core‒shell bimetallic MOFs by modifying the shell thickness, which can be controlled by varying the metal ratio. In a related study, Co‐MOF‐74@Mn‐MOF‐74 core‐shell nanorods were obtained via a similar seed‐mediated method.^[^ ^186^ ^]^ Co‐MOF‐74 seed crystals were initially prepared via a solvothermal reaction involving cobalt nitrate and the DHTP ligand in a DMF/water/ethanol mixture at 150 °C for 24 h. The Mn‐MOF‐74 shell was then grown by dispersing these seeds in a DMF solution containing MnCl_2_, PVP, and DHTP, followed by a solvothermal reaction at 150 °C for another 24 h. The thickness of the Mn‐MOF‐74 shell was adjusted from <1 to 2 µm by increasing the mass ratio of Co‐MOF‐74 to MnCl_2_·4H_2_O from 1:1 to 4:1. Furthermore, with increasing thickness of the Mn‐MOF‐74 shell, the diffraction peaks of the resulting Co‐MOF‐74@Mn‐MOF‐74 particles shifted to lower angles. This shift was attributed to the larger lattice of Mn‐MOF‐74 than that of Co‐MOF‐74. In addition, the SSA of Co‐MOF‐74@Mn‐MOF‐74 core‒shell nanorods gradually decreased with increasing shell thickness. These core‒shell samples had pore diameters ranging from 2.98 to 4.49 nm, which are larger than those of pure Co‐MOF‐74 (2.69 nm) and Mn‐MOF‐74 (3.5 nm). This expansion in pore size originated from the growth of the outer Mn‐MOF‐74 layer. Owing to their similar topology, MOF‐5@IRMOF‐3 (IRMOF = Isoreticular Metal‐Organic Framework) core‒shell crystals were successfully synthesized through a seed‐mediated (epitaxial growth) method.^[^ ^187^ ^]^ The synthesis involved immersing MOF‐5 seed particles in a precursor solution for IRMOF‐3 containing 2‐amino benzene‐1,4‐dicarboxylate (ABDC) and zinc nitrate, followed by heating at 100 °C for 15 h. The reverse approach was employed to prepare IRMOF‐3@MOF‐5 by immersing IRMOF‐3 seed crystals into the MOF‐5 precursor solution. With increasing ABDC concentration, the SSA of the resulting MOF‐5@IRMOF‐3 core‒shell products decreased in an approximately linear fashion, varying between the SSA of MOF‐5 (3170 m^2^ g^−1^) and IRMOF‐3 (2660 m^2^ g^−1^).

**Figure 9:**
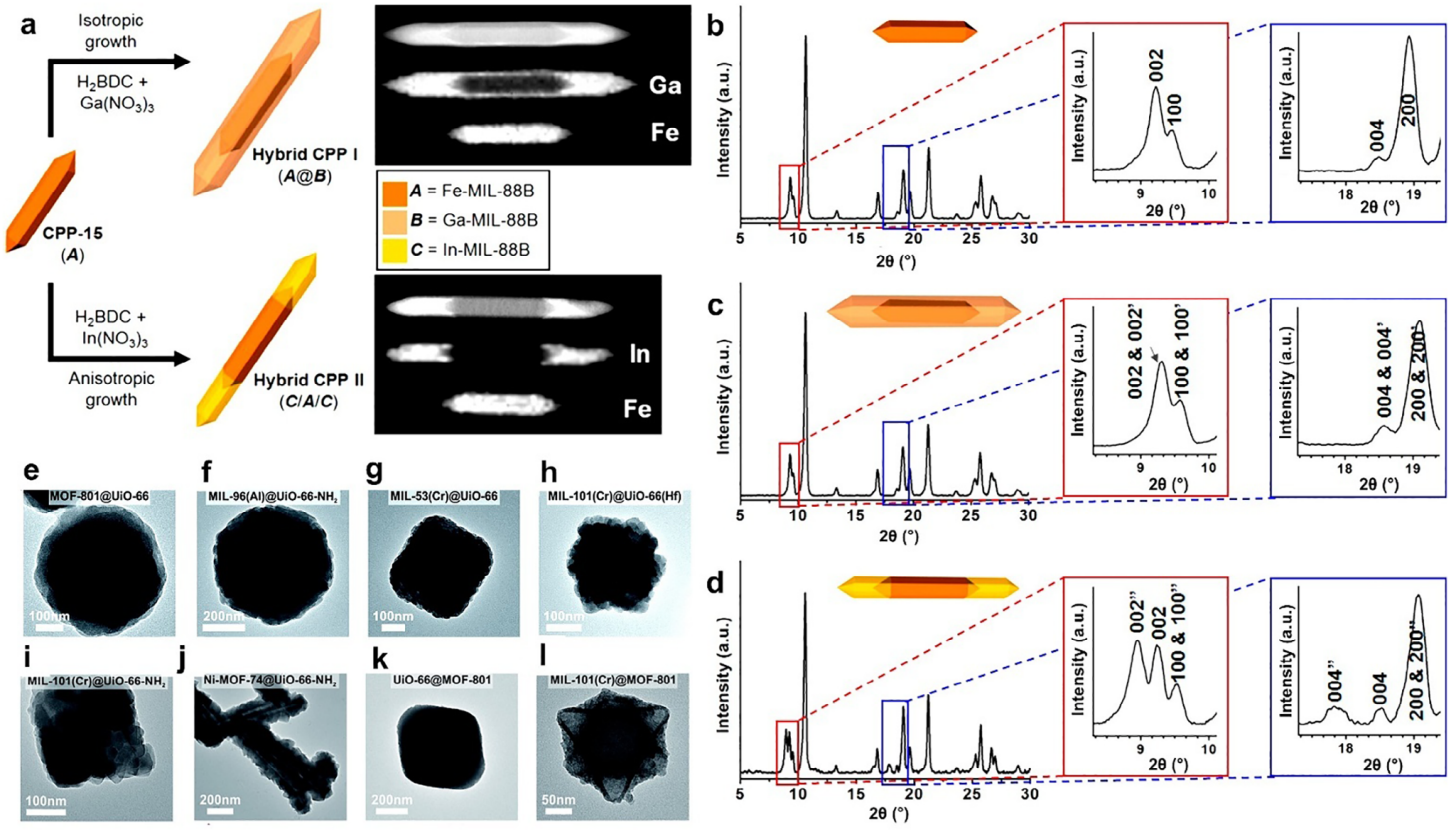
a) Schematic diagram and STEM images showing the growth of Ga‐MIL‐88B and In‐MIL‐88B shells on Fe‐MIL‐88B. XRD patterns of b) CPP‐15 (pure Fe‐MIL‐88B), c) hybrid CPP‐I (Fe‐MIL‐88B @Ga‐MIL‐88B), and c) hybrid CPP‐II (In‐MIL‐88B/Fe‐MIL‐88B/In‐MIL‐88B). Reproduced with permission.^[^ ^188^ ^]^ Copyright 2013, American Chemical Society. TEM images of e) MOF‐801@UiO‐66, f) MIL‐96(Al)@UiO‐66‐NH_2_, g) MIL‐53(Cr)@UiO‐66, h) MIL‐101(Cr)@UiO‐66(Hf), i) MIL‐101(Cr)@UiO‐66‐NH_2_, j) Ni‐MOF‐74@UiO‐66‐NH_2_, k) UiO‐66@MOF‐801, and l) MIL‐101(Cr)@MOF‐801. Reproduced with permission.^[^ ^190^ ^]^ Copyright 2019, Royal Society of Chemistry.

Differences in the lattice parameters among isostructural MOFs can significantly influence the growth behavior of the MOF shell on the MOF core during seed‐induced growth. For example, Ga‐MIL‐88B and In‐MIL‐88B shells exhibited distinct growth patterns on Fe‐MIL‐88B because of differences in ionic radii (**Figure** **9**a).^[^ ^188^ ^]^ Specifically, Ga‐MIL‐88B grows isotropically on the surface of Fe‐MIL‐88B because of the relatively similar ionic radii of Ga^3+^ (76 pm) and Fe^3+^ (78.5 pm). In contrast, In‐MIL‐88B displayed anisotropic growth along the *c*‐direction at both ends of the Fe‐MIL‐88B nanorods (Figure 9a). This difference was attributed to the larger ionic radius of In^3+^ (94 pm) relative to that of Fe^3+^ (78.5 pm), leading to a significant increase in the c‐axis parameters of In‐MIL‐88B and, consequently, its anisotropic growth. These observations were further confirmed by XRD analysis. The peaks observed in Fe‐MIL‐88B@Ga‐MIL‐88B (labeled Hybrid CPP I; CPP = coordination polymer particles) (Figure 9c) matched those of pure Fe‐MIL‐88B (Figure 9b), indicating successful isotropic growth. Conversely, the In‐MIL‐88B/Fe‐MIL‐88B/In‐MIL‐88B sample (named Hybrid CPP II) exhibited additional (002) and (004) peaks, corresponding to both Fe‐MIL‐88B and In‐MIL‐88B (Figure 9d).

Constructing core‒shell bimetallic MOFs from two MOFs with different crystallographic parameters via epitaxial growth is challenging but desirable for enhancing their functionality. In such cases, seed‐induced growth methods assisted by capping agents can be used to produce bimetallic MOFs with core‒shell structures. For example, UiO‐66@ZIF‐8 core‒shell particles were successfully prepared using the surfactant cetyltrimethylammonium bromide (CTAB), despite the differing crystal structures and topologies of these MOFs.^[^ ^189^ ^]^ In this process, UiO‐66 microcrystals were first prepared as seed particles and then mixed with CTAB and ZIF‐8 precursors in water. Ultrasonication facilitated the formation of small, uniform ZIF‐8 nuclei, which adhered to the UiO‐66 surface through CTAB layers and gradually grew into a conformal shell, yielding UiO‐66@ZIF‐8 core‒shell structures. Replacing CTAB with other ionic surfactants, such as cetylpyridinium bromide (CPB), tetradecyltrimethylammonium bromide (TTAB), and sodium dodecyl sulfate (SDS), generated fractured ZIF‐8 shells consisting of small, irregular nanocrystals (NCs). Moreover, when PVP was used instead of ionic surfactants, no ZIF‐8 overgrowth on UiO‐66 was observed because of the lack of interactions between PVP and the MOFs in water. In contrast, conducting the PVP‐mediated reaction in methanol led to the embedding of several UiO‐66 NCs within the ZIF‐8 particles because of stronger interactions in this solvent. The final UiO‐66@ZIF‐8 core‒shell particles were predominantly micropores, leading to a large SSA of 1240 m^2^ g^−1^.

Wang et al. demonstrated that the use of surfactants or capping agents is not always necessary for creating core‒shell bimetallic MOFs from two MOFs with different topologies and lattice parameters.^[^ ^190^ ^]^ Instead, they successfully employed a nucleation kinetic‐guided growth strategy to grow a UiO‐66 shell on a MIL‐101(Cr) core. At lower concentrations of ZrCl_4_ and BDC (25 and 50 mm) and shorter reaction times, the surface coverage of the MIL‐101(Cr) core by the UiO‐66 shell was incomplete. By optimizing the concentrations of ZrCl_4_ and BDC to 100 mM and increasing the reaction time to 1 h, uniform MIL‐101(Cr)@UiO‐66 core‒shell particles were obtained. This nucleation kinetic‐guided growth strategy was successfully generalized for the preparation of other core‐shell MOFs, including MOF‐801@UiO‐66, MIL‐96(Al)@UiO‐66‐NH_2_, MIL‐53(Cr)@UiO‐66, MIL‐101(Cr)@UiO‐66(Hf), MIL‐101(Cr)@UiO‐66‐NH_2_, Ni‐MOF‐74@UiO‐66‐NH_2_, UiO‐66@MOF‐801, and MIL‐101(Cr)@MOF‐801 (Figure 9e–l). However, the growth of Zr‐based MOFs, such as UiO‐66, on acid‒labile cores (e.g., ZIF‐8) remains challenging since the acidic precursors (Zr^4+^ and BDC) required to synthesize UiO‐66 can induce dissolution of the ZIF‐8 core. To overcome this issue, they employed a ZIF‐8/UiO‐66‐NH_2_ growth solution ratio of 1:1 (precursor concentration = 100 mm) and a short reaction time of 2 h to form an initial amorphous zirconium‐ligand complex layer on the ZIF‐8 surface. Afterward, the ZIF‐8 core underwent controlled digestion, followed by repetition of the growth process using fresh growth solution, ultimately yielding the desired ZIF‐8@UiO‐66‐NH_2_ core‒shell structure. Importantly, the initially formed amorphous zirconium‐ligand complex passivated the ZIF‐8 surface, thus limiting the diffusion of acidic species into ZIF‐8 and effectively preventing its dissolution.

In summary, nucleation and dissolution kinetics are critical factors influencing the formation of core‒shell bimetallic MOFs from two MOFs with distinct topologies. Nucleation kinetics affect the size of the MOF shell, which in turn influences the coverage and uniformity of the shell on the core surface. On the other hand, dissolution kinetics regulate the dissolution rate of the MOF core during shell growth, impacting the overall structural integrity of the core‒shell product. Although the seed‐induced method has been successfully applied for creating well‐defined core‒shell bimetallic MOFs from MOFs with similar and dissimilar lattice parameters, several limitations remain. First, careful control of the growth conditions is required to ensure uniform shell growth around the MOF core, making the synthesis process complex and time‐consuming. Second, poorly controlled growth conditions may result in unwanted homogeneous nucleation of the secondary MOF, resulting in the formation of separate particles rather than a uniform shell. Finally, precise control over shell thickness and morphology remains challenging owing to the inherently dynamic nature of secondary MOF nucleation and growth processes.

##### One‐Pot (Direct) Synthesis

**Figure 10:**
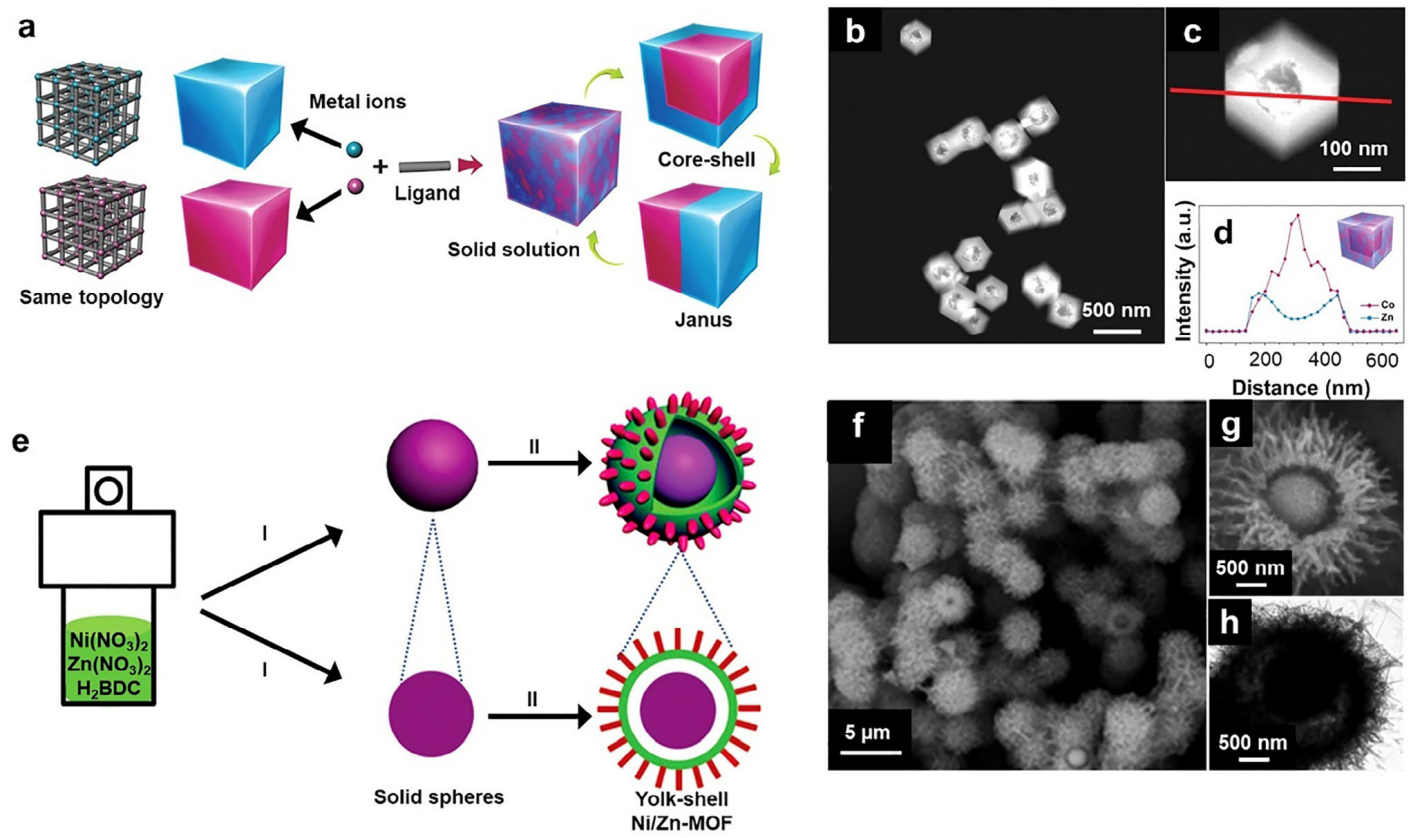
a) Schematic diagram showing the synthesis of hybrid MOF nanoarchitectures. b,c) HAADF‐STEM images of the ZIF‐67@ZIF‐8/ZIF‐67 core‒shell NPs and d) the related EDS profile along the red line shown in (c) (inset: structural model of the core‒shell nanoparticle). Reproduced with permission.^[^ ^192^ ^]^ Copyright 2017, Wiley‐VCH. e) Schematic illustration depicting the formation of yolk‐shell Ni/Zn‐MOF microspheres and their transformation into double‐shelled NiO/ZnO hollow spheres. f,g) SEM and h) TEM images of the yolk‐shell Ni/Zn‐MOF microspheres. Reproduced with permission.^[^ ^193^ ^]^ Copyright 2016, Royal Society of Chemistry.

One‐pot (direct) synthesis offers a simple and straightforward approach for preparing core‒shell bimetallic MOFs, as it eliminates the need for separation and purification of intermediates.^[^ ^14^, ^191^ ^]^ In this approach, precursors for both the MOF core and MOF shell are typically combined in a single solution. However, careful control of the nucleation and growth kinetics of the two MOFs within the solution is crucial to ensure exclusive growth of the MOF shell on the MOF core surface and to prevent undesired self‐nucleation. To achieve this, proper regulation of the reaction parameters (e.g., ratio of metal precursors, reaction temperature and time, solvents, and modulators) is essential to balance the rates of self‐nucleation and growth of both the MOF core and MOF shell, thus enabling the formation of a well‐defined core‒shell structure. For example, ZIF‐67@ZIF‐8/67 core‒shell particles were successfully fabricated by precisely controlling the metal addition sequence and growth kinetics (**Figure** **10**a).^[^ ^192^ ^]^ Specifically, the Co precursor solution was first added to the 2‐MeIM solution, followed by the addition of a Zn precursor solution after 5 minutes.^[^ ^192^ ^]^ The resulting core‒shell particles featured a Co‐only core ≈160 nm in diameter, surrounded by an ≈95 nm thick shell containing both Zn and Co (Figure 10b–d). Conversely, reversing the addition sequence by introducing the Zn precursor first led to the formation of irregular NPs. Kinetic studies further revealed the dominant role of Co in the formation of these bimetallic ZIF‐based core‒shell particles because of its faster nucleation and growth kinetics. This study highlights the critical importance of the order of metal addition in the one‐pot synthesis of core‒shell bimetallic MOFs.

Ni/Zn‐MOF yolk‒shell microspheres were synthesized through a direct solvothermal reaction of Ni and Zn salts with the BDC ligand in a DMF/ethylene glycol mixture at 150 °C for 6 h (Figure 10e).^[^ ^193^ ^]^ During the reaction, solid Ni/Zn‐MOF spheres formed initially. With increasing reaction time, the nanorods gradually self‐assembled on these solid spheres, generating Ni/Zn‐MOF yolk‒shell microspheres (Figure 10f–h). Cr/V‐MIL‐53@Cr‐MIL‐53 core‐shell particles were previously obtained via a one‐pot microwave‐assisted reaction by heating a solution containing Cr and V precursors and the BDC ligand to 200 °C at 200 W.^[^ ^194^ ^]^ In contrast, employing a solvothermal reaction with the same precursor mixture resulted in homogeneous bimetallic Cr/V‐MIL‐53 without a core‐shell structure, highlighting the profound effect of the synthesis method on the metal distribution in bimetallic MOFs. Additionally, a one‐pot strategy involving ion modulation (with Fe^3+^ as the modulator) has been developed to synthesize PBA@PBA core‐shell particles (PBA = Prussian blue analog).^[^ ^195^ ^]^ Specifically, Mn_3_[Fe(CN)_6_]_2_@Mn_2_[Fe(CN)_4_]_3_ core‒shell NCs were fabricated by mixing Fe and Mn precursors (in an ethanol/water/PVP mixture, with [Fe^3+^]/([Fe^3+^] + [Mn^2+^]) = 0.2) with potassium hexacyanoferrate(III) (K_3_Fe(CN)_6_), followed by stirring at ambient temperature for 1 h. Mn^II^–NC–Fe^III^ (cubic phase) and Mn^III^–NC–Fe^II^ (tetragonal phase) exhibited similar crystallographic parameters, facilitating the formation of a core‒shell structure.

**Figure 11:**
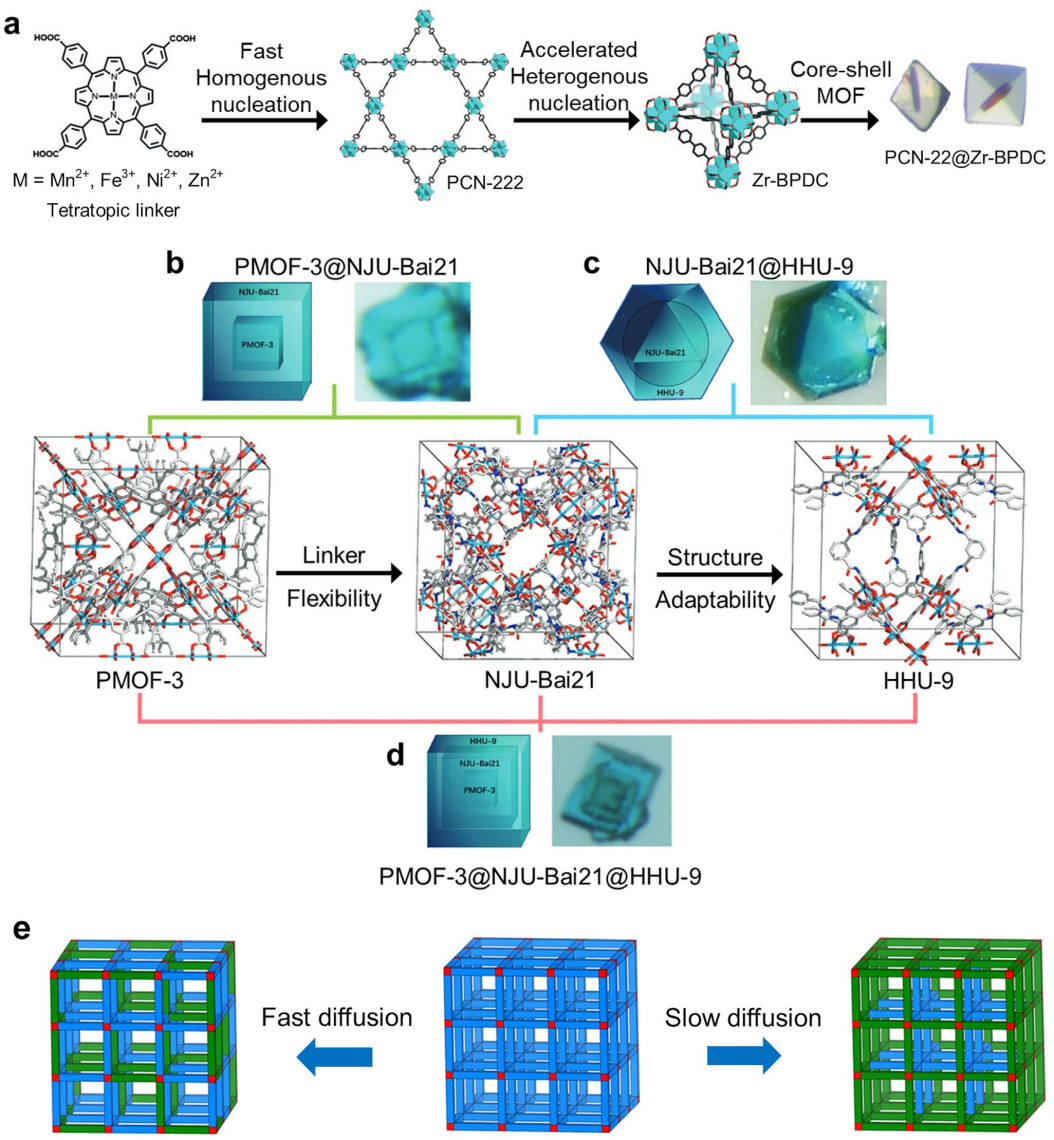
a) Kinetically guided synthesis of PCN‐222@Zr‐BPDC core‒shell particles, demonstrating precise control of core‒shell formation through selective kinetic conditions that allow for targeted layering and structural assembly. Reproduced with permission.^[^ ^196^ ^]^ Copyright 2018, Wiley‐VCH. b) Control of nucleation kinetics through ligand flexibility and structural adaptability: b) ligand flexibility promotes PMOF‐3@NJU‐Bai21 core‐shell formation; c) structural adaptability facilitates NJU‐Bai21@HHU‐9 core‐shell formation; and d) a one‐pot synthesis sequence yields triple‐layered PMOF‐3@NJU‐Bai21@HHU‐9. Reproduced with permission.^[^ ^197^ ^]^ Copyright 2024, Wiley‐VCH. e) Two different ligand incorporation models: (left) a uniform distribution resulting from fast diffusion relative to the exchange process, leading to a homogeneous structure; and (right) a core‒shell distribution resulting from slow diffusion relative to the exchange process, producing a distinct layered architecture. Reproduced with permission.^[^ ^203^ ^]^ Copyright 2017, American Chemical Society.

Unlike the epitaxial growth method, which typically requires good lattice matching between the MOF core and shell, the one‐pot synthesis approach can bypass this stringent requirement and enable the creation of core‒shell bimetallic MOFs from two MOFs with poor lattice matching. For example, Zr‐based PCN‐222@Zr‐BPDC (PCN = porous coordination network) core‒shell particles with mismatched lattices were successfully fabricated via a one‐pot solvothermal reaction of ZrCl_4_ with TCPP and BPDC ligands by carefully controlling the nucleation kinetics.^[^ ^196^ ^]^ Due to the weaker coordination between BPDC and Zr^4+^ compared with TCPP, PCN‐222 nucleated first to form the core, followed by subsequent nucleation and growth of the Zr‐BPDC shell around the PCN‐222 core (**Figure** **11**a). Interestingly, in the absence of the TCPP ligand, the formation of Zr‐BPDC was significantly slower, emphasizing the critical role of the PCN‐222 core in promoting heterogeneous nucleation of Zr‐BPDC. The resulting PCN‐222@Zr‐BPDC core‒shell particles exhibited abundant mesopores originating from the mesoporous PCN‐222 core. The same strategy was successfully applied to create other core‒shell systems, such as PCN‐222@Zr‐NDC and PCN‐222@Zr‐AZDC (AZDC = azobenzene dicarboxylate), which contain ligands that are either shorter or longer than Zr‐BPDC. Additionally, the versatility of this method was further demonstrated through the synthesis of core‐shell bimetallic MOFs using mixed‐ligand and 2D MOFs (e.g., PCN‐134@Zr‐BTB; BTB = 1,3,5‐benzenetribenzoate), MOFs containing two ligands with identical connectivity (e.g., PCN‐222@NU‐1000; NU = Northwestern University), and MOFs with mismatched lattices and different metal nodes (e.g., La‐TCPP@La‐BPDC). Overall, this study highlighted the flexibility and broad applicability of the one‐pot synthesis method for constructing core‒shell bimetallic MOFs from MOFs with diverse lattice parameters, ligand types and lengths, and metal nodes.

In a recent study, Lu et al. proposed structural incompatibility (rather than lattice mismatch) as the key factor for successfully creating core‐shell bimetallic MOFs (MOF@MOF) via a one‐pot synthesis method.^[^ ^197^ ^]^ This concept was demonstrated through the synthesis of PMOF‐3@HHU‐9 (PMOF = Porphyrin Metal‐Organic Framework; HHU = Heinrich Heine University Düsseldorf) core‒shell particles via a one‐pot approach. Despite having isoreticular crystal lattices, these MOFs exhibited incompatible catenation due to the ligand flexibility in HHU‐9. In contrast, two MOFs with matched crystal lattices and isoreticular networks, NJU‐Bai20 (NJU‐Bai = Nanjing University – Bai’s group) and NJU‐Bai21, formed a multivariate MOF with randomly distributed, mixed ligands instead of a defined core‒shell structure. The authors further argued that ligand flexibility and structural flexibility, rather than ligand connectivity, primarily determine the nucleation kinetics required to achieve an isotropic core‒shell architecture. This was supported by the successful formation of PMOF‐3@NJU‐Bai21, which resulted from the slower nucleation kinetics of NJU‐Bai21 relative to those of PMOF‐3, despite NJU‐Bai21 having a more flexible ligand (Figure 11b,c). Moreover, structurally flexible MOFs derived from flexible ligands presented the slowest nucleation rates and greatest adaptability. For example, NJU‐Bai21@HHU‐9 formed successfully because the greater structural flexibility of NJU‐Bai21 slowed the nucleation of HHU‐9. Overall, the nucleation kinetics followed the order HHU‐9 < NJU‐Bai21 < PMOF‐3. Consequently, in a one‐pot synthesis, any combination of these three MOFs could produce a core‒shell structure, with the slower‐nucleating MOF forming the shell around a faster‐nucleating core. Leveraging the distinct flexibility of these MOFs, the multilayered PMOF‐3@NJU‐Bai21@HHU‐9 structure (Figure 11d) was successfully synthesized, marking the first creation of a multilayered core‒shell MOF through a one‐pot approach.

##### Post‐Synthetic Exchange (PSE)

In addition to the seed‐induced approach, core‒shell bimetallic MOFs composed of two MOFs with distinct topologies can also be achieved through postsynthetic exchange (PSE). The PSE approach typically includes PSME and postsynthetic ligand exchange (PSLE). To fabricate core‒shell bimetallic MOFs via PSME, the MOF core is first synthesized and subsequently immersed in a solution containing a secondary metal precursor.^[^ ^106^ ^]^ This step allows the selective replacement (also known as “selective transmetalation”) of metal ions at the surface region of the MOF core with secondary metal ions, while the metal ions within the inner core remain intact.^[^ ^14^, ^77^ ^]^ The extent of metal exchange in a PSME can be precisely tuned by controlling the reaction parameters, such as the reaction temperature and time and the concentration of the secondary metal solution.^[^ ^114^, ^198^ ^]^ Typically, shorter reaction times are preferable to limit exchange to the surface (shell), whereas longer reaction times may lead to deeper penetration, causing bulk metal exchange. Furthermore, optimizing the concentration of the secondary metal solution is essential to achieve selective metal exchange exclusively at the MOF surface, avoiding unwanted bulk metal exchange.

Currently, there are very few reports on the development of core‒shell bimetallic MOFs by PSME. Song and co‐workers demonstrated the first selective transmetalation of M_6_(BTB)_4_(BP)_3_ (where M = Zn(II) (**1**), Co(II) (**2**), Cu(II) (**3**), Ni(II) (**4**), and BP = 4,4′‐dipyridyl) to generate core‒shell bimetallic MOFs.^[^ ^198^ ^]^ Several well‐defined core‒shell bimetallic MOFs, named 1@2, 1@3, and 1@4, were successfully synthesized by employing the thermodynamically more stable crystals 2–4 as MOF cores and the less stable crystal 1 as the MOF shell. Specifically, by immersing the 2–4 core crystals in a DMF solution containing the Zn precursor, H_3_BTB, and BP at ambient temperature, shell crystal 1 epitaxially grew on the surfaces of these cores over three days. In a separate study, Song and co‐workers explored the selective transmetalation of two different Zn‐based MOFs with varying ligand lengths, Zn‐HKUST‐1 and Zn‐PMOF‐2 (Zn_24_L_8_(H_2_O)_12_, where L represents 1,3,5‐tris(3,5‐dicarboxylphenylethynyl)benzene), by soaking them in Cu(II)‐containing methanol solutions.^[^ ^114^ ^]^ The results revealed differences in the transmetalation rates, with Zn‐PMOF‐2 undergoing metal exchange significantly faster than Zn‐HKUST‐1 over three months, which was attributed to the greater structural flexibility of Zn‐PMOF‐2. In both cases, metal ion exchange was confined primarily to the outer surface, resulting in the formation of core‒shell bimetallic MOFs.

In contrast to PSME, the PSLE method is more widely used for fabricating core‐shell bimetallic MOFs. In PSLE, a MOF core crystal is typically immersed in a solution containing a different ligand. This leads to selective exchange between the original organic ligand of the MOF core and the newly introduced ligand at the outer surface, resulting in the formation of a core‒shell structure.^[^ ^106^ ^]^ During the PSLE process, various parameters, such as temperature, time, solvent, and ligand concentration, must be carefully controlled. To achieve complete substitution of the original ligand, an excess of the new ligand is usually needed, with an exchange‐to‐original ligand ratio exceeding 4:1.^[^ ^199^, ^200^ ^]^ Conversely, for partial substitution, a stoichiometric ratio of 1:1 or lower is typically used.^[^ ^201^, ^202^ ^]^ A crucial consideration during PSLE is ensuring that the ligand exchange rate at the edges of the parent (original) MOF crystal significantly exceeds the rate of ligand diffusion into the pores to achieve core‒shell bimetallic MOFs (Figure 11e). For example, Boissonnault et al. successfully fabricated MOF‐5@UiO‐66 core‐shell crystals by soaking MOF‐5 crystals in a 0.01 M tetrahydrofuran (THF) solution containing a 1:1 mixture of H_2_BDC‐*d*_4_/BDC (H_2_BDC‐*d*_4 =_ benzene‐2,3,5,6‐*d*_4_‐1,4‐dicarboxylic acid) and BDC at room temperature for 18 h.^[^ ^203^ ^]^ These core‒shell crystals formed due to the much slower diffusion of the BDC‐*d*_4_ ligand into the pores of MOF‐5 than its surface exchange rate with BDC. This PSLE approach was also successfully extended to another Zr‐based MOF, UMCM‐8 (UMCM = University of Michigan Crystalline Material), demonstrating the generality of the method. However, a notable challenge with this approach is that the PSLE rate strongly depends on the nature of the metal clusters, complicating the precise determination of the resulting microstructure. Although the PSLE strategy has previously been successfully employed to fabricate core‐shell MOFs from two MOFs containing the same metal center, such as MOF‐5@ZIF‐8 and ZIF‐67@Co‐MOF‐74, it remains difficult to apply this method to core‐shell bimetallic MOFs containing two different metal centers.^[^ ^204^, ^205^ ^]^

The advantages and disadvantages of different strategies for fabricating core‒shell bimetallic MOFs are summarized in **Table** **2**.

**Table 2:** Advantages and disadvantages of various strategies used to fabricate core‒shell bimetallic MOFs.

Strategy	Advantages	Disadvantages
One‐pot synthesis	Simple, scalable, and cost‐effective. Usually, good distribution of metals throughout the framework.	Difficult to control the interactions between the metal ions. Limited control over the core‐shell architecture. Usually, requires good lattice matching.
Seed‐induced growth	Formation of well‐defined core‐shell structure. Better control over the thickness, composition, and functionality of the shell. Possibility to produce a wide range of core‐shell structures using different core templates and growth conditions.	Complex and multistep. Time‐consuming. Secondary nucleation is possible if growth conditions are not well‐controlled.
PSE	Enables selective exchange of metal ions at the surface of MOF core only to prevent bulk exchange. Addition of functionality or reactivity to the MOF core without requiring complete reconstruction.	Limited to exchangeable metals: Inconsistent metal distribution Possible structural destruction due to exchange conditions

## 4 Quartz Crystal Microbalance (QCM) Gas Sensors

The QCM is an advanced analytical instrument used for the precise measurement of mass changes.^[^ ^206^, ^207^, ^208^, ^209^ ^]^ This technique operates by detecting shifts in the oscillation frequency of a quartz crystal, which occur due to mass variations on the surface of the crystal.^[^ ^209^, ^210^, ^211^ ^]^ The high sensitivity of the QCM enables the detection of very small mass changes, ranging from micrograms to nanograms, along with rapid response times.^[^ ^211^, ^212^, ^213^ ^]^ Additionally, the QCM is notable for its ease of fabrication, potential for miniaturization, and suitability for measurements across a broad temperature range.^[^ ^214^, ^215^ ^]^ These features make it particularly valuable in surface adsorption studies, gas sensing, and monitoring of chemical reactions and biomolecular interactions.

To date, various types of materials, including metal oxides, organic polymers, carbon, and, more recently, MOFs, have been investigated as sensing layers in QCM gas sensors.^[^ ^30^, ^43^, ^44^, ^216^ ^]^ Each of these materials possesses distinct properties suitable for detecting specific gases. For example, graphitic carbon nitride‐coated films, polyvinyl acetate (PVA) nanofibers, and Ni nanofibers have been used to detect hydrogen, trimethylamine (TMA), and methanol, respectively.^[^ ^41^, ^217^, ^218^ ^]^ However, these conventional sensing materials often lack sufficient porosity and adsorption sites to capture target gas molecules effectively. To increase the sensitivity and selectivity of QCM gas sensors, nanoporous materials, such as functionalized mesoporous silica, porous metal oxides, and MOFs, have been introduced.^[^ ^30^, ^219^ ^]^ These materials offer increased surface area and porosity, resulting in a greater number of adsorption sites and improved diffusion of gas molecules through the pores, thereby enhancing the performance of the resulting QCM gas sensors.

### 4.1 Working Principle of QCM Gas Sensors

**Figure 12:**
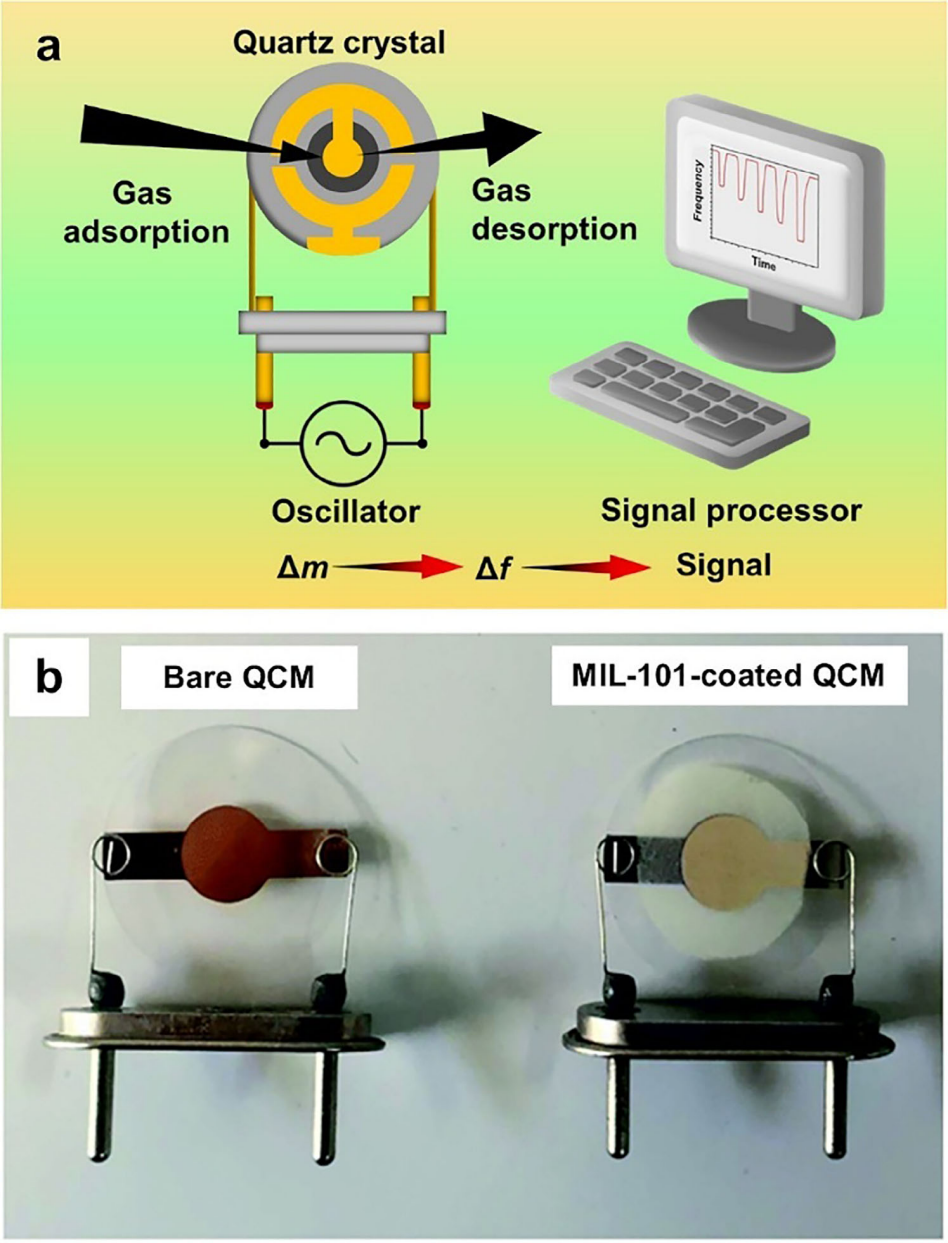
a) Schematic diagram depicting the experimental apparatus and working principle of the QCM. b) Digital photograph showing bare QCM and MIL‐10(Cr)‐coated QCM sensors. Reproduced with permission.^[^ ^243^ ^]^ Copyright 2019, Royal Society of Chemistry.

The operating principle of the QCM is based on the piezoelectric property of quartz crystals.^[^ ^206^ ^]^ In 1880, Jacques and Pierre Curie discovered that various crystals, such as quartz, tourmaline, and Rochelle salt (NaKC_4_H_4_O_6_·4H_2_O), generated electrical potentials proportional to mechanically induced surface stresses, a phenomenon termed the “piezoelectric effect” (derived from the Greek word *piezein*, meaning “to press”).^[^ ^220^ ^]^ Notably, this process is reversible, as the application of an alternating voltage to a quartz crystal causes it to oscillate at its fundamental frequency, which is determined by the thickness and mass of the crystal. In the QCM, the frequency shift of a quartz crystal depends not only on the intrinsic properties of the crystal (such as size, cut, density, and shear modulus) but also on the layers or phases attached to its surface.^[^ ^221^ ^]^ For analytical sensing applications, the proportional relationship between the crystal's resonant frequency and mass is paramount, forming the basis of piezoelectric sensors for detecting mass changes.^[^ ^45^ ^]^ Typically, a gold‐coated quartz crystal plate is used, and it is further modified with a thin sensing layer (e.g., a MOF layer) that selectively adsorbs specific gas molecules (**Figure** **12**a,b).^[^ ^222^ ^]^ When a gaseous analyte is introduced into the sensing chamber, it adsorbs onto the surface of the QCM sensor by either physisorption or chemisorption. This adsorption increases the mass of the crystal, resulting in a decrease in its resonant frequency. The mass sensitivity of the QCM strongly depends on the crystal thickness, which directly affects its resonant frequency.^[^ ^30^ ^]^ To regenerate the sensor, high‐purity nitrogen is introduced into the chamber to remove (desorb) the adsorbed gaseous analyte, restoring the QCM frequency to its initial baseline value.

In the QCM, the relationship between the frequency change (Δ*f*) of the oscillating quartz crystal and the corresponding mass change (∆*m*) on its surface is described by Sauerbrey's equation (Equations 1 and 2):^[^ ^10^, ^45^ ^]^

(1) $$\Delta f=\left(\frac{2Nf_{o}^{2}}{\sqrt{\rho_{q}\mu_{q}}}\right)\;\frac{\Delta m}{A}$$

(2) $$\Delta f=f_{1}-f_{0}$$

where *N* is the harmonic overtone, *f*_0_ (Hz) is the fundamental resonance frequency of the QCM, *ρ_q_* is the density of the quartz crystal (2.649 g cm^−3^), *µ_q_* is the shear modulus (2.947 × 10^11^ g cm^−1^ s^−2^) and *A* is the surface area of the gold electrode. According to Sauerbrey's equation (Equation 1), Δ*f* is proportional to the mass of the analyte adsorbed onto the sensing layer coated on the QCM electrode. The concentration of the analyte is one of the important factors determining the Δ*f* value. For liquid analytes, the concentration can be calculated via the following formula:^[^ ^10^, ^13^ ^]^

(3) $$C_{ppm}=\left(\frac{22.4w\rho TV_{s}}{273MV}\right)\times 10^{3}$$

here, *C*_ppm_ is the concentration of the analyte in ppm, *w* is the mass concentration of the liquid analyte (wt.%), *ρ* is the density of the injected analyte (g mL^−1^), *T* is the operating temperature (K), *M* is the molecular weight of the injected analyte (g moL^−1^), *V*_s_ is the volume of the injected analyte (µL), and *V* is the volume of the working glass vessel (L). Generally, a higher concentration of the injected analyte results in a greater mass change on the quartz crystal surface, leading to a more pronounced decrease in Δ*f*. Aside from sensitivity (reflected by Δ*f*), achieving high selectivity toward the target gas is also essential for practical applications. Selectivity is typically evaluated via the following equation:^[^ ^10^, ^13^ ^]^

(4) $$\sigma =\left(\frac{\Delta f_{i}^{gas}}{\sum_{i=1}^{N}\Delta f_{i}^{gas}}\right)\times 100\%$$

where Δ*f_i_^gas^* corresponds to the frequency change associated with the target analyte. Similarly, long‐term stability is crucial for ensuring the accuracy, reliability, and cost effectiveness of QCM gas sensors. Stability is typically assessed by measuring Δ*f* values in response to the target analyte over extended periods (weeks to months).

Another important set of parameters in QCM gas sensors is the response and recovery times. The response time is defined as the time required for the sensor to reach 90% of its final Δ*f* after exposure to a specific concentration of the analyte. In comparison, recovery time is defined as the time needed for the sensor's frequency to return to within 90% of its initial baseline value after the analyte is removed. Both response and recovery times depend primarily on the rate at which the analyte molecules adsorb onto and desorb from the QCM sensing surface. For MOF‐based QCM gas sensors, the sensing performance is typically governed by several key factors, including the following:

- Surface area and pore size: MOFs with higher surface areas can offer more adsorption sites for gas molecules, leading to higher sensitivity due to greater uptake of the gaseous analyte. Ideally, the pore size of the MOFs should be larger than the dimensions of the target gas molecules to enable efficient diffusion into and out of the sensing layer, resulting in shorter response and recovery times.
- Particle size and morphology: Larger MOF particles typically lead to longer diffusion pathways for gas molecules, which results in slower response and recovery times. Conversely, low‐dimensional MOF nanostructures, such as 1D nanorods or 2D nanosheets, provide significantly shorter gas diffusion paths, facilitating rapid adsorption and desorption. This enhances the response and recovery performance compared with those of bulk MOF particles. Additionally, in 2D MOF nanosheets, the layer thickness can be precisely controlled to tune the selectivity toward specific analytes.
- Adsorption and desorption kinetics: Stronger interactions between the MOF and target gas molecules (e.g., hydrogen bonding, π–π interactions, or metal‒ligand coordination) generally lead to faster response times but slower recovery times owing to prolonged desorption processes from the MOF surface. Additionally, larger gas molecules require more time to diffuse through MOF pores, contributing to longer response and recovery times. Owing to the slower desorption of the gas from the MOF surface. Density functional theory (DFT) calculations can be employed to estimate the adsorption energies of specific gas molecules interacting with the MOF surface.
- Operating temperature and humidity: Higher operating temperatures increase both the adsorption and desorption rates of gas molecules, resulting in shorter response and recovery times. Conversely, increased humidity introduces competition between water vapor and the target gas molecules for adsorption sites on the MOF surface, which decreases both the sensitivity and response time.

### 4.2 Sensing Mechanisms of MOF‐Based Gas Sensors

In MOF‐based QCM gas sensors, the sensing mechanism relies primarily on the interaction between gas molecules and the MOF sensing layer. This interaction typically involves physical or chemical adsorption of analyte molecules onto the MOF surface, resulting in a measurable mass increase and corresponding frequency shift. Common types of interactions include van der Waals forces, π–π interactions, electrostatic interactions, and hydrogen bonding.^[^ ^30^ ^]^ The choice of metal nodes and organic ligands in MOFs can affect the strength and nature of these interactions, increasing the sensitivity of detection. Additionally, structural factors, such as crystallinity, morphology, and surface area, can strongly influence sensitivity. They can be controlled by modifying the synthesis parameters (e.g., reaction temperature, reaction time, and metal‐to‐ligand ratio). Crystalline MOFs generally feature stable structures, ordered pores, and excellent pore connectivity, all of which increase their sensitivity, selectivity, and stability in gas‐sensing applications. Moreover, low‐dimensional MOFs (e.g., MOF nanosheets or nanorods) generally outperform bulk MOFs because of their higher surface areas, facilitating improved gas adsorption and more effective interactions with gas molecules. Selectivity can be optimized by fine‐tuning the choice of organic ligand, pore size, and surface chemistry of MOFs, allowing precise targeting of specific analytes. Additionally, other factors, such as intermolecular forces (e.g., hydrogen bonding and dipole‒dipole forces between ligands and gas molecules) and electrostatic interactions, can influence selectivity.^[^ ^18^ ^]^

In recent years, several theoretical investigations have investigated the adsorption behaviors of different gases onto MOFs (including bimetallic MOFs). For example, Li and co‐workers examined the adsorption of different gases, including SO_2_, H_2_S, SOF_2_, and CF_4,_ on Ni‐doped ZIF‐8 clusters.^[^ ^223^ ^]^ Their findings revealed that the adsorption strength of these clusters followed the sequence SO_2_ > H_2_S > SOF_2_ > CF_4_. Furthermore, adsorption energy calculations indicated that SO_2_ adsorption likely involved chemical bonding between the Ni atom and the oxygen atom of SO_2_. In contrast, H_2_S adsorption was facilitated by chemical bonding between the Ni atom and a H atom of H_2_S. Lei et al. previously studied the adsorption of CF_4_, CS_2_, H_2_S, SF_6_, SO_2_, SOF_2_ and SO_2_F_2_ on Mg‐MOF‐74.^[^ ^224^ ^]^ Their results revealed adsorption energy values in the following order: H_2_S (−0.474 eV) > SO_2_ (−0.425 eV) > SOF_2_ (−0.418 eV) > SO_2_F_2_ (−0.363 eV) > CS_2_ (−0.296 eV) > SF_6_ (−0.208 eV) > CF_4_ (−0.179 eV), indicating a high affinity for H_2_S. Furthermore, charge distribution analysis revealed that Mg‐MOF‐74 exhibited electron‐withdrawing properties during adsorption, whereas the gases exhibited electron‐donating properties.

The sensing properties of M‐BTC MOFs (M (Metal) =  Fe, Ni, Cu, Zn, and Pd) toward toxic SO_2_ gas have also been investigated via DFT simulations.^[^ ^225^ ^]^ The findings revealed that, with the exception of Pd‐BTC, the adsorption of SO_2_ on Zn‐BTC, Ni‐BTC, Cu‐BTC, and Fe‐BTC occurred via chemical bonding between the oxygen atom of SO_2_ and the metal atom within these MOFs. The adsorption energies were found to follow the order of Fe‐BTC > Zn‐BTC > Ni‐BTC > Cu‐BTC > Pd‐BTC. Despite the strong adsorption of Fe‐BTC toward SO_2_, its electronic properties remained largely unaffected after SO_2_ adsorption, suggesting the low suitability of Fe‐BTC and Pd‐BTC MOFs for practical SO_2_ sensing. However, experimental validation is necessary to confirm these theoretical findings.

**Figure 13:**
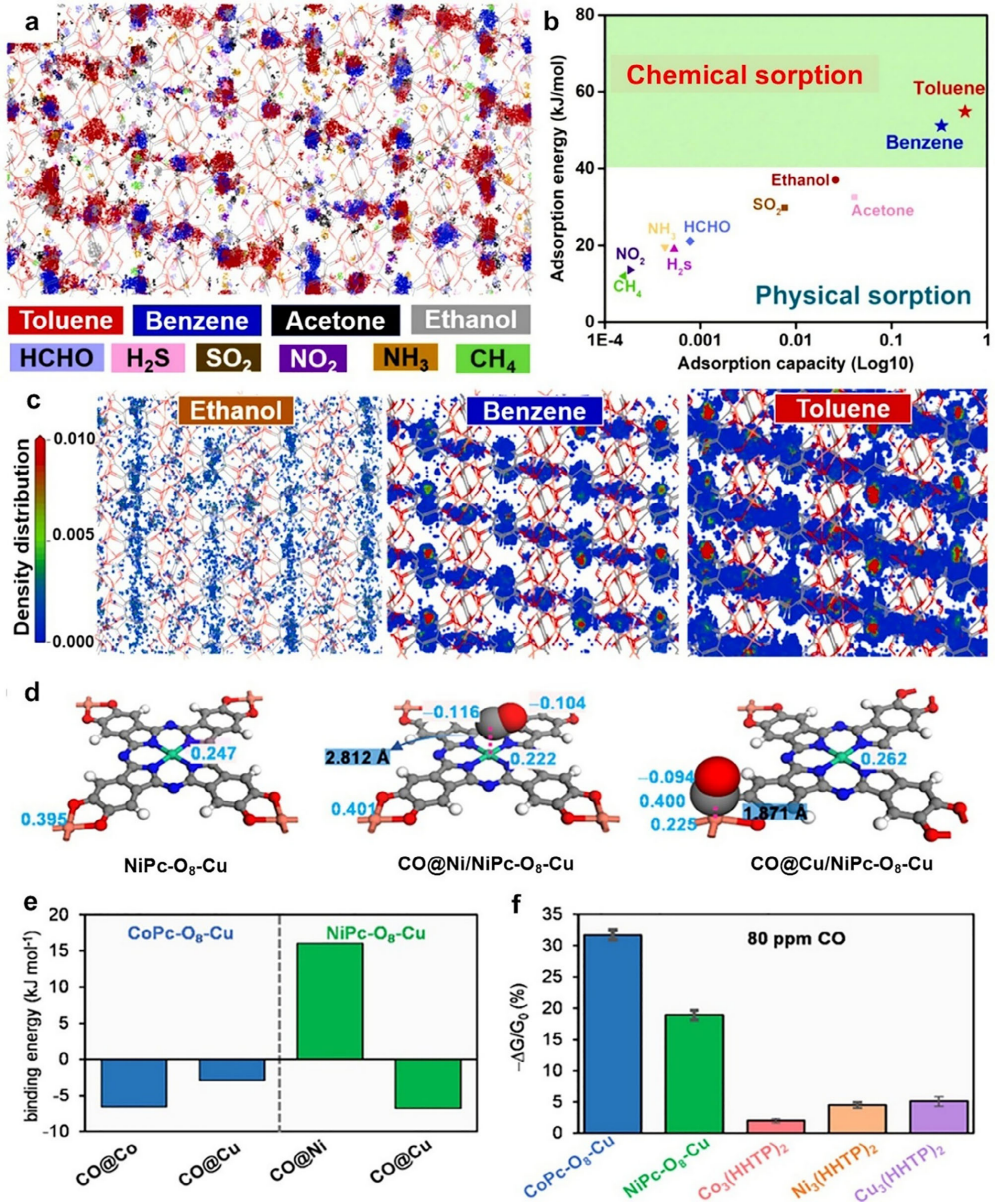
a) Distribution of the adsorption density for mixed gases within Cu‐MOF‐74. b) Relationship between the adsorption capacity and adsorption energy for nine different analytes in Cu‐MOF‐74 at a concentration of 10 ppm. c) Adsorption density maps of ethanol, benzene, and toluene within Cu‐MOF‐74. Reproduced with permission.^[^ ^227^ ^]^ Copyright 2025, Elsevier. d) Optimized configurations of NiPc‐O8‐Cu, CO@Ni/NiPc‐O8‐Cu, and CO@Cu/NiPc‐O8‐Cu. The calculated Mulliken charge values are indicated in blue, whereas the CO•••M bond distances are marked in black. e) Binding free energies of CO at various adsorption sites within the MPc‐O8‐Cu MOFs. f) Comparative analysis of the sensing performance of MPc‐O8‐Cu and M_3_(HHTP)_2_ MOFs in response to 80 ppm CO in a nitrogen environment. Reproduced with permission.^[^ ^91^ ^]^ Copyright 2022, Wiley‐VCH.

Sun and colleagues investigated the role of ZIF‐7 shells on a zinc oxide core (ZnO@ZIF‐7) and a zinc oxide‒gold core (ZnO‐Au@ZIF‐7) for NO_2_ sensing via DFT calculations.^[^ ^226^ ^]^ They reported that the addition of the ZIF‐7 shell reduced the adsorption energy of NO_2_ from −1.66 eV (ZnO) to −1.86 eV (ZnO@ZIF‐7) and −3.34 eV (ZnO‐Au@ZIF‐7). These results indicated that the incorporation of ZIF‐7 helped filter large gas molecules and increased the adsorption of NO_2_ on the surfaces of both the ZnO and the ZnO‐Au cores. Ma et al. studied the adsorption of nine different gases, including CH_4_, H_2_S, ethanol, acetone, formaldehyde, NH_3_, and NO_2,_ on Cu‐MOF‐74 via both DFT and grand canonical Monte Carlo (GCMC) simulations.^[^ ^227^ ^]^ The adsorption energies followed the trend benzene > toluene > H_2_S > NH_3_ > formaldehyde > acetone > CH_4_ > ethanol > NO_2_, according to the DFT calculations. These results revealed the preferential adsorption of Cu‐MOF‐74 toward aromatic hydrocarbons, and the sensing mechanism was based on the π–π interactions between benzene molecules and the BDC ligand. Furthermore, the crystal facet of Cu‐MOF‐74 influences the strength of the interaction, with the (110) plane showing stronger toluene adsorption (−67.50 kJ mol^−1^) than the (300) plane (−65.70 kJ mol^−1^). GCMC was also employed to study the adsorption distribution of gases when the above gases (benzene + toluene + H_2_S + NH_3_ + formaldehyde + acetone + CH_4_ + ethanol + NO_2_) were mixed and exposed to Cu‐MOF‐74. As shown in **Figure** **13**a, toluene occupied most of the adsorption sites, whereas benzene occupied a few sites. Moreover, the number of adsorbed toluene or benzene molecules was significantly greater (hundreds to thousands of times greater) than that of other small molecules, indicating the good selectivity of Cu‐MOF‐74 toward these aromatic hydrocarbons. Furthermore, the GCMC calculations revealed two adsorption types on the basis of the adsorption energies: (i) physical adsorption, which encompasses CH_4_, H_2_S, NH_3_, NO_2_, and formaldehyde, and (ii) weak chemical adsorption, which includes toluene and benzene (Figure 13b). Among the tested gases, toluene was preferentially adsorbed owing to its highest adsorption energy, indicating the good sensitivity of Cu‐MOF‐74 to this compound. This observation was further supported by analyzing the adsorption density distributions of ethanol, benzene and toluene in Cu‐MOF‐74 at identical concentrations of 1 ppm and partial pressures of 0.1 Pa. As depicted in Figure 13c, ethanol adsorption in Cu‐MOF‐74 was uniformly distributed and relatively low (indicated in blue), indicating a low probability of ethanol adsorption and a lack of significant recognition. In contrast, benzene and toluene resulted in higher adsorption densities, with a clear segregation effect, resulting in greater adsorption near the primary adsorption sites (indicated in red).

In bimetallic MOFs, understanding the role of different metals in promoting the adsorption of gases is crucial for designing effective sensors based on these materials. Recently, Najib and co‐workers investigated the adsorption of CO_2_, NO_2_, and water on M/Zn‐MOF‐74 (M  =  Be, Mg, Ca, Sr, Ba). These findings revealed that these bimetallic MOFs have a preferential adsorption order of NO_2_ > water > CO_2_.^[^ ^90^ ^]^ Among the studied M/Zn‐MOF‐74, Ca/Zn‐MOF‐74 showed the strongest adsorption for NO_2,_ with an adsorption energy of −99.1 kJ mol^−1^, followed by Sr/Zn‐MOF‐74 (−95.8 kJ mol^−1^), Ba/Zn‐MOF‐74 (−83.5 kJ mol^−1^), Mg/Zn‐MOF‐74 (−77.7 kJ mol^−1^), and Be/Zn‐MOF‐74 (−26.6 kJ mol^−1^). In comparison, the trend in adsorption energy for CO_2_ followed the order of Ca/Zn‐MOF‐74 (−40.0 kJ mol^−1^) > Sr/Zn‐MOF‐74 (−38.4 kJ mol^−1^), Ba/Zn‐MOF‐74 (−38.3 kJ mol^−1^) > Mg/Zn‐MOF‐74 (−30.3 kJ mol^−1^) > Be/Zn‐MOF‐74 (−21.1 kJ mol^−1^). Moreover, the adsorption energy for water followed the trend Mg/Zn‐MOF‐74 (−84.0 kJ mol^−1^) > Be/Zn‐MOF‐74 (−79.4 kJ mol^−1^), Ca/Zn‐MOF‐74 (−75.2 kJ mol^−1^) > Sr/Zn‐MOF‐74 (−74.6 kJ mol^−1^) > Ba/Zn‐MOF‐74 (−70.4 kJ mol^−1^). The significant difference in adsorption energy between Ca/Zn‐MOF‐74 and Be/Zn‐MOF‐74 for CO_2_ is also reflected by the difference in charge density. When CO_2_ interacted with Be/Zn‐MOF‐74, a small amount of charge accumulation was observed around the oxygen atom of the CO_2_ molecule. In contrast, CO_2_ adsorption on Ca/Zn‐MOF‐74 resulted in significantly greater charge accumulation around the oxygen atom. Similarly, the strong adsorption of NO_2_ on Ca/Zn‐MOF‐74 was supported by the substantial charge accumulation around the NO_2_ molecule. For water adsorption on Mg/Zn‐MOF‐74, charge accumulation was primarily observed around the oxygen atom of the water molecule. These results revealed the differing affinities of various metals toward CO_2_, NO_2_, and water, underscoring the importance of carefully selecting the secondary metal in bimetallic MOFs. DFT calculations were previously performed to explore the adsorption mechanisms of TMA, dimethylamine (DMA), NH_3_, and formaldehyde on a bimetallic LaCe‐MOF.^[^ ^228^ ^]^ The calculated adsorption energies for TMA, DMA, NH_3_, and formaldehyde on the LaCe‐MOF were −0.4329 eV, −0.5204 eV, −0.6823 eV, and −0.7576 eV, respectively, indicating that these gases were primarily physisorbed onto the MOF surface.

The influence of secondary metals (Ni and Co) on the adsorption of CO by 2D conductive Cu‐MOFs with integrated metallophthalocyanine (MPc) units (MPc‐O_8_‐Cu) was studied by Aykanat and co‐workers.^[^ ^91^ ^]^ Using diffuse reflectance infrared Fourier transform spectroscopy (DRIFTS), they determined that the Cu‐bis(dioxolene) units in MPc‐O_8_‐Cu served as primary host sites for CO sensing and that the incorporation of Co led to structural distortion of these units. Additionally, the authors employed DFT calculations to elucidate the superior CO‐sensing performance of CoMPc‐O_8_‐Cu compared with that of NiMPc‐O8‐Cu. Their computational results indicated that the CO‐binding behavior in CO‐bound NiPc‐O_8_‐Cu was similar to that in CoPc‐O_8_‐Cu. However, the distances between Co/Cu and CO were much shorter than those between CO and Ni/Cu ions (Figure 13d). The calculated free energies for CO binding at the Co and Cu sites in CoPc‐O_8_‐Cu were −6.5 and −2.9 kJ mol^−1^, respectively. In contrast, the corresponding energies for the Ni and Cu sites in NiPc‐O_8_‐Cu were 16.0 and −6.8 kJ mol^−1^, respectively (Figure 13e). Therefore, CO adsorption was energetically more favorable at the Co and Cu sites than at the Ni site, leading to the superior sensing performance of CoMPc‐O_8_‐Cu toward CO gas, as validated by experiments (Figure 13f).

**Figure 14:**
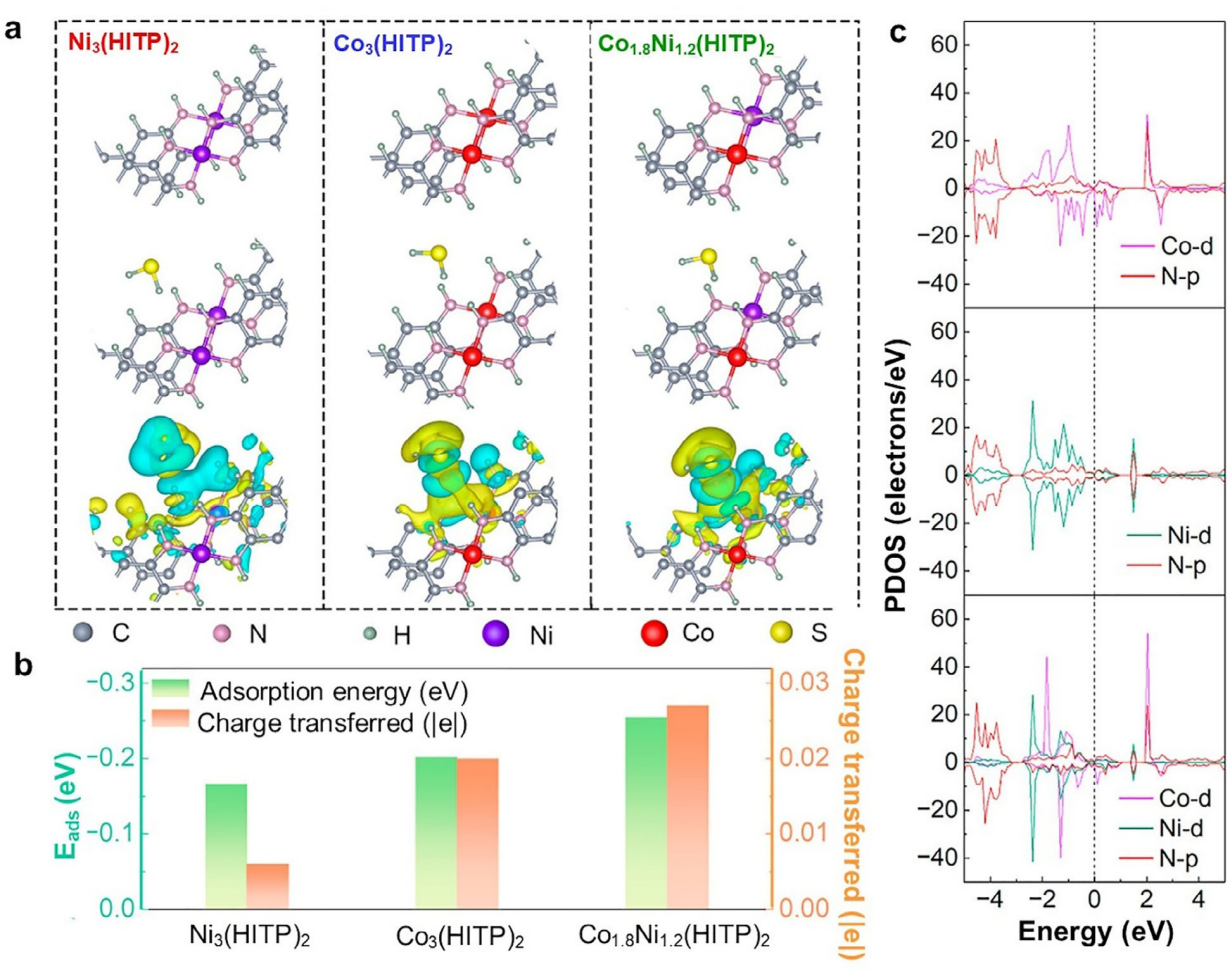
a) Optimized structures and differential charge density plots of Ni_3_(HITP)_2_, Co_3_(HITP)_2_, and Co_1.8_Ni_1.2_(HITP)_2_, where the yellow and blue regions represent charge accumulation and depletion, respectively. b) Diagrams illustrating the adsorption energy and charge transfer for the three MOFs. c) PDOS calculations for Ni_3_(HITP)_2_, Co_3_(HITP)_2_, and Co_1.8_Ni_1.2_(HITP)_2_, considering both spin‐up and spin‐down configurations. Reproduced with permission.^[^ ^51^ ^]^ Copyright 2024, American Chemical Society.

Recently, Zhao et al. investigated the H_2_S sensing mechanisms of bimetallic Co_1.8_Ni_1.2_(HITP)_2_ and its monometallic counterparts (Ni_3_(HITP)_2_ and Co_3_(HITP)_2_) (HITP = 2,3,6,7,10,11‐hexaaminotriphenylene)) via DFT simulations by analyzing their adsorption energies and charge transfer properties.^[^ ^51^ ^]^ Co_1.8_Ni_1.2_(HITP)_2_ exhibited significantly greater electron transfer upon H_2_S adsorption than Ni_3_(HITP)_2_ and Co_3_(HITP)_2_. Bader charge analysis revealed that Co_1.8_Ni_1.2_(HITP)_2_ transferred 0.027 e^−^, which is notably greater than that of Ni_3_(HITP)_2_ (0.006 e^−^) and Co_3_(HITP)_2_ (0.020 e^−^) (**Figure** **14**a). These findings underscore the synergistic effect of Ni and Co in enhancing charge transfer and chemical interactions during the sensing process. This trend was further supported by the higher adsorption energy of H_2_S on Co_1.8_Ni_1.2_(HITP)_2_ (−0.255 eV) than on Ni_3_(HITP)_2_ (−0.166 eV) and Co_3_(HITP)_2_ (−0.202 eV) (Figure 14b). Additionally, projected density of states (PDOS) analysis revealed a narrower p‐d orbital overlap range in Co_1.8_Ni_1.2_(HITP)_2_ than in Co_3_(HITP)_2_ and Ni_3_(HITP)_2_, implying reduced atomic interaction (Figure 14c). Consequently, the adsorption process involved a direct interaction with H_2_S, and electron transfer occurred without relying on oxygen. Overall, these results reinforced the stronger interaction and superior charge transfer capability of this bimetallic system. A similar conclusion was drawn by Ou's group, who reported the positive impact of bimetallic composition on gas‐sensing performance.^[^ ^53^ ^]^ They reported that the adsorption energies of NO_2_ on bimetallic Fe_2_Mn PCN‐250 (−28.79 kcal mol^−1^) and Fe_2_Co PCN‐250 (−18.64 kcal mol^−1^) were greater than those of Fe_3_ PCN‐250 (−16.71 kcal mol^−1^). The stronger interaction between Fe_2_Mn PCN‐250 and NO_2_ was attributed to the lower electronegativity and larger ionic radius of Mn relative to those of Co and Fe.

On the basis of the above studies, it can be concluded that the inclusion of a secondary metal can enhance charge transfer and the strength of interactions with the target gas compared with those of monometallic MOFs, resulting in superior sensing performance. Furthermore, secondary metals should be carefully selected to optimize the sensing performance of bimetallic MOFs, as different metals exhibit varying affinities for different gases.

### 4.3 Application of Monometallic MOFs and MOF‐Derived Carbons in QCM Gas Sensors

Traditionally, sensing materials for QCM gas sensors have been based on polymers, metal oxides, and carbon. However, in recent years, MOFs have been increasingly used in QCM gas sensors because of their high porosity, large surface area, and highly controllable composition, structure, and pore size.^[^ ^2^, ^30^, ^229^ ^]^ To fabricate a MOF‐based QCM gas sensor, the QCM electrode is coated with an MOF layer to measure the change in mass caused by the adsorption or desorption of gas molecules by the MOF layer.^[^ ^230^ ^]^ When MOF particles are deposited on the QCM electrode, they interact with gases through reactive sites in the frameworks, improving both the detection sensitivity and selectivity. The mass of the sensor increases when the MOF layer is exposed to a specific analyte, indicating that the MOF has absorbed the analyte.^[^ ^22^, ^231^ ^]^ To date, various gases or vapors, including water (humidity),^[^ ^46^, ^232^, ^233^ ^]^ NH_3_,^[^ ^10^, ^13^ ^]^ CO_2_,^[^ ^234^ ^]^ SO_2_,^[^ ^50^ ^]^ and VOCs^[^ ^47^, ^49^, ^235^, ^236^ ^]^ have been detected via MOFs. This section discusses the research progress in the application of pure MOFs and MOF‐derived carbons in QCM gas sensors.

Humidity detection has significance in various industries, including aerospace, semiconductor, food storage, mining, meteorology, medicine, and pharmaceutical industries.^[^ ^237^, ^238^ ^]^ The term “relative humidity (RH)” is commonly used to describe the level of humidity in the surrounding environment. RH is the ratio of the amount of water vapor present in the air to the maximum amount of water vapor the air can hold at the same temperature, expressed as a percentage. During sensing tests, different types of saturated saline solutions are typically used to vary the RH.^[^ ^30^, ^239^ ^]^ Carboxylate‐based MOFs are particularly popular for humidity detection because their abundant carboxyl groups easily form hydrogen bonds with water molecules during adsorption. For example, Kosuru and co‐workers developed an HKUST‐1‐coated QCM sensor for humidity sensing.^[^ ^232^ ^]^ They reported that the uncoated, PVP‐coated, and HKUST‐1‐coated QCM sensors presented Δ*f* values of 7, 48, and 720 Hz, respectively, within the 22–69% RH range, indicating the high sensitivity of HKUST‐1 to water vapor. This high sensitivity was attributed to the high adsorption energy and partial coverage of the HKUST‐1 surface by NH_3_ molecules. Additionally, a highly sensitive humidity sensor based on a composite film of carbon nanotubes (CNTs) and HKUST‐1 (CNTs‐HKUST‐1) was fabricated via the spin coating method.^[^ ^46^ ^]^ The hybridization of HKUST‐1 with CNTs (0.5 mg) significantly improved its sensitivity by 230% relative to that of pure HKUST‐1, achieving an average sensitivity of 141 Hz/% RH. This increase was promoted by the increased number of adsorption sites provided by the CNTs. However, optimizing the amount of CNTs was crucial, as their lower affinity for water could reduce the sensing performance. The CNT‐HKUST‐1 sensor also displayed a reasonable response time of <4.2 minutes and a recovery time of <4.4 minutes. Additionally, it showed good long‐term stability, with minor changes in Δ*f* over a 10‐day period.

**Figure 15:**
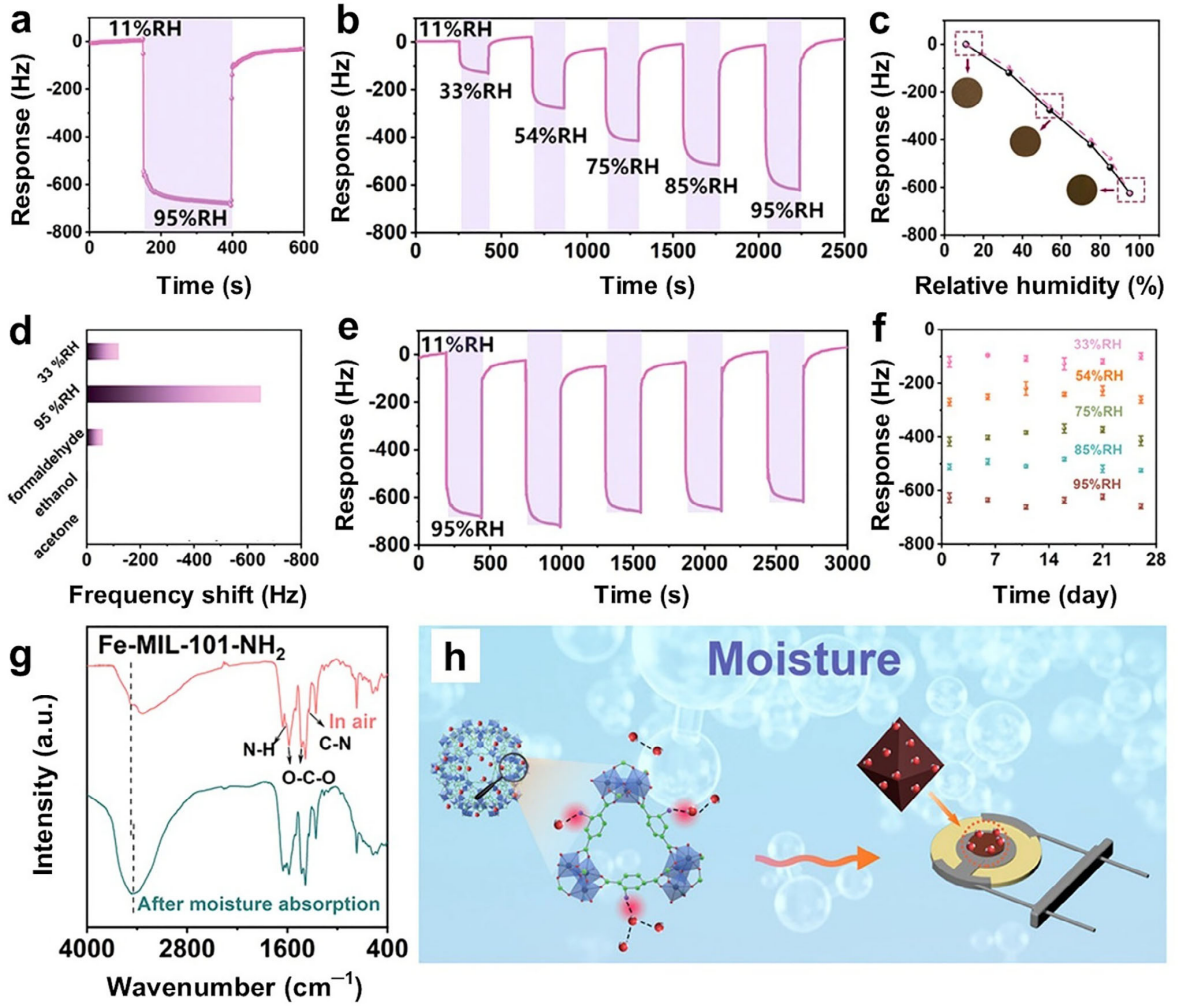
a) Dynamic response‒recovery curve of the QCM‐humidity sensor based on Fe‐MIL‐101 NH_2_ under RH conditions ranging from 11% to 95%. b) The frequency variation of the Fe‐MIL‐101 NH_2_‐based QCM sensor across varying RH levels from 11% to 95% and c) the corresponding humidity hysteresis response. d) Frequency shifts of the Fe‐MIL‐101 NH_2_‐based QCM sensor upon exposure to different gases, including acetone, ethanol, and formaldehyde. e) Reproducibility and f) long‐term stability tests of the Fe‐MIL‐101 NH_2_‐based QCM sensor toward varying RH levels from 11% to 95%. g) FTIR spectra of Fe‐MIL‐101‐NH_2_ before and after moisture absorption. h) A schematic representation of the humidity‐sensing mechanism of Fe‐MIL‐101‐NH_2_. Reproduced with permission.^[^ ^240^ ^]^ Copyright 2022, Wiley‐VCH.

In addition to carboxylic acid‐based MOFs, a 3D mixed‐valent Cu^I^/Cu^II^ MOF constructed by connecting 1D [Cu^I^(4,4′‐bipy)]*_n_* chains with [Cu^II^(oda)_2_]^2−^ cationic subunits has also been used for humidity sensing.^[^ ^233^ ^]^ This MOF exhibited Δ*f* values of 1098, 190, 114, 0, and 0 Hz upon exposure to 10 ppm water, methanol, ethanol, acetone, and dichloromethane, respectively. The superior sensitivity and selectivity of this MOF toward water vapor over organic vapors are attributed to strong hydrogen bonding interactions between polar water molecules and the uncoordinated carboxyl oxygen atoms in the framework. Additionally, the mixed‐valent Cu^I^/Cu^II^ MOF showed high Δ*f* values of 271, 444, 872, 1492, and 1996 Hz upon exposure to 2, 4, 8, 12, and 16 ppm water vapor, respectively, highlighting its excellent sensitivity. Despite these advantages, the response and recovery times of this mixed‐valent Cu‐MOF may require further optimization to increase its practicality as a humidity sensor. Fe‐MIL‐101‐NH_2_ particles with octahedral morphologies have also been explored for humidity sensing.^[^ ^240^ ^]^ These particles had a large Δ*f* of 650 Hz with increasing RH from 11% to 95%, with a rapid response/recovery time of 16 s/15 s (**Figure** **15**a). From the dynamic response‒recovery curves obtained at different RH levels (Figure 15b), a linear relationship between Δ*f* and the RH level was observed (Figure 15c). Furthermore, these octahedral Fe‐MIL‐101‐NH_2_ particles displayed excellent selectivity for water vapor relative to other organic vapors, such as formaldehyde, acetone, and ethanol (Figure 15d). Additionally, they showed good reproducibility over 5 cycles and a consistent response over a period of 28 days (Figure 15e, f). Postadsorption FTIR analysis revealed a shift in the amine vibration peak from ≈3500 to 3451 cm^−1^ (Figure 15g). Therefore, humidity sensing originates from hydrogen bonding between the amino group of Fe‐MIL‐101‐NH_2_ and water molecules, as illustrated in Figure 15h.

In addition to being used for humidity sensing, MOFs have also been used for detecting VOCs via QCM technology. For example, Chappanda et al. previously developed a ZIF‐8‐based QCM gas sensor for detecting acetone, a hazardous substance commonly used in the production of plastics, fibers, and drugs.^[^ ^235^ ^]^ Exposure to acetone can cause narcosis, nausea, and headaches.^[^ ^241^ ^]^ The ZIF‐8‐coated QCM gas sensor exhibited high stability for acetone sensing, with steady responses over a month, which was attributed to its homogeneous and dense structure.^[^ ^235^ ^]^ However, this sensor was only able to detect acetone at very high concentrations, with a Δ*f* value of 200 Hz at 265000 ppm of acetone, and suffered from a long response time (1 h). In comparison, the ZIF‐90‐based QCM gas sensor made by Zhang and co‐workers showed superior performance for acetone sensing, with a high Δ*f* value of 95 Hz and a fast response/recovery time of 12 s/17 s upon exposure to 1 ppm acetone.^[^ ^47^ ^]^ This sensor also displayed high selectivity toward acetone over other vapors, including acetone, formaldehyde, ethanol, CO_2_, NO, NO_2_, benzene, and toluene. Additionally, it can detect acetone vapor in the low concentration range of 0.5–10 ppm, with a limit of detection (LoD) of 13.7 ppb. The adsorption enthalpy of this MOF for acetone was determined to be −57.93 kJ mol^−1^ from temperature‐dependent experiments, indicating reversible chemisorption of acetone. Compared with ZIF‐90, the UiO‐66‐based QCM gas sensor reported by Ito et al. exhibited a lower Δ*f* value of ≈12 Hz toward 1 ppm acetone.^[^ ^48^ ^]^ Despite its lower sensitivity, the sensor showed a highly reversible shift in resonant frequency when exposed to a wide range of acetone concentrations from 150 ppb to 3 ppm.

Davydovskaya et al. studied the sensing performance of a QCM gas sensor coated with HKUST‐1 for detecting three different flammable VOCs: methanol, ethanol, and *n*‐propanol (2–50 ppm).^[^ ^49^ ^]^ They reported that the alcohol chain length (C_1_–C_3_), geometry (1‐propanol, 2‐propanol), and polarity significantly affect the sensing performance of alcohol. This study revealed preferential adsorption of short‐chain alcohols over long‐chain alcohols in dry air, with Δ*f* values of 226.5, 196.1, 79.5, and 38.4 Hz for methanol, ethanol, 1‐propanol, and 2‐propanol, respectively, at 0% RH. In contrast, in humid air, the HKUST‐1‐coated QCM gas sensor exhibited better sensing performance for long‐chain alcohols than for short‐chain alcohols. This change was attributed to different adsorption mechanisms: in dry air, alcohols bound specifically to unsaturated open metal sites on HKUST‐1, whereas in humid air, alcohols weakly physiosorbed through interactions with water molecules adsorbed on the sensor surface. Two MOFs with mixed ligands of carboxylate and bipyridine, [Cu_2_(OH)(2,2′‐bpy)_2_(BTC)·2H_2_O]*_n_* and [Co(4,4′‐bpy)(m‐BDC)]*_n_*, have also been explored for methanol sensing.^[^ ^236^ ^]^ The [Cu_2_(OH)(2,2′‐bpy)_2_(BTC)·2H_2_O]*_n_* MOF displayed a high Δ*f* value of ≈420 Hz toward 160 ppm of methanol, whereas [Co(4,4′‐bpy)(m‐BDC)]*_n_* exhibited an impressive Δ*f* value of ≈1400 Hz toward 16 ppm of methanol. The superior methanol‐sensing performance of [Co(4,4′‐bpy)(m‐BDC)]*_n_* was attributed to its higher solvent‐accessible volume and hydrophilic nature. The porous MOF [Cu_4_(OH)_2_(tci)_2_(bpy)_2_]·11H_2_O], created using two different ligands, tri(2‐carboxyethyl)‐isocyanurate (H_3_tci) and bpy, was previously explored for the detection of polar molecules (water, methanol, ethanol, acetone, acetonitrile) and nonpolar molecules (benzene, methylbenzene, ethylbenzene and chlorobenzene).^[^ ^242^ ^]^ It exhibited high Δ*f* values in the range of 1600–6100 Hz for polar molecules, whereas the Δ*f* values for nonpolar molecules were much lower (<2000 Hz) under ambient conditions and 65% RH. The high sensing performance of this MOF for polar molecules was attributed to its large void space and hydrophilic nature, which facilitated the formation of hydrogen bonds with these molecules.

Previously, a MIL‐101(Cr)‐coated QCM gas sensor was developed for detecting various VOCs, including methanol, ethanol, isopropanol, acetone, *n*‐hexane, dichloromethane, chloroform, pyridine, and THF, under a nitrogen atmosphere under ambient conditions.^[^ ^243^ ^]^ This MOF sensor exhibited the highest sensitivity to pyridine (2.793 Hz ppm^−1^), followed by 2‐propanol (0.648 Hz ppm^−1^), chloroform (0.524 Hz ppm^−1^), ethanol (0.429 Hz ppm^−1^), methanol (0.306 Hz ppm^−1^), THF (0.205 Hz ppm^−1^), *n*‐hexane (0.164 Hz ppm^−1^), dichloromethane (0.155 Hz ppm^−1^), and acetone (0.114 Hz ppm^−1^). Furthermore, it showed a high Δ*f* value of ≈145 Hz toward 50 ppm of pyridine, along with short response/recovery times of ≈8–24 s/≈61–118 s toward 5–700 ppm of pyridine. Additionally, the MIL‐101(Cr)‐coated QCM gas sensor showed a good LoD of 1.603 ppm for pyridine detection and excellent long‐term stability after 2 months of testing. The sensing mechanism was largely based on the strong π–π stacking interaction between pyridine and the aromatic rings in the MIL‐101(Cr) ligand. Compared with MIL‐101(Cr), the Al(OH)(1,4‐NDC) MOF showed superior sensing performance toward pyridine, as demonstrated by Xu and co‐workers.^[^ ^244^ ^]^ This MOF displayed an impressive Δ*f* value of ≈2200 Hz toward 20 ppm of pyridine and showed good selectivity for pyridine over water, methanol, ethanol, acetone, and THF. The recovery ratios for pyridine vapor detection over 32 days ranged from 97.5–108.7%, with a relative standard deviation (RSD) below 2%, indicating excellent long‐term stability. The strong adsorption of pyridine on Al(OH)(1,4‐NDC) was promoted by the formation of hydrogen bonds between the hydroxyl groups in this MOF and the nitrogen atom of pyridine (OH···N), as supported by their theoretical calculations.

In a separate study by Huang et al., an MIL‐101(Cr)‐based QCM gas sensor was used for the detection of n‐butylamine and n‐hexane.^[^ ^245^ ^]^ This sensor exhibited Δ*f* values of ≈46 Hz and ≈10 Hz upon exposure to 331 ppm n‐butylamine and 750 ppm n‐hexane, respectively. The adsorption of n‐butylamine was hypothesized to occur at unsaturated metal sites, which act as electron acceptors, whereas heteroatoms in n‐butylamine serve as electron donors. Additionally, adsorption occurred within the pores of the MOF. In contrast, for n‐hexane, the adsorption took place solely within the pores of MIL‐101(Cr), resulting in a weaker response. The sensing performance of a 3D calcium‐based MOF, Ca‐SBF (SBF = 9,9′‐Spirobi[9*H*‐fluorene]), for toluene was studied by Fang and colleagues.^[^ ^246^ ^]^ They reported that the Ca‐SBF‐based QCM gas sensor exhibited Δ*f* values of 3240, 1350, 886, and 330 Hz in response to toluene, ethanol, acetone, and benzene, respectively, indicating its high selectivity for toluene. Additionally, the sensor demonstrated a fast response and recovery time of <60 s, along with a detection limit in the ppm range. The sensing process was largely driven by the π–π interactions between the benzene ring of toluene and the aromatic rings in the Ca‐SBF framework. In comparison, the Cu_3_(BTC)_2_‐coated QCM gas sensor reported by Kimura's group showed relatively similar performance for toluene sensing to the Ca‐SBF‐based QCM gas sensor, with a Δ*f* value of 3000 Hz at 60 °C and a LoD of ≈1 ppm.^[^ ^247^ ^]^ This group also explored the effect of operating temperature on the toluene‐sensing performance of a Cu_3_(BTC)_2_‐coated QCM gas sensor. They reported a decrease in the sensing performance as the temperature increased from 20 to 60 °C. However, they also reported that toluene desorption was faster at higher temperatures.

**Figure 16:**
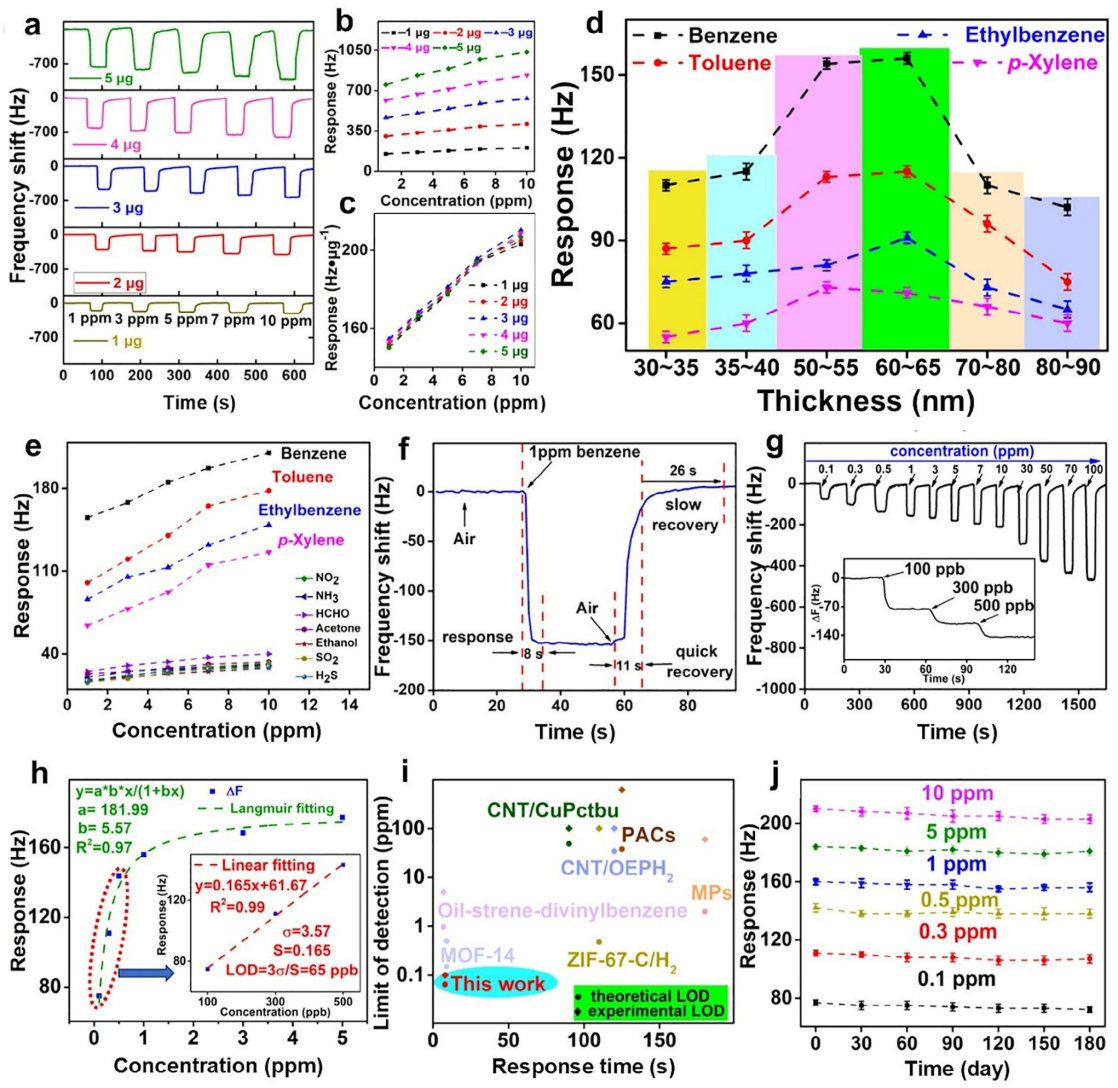
a) Response‒recovery profiles of the QCM sensor coated with varying amounts of Cu‐TCPP‐Cu‐12 upon exposure to varying concentrations of benzene from 1 to 10 ppm. b) Sensor responses of Cu‐TCPP‐Cu‐12 at various benzene vapor concentrations (1–10 ppm). c) Normalized response curves for five different sensors. d) Sensitivity of Cu‐TCPP‐Cu‐12 with varying film thicknesses toward 1 ppm of various vapors. e) Response of the Cu‐TCPP‐Cu‐12‐based QCM sensor to different vapors within the 1–10 ppm range. f) Transient response behavior of the Cu‐TCPP‐Cu‐12 sensor upon exposure to 1 ppm benzene. g) Dynamic response‒recovery curves of the Cu‐TCPP‐Cu‐12 sensor at various benzene concentrations (the inset shows consecutive response curves for concentrations between 100 and 500 ppb). h) Langmuir adsorption fitting for benzene, with the inset displaying a linear fit at low benzene concentrations. i) LoD of the Cu‐TCPP‐Cu‐12‐based QCM sensor as a function of response time. j) Long‐term stability evaluation of the Cu‐TCPP‐Cu‐12‐based QCM sensor for benzene detection at concentrations ranging from 0.1 to 10 ppm. Reproduced with permission.^[^ ^248^ ^]^ Copyright 2024, American Chemical Society.

Recently, Xu's group studied the effects of loading and thickness of 2D Cu‐TCPP‐Cu MOF nanosheets on their sensing performance toward benzene.^[^ ^248^ ^]^ The response of this 2D MOF sensor increased with increasing MOF loading from 1 to 5 µg (**Figure** **16**a,b), with the optimum sensing performance achieved using 3 µg after normalization (Figure 16c). Furthermore, a volcano‐shaped relationship was observed between the thickness of the MOF nanosheets and their response to benzene and other vapors (toluene, ethylbenzene, and p‐xylene). A thickness range of 60–65 nm was found to be optimal for achieving the best sensing performance toward most vapors (including benzene), except for p‐xylene (Figure 16d). The dependence of the sensing performance of the 2D Cu‐TCPP‐Cu MOF nanosheets on concentration is clearly illustrated in Figure 16e. The responses of these MOF nanosheets gradually increased with increasing vapor concentration for all analytes. The highest sensing performance was observed for benzene, with the 2D Cu‐TCPP‐Cu MOF nanosheets displaying a high Δ*f* value of ≈160 Hz toward 1 ppm benzene. Moreover, this 2D MOF sensor exhibited a fast response/recovery time of 8 s/11 s when exposed to 1 ppm benzene (Figure 16f). From the dynamic response‒recovery curves (Figure 16g), a linear calibration curve was obtained between the benzene concentration and response, and the LoD was estimated to be 65 ppb (Figure 16h). The benefit of the 2D structure was evident from Figure 16i, where the performance of the Cu‐TCPP‐Cu MOF nanosheets was superior to that of 3D ZIF‐67 particles for benzene sensing, while excellent long‐term stability was maintained over a 6‐month period. (Figure 16j).

In addition to VOCs, MOF‐based QCM gas sensors can also be employed for detecting toxic gases. Hupp and co‐workers fabricated a HKUST‐1‐silica colloidal crystal thin film on a QCM electrode for carbon disulfide (CS_2_) detection.^[^ ^249^ ^]^ Exposure to CS_2_ can cause nausea, vomiting, and diarrhea and damage the nervous, reproductive, and respiratory systems.^[^ ^250^, ^251^ ^]^ The HKUST‐1‐silica thin film responded rapidly and reversibly to CS_2_, but it exhibited relatively low sensitivity and poor selectivity, with cross‐sensitivity to other vapors, such as water and ethanol. Hence, further modifications are needed to further enhance the sensing performance of HKUST‐1 toward CS_2_.

CO_2_ is one of the most common greenhouse gases, and monitoring its concentration is crucial for food safety, air quality, and early fire detection.^[^ ^252^, ^253^ ^]^ A dense ZIF‐8 crystal film was employed by Devkota et al. to detect CO_2_ via the QCM technique.^[^ ^234^ ^]^ The twice‐coated ZIF‐8‐based QCM sensor showed a Δ*f* value of 217 Hz in response to 100 vol% CO_2_. The sensor exhibited a linear relationship between Δ*f* and the CO_2_ concentration, yielding a sensitivity of 2.18 Hz/vol.% (∼0.24 × 10^−6^/vol.%). Interestingly, the sensitivity of this ZIF‐8 sensor to methane (CH_4_) was 23.6 times lower than that to CO_2_. This significant difference in sensitivity was attributed to the heavier nature and smaller size of CO_2_ than the cage size of ZIF‐8, allowing for more effective adsorption.

SO_2_ is a gaseous pollutant commonly released from the combustion of fossil fuels and volcanic activity. Exposure to this gas can lead to various health effects, ranging from respiratory irritation to decreased lung function due to respiratory diseases.^[^ ^254^ ^]^ Fluorinated MOFs, specifically KAUST‐7 and KAUST‐8 (KAUST = King Abdullah University of Science and Technology), have demonstrated exceptional sensitivity in detecting SO_2_.^[^ ^50^ ^]^ These MOFs were able to detect SO_2_ concentrations as low as 15 ppb. Interestingly, the sensing performance of the two fluorinated MOFs varied under humid conditions. Unlike that of the KAUST‐7 sensor, the response of the KAUST‐8‐based QCM gas sensor to SO_2_ was greatly reduced in the presence of humidity (60% RH). This phenomenon was attributed to the ability of SO_2_ to displace preadsorbed water molecules on KAUST‐7 but not on KAUST‐8, which exhibited a relatively high affinity for water. Both the KAUST‐7 and KAUST‐8‐based QCM sensors demonstrated good long‐term stability when exposed to 50, 100, and 157 ppm SO_2_ over a 12‐day period.

MOFs are rich in carbon and have been widely used as precursors or templates for preparing nanoporous carbons.^[^ ^255^, ^256^, ^257^ ^]^ Previously, ZIF‐8‐derived nanoporous carbon materials have been investigated for the detection of harmful carcinogens, such as benzene and toluene. Torad and colleagues reported the first use of ZIF‐8‐derived nanoporous carbon for toluene detection via the QCM technique.^[^ ^258^ ^]^ This study revealed that the sensitivity and selectivity of ZIF‐derived carbon polyhedrons are influenced by their size and degree of graphitization. Specifically, compared with larger nanoporous carbon polyhedrons (Δ*f =* 582 Hz), small nanoporous carbon polyhedrons showed greater adsorption uptake (Δf = 727 Hz) toward toluene within a few minutes. The enhanced adsorption was attributed to the shortened diffusion pathway resulting from the decreased size of the nanoporous carbon particles.

In another study, Torad and co‐workers compared the sensing properties of nanoporous carbon materials obtained from the carbonization of both pure ZIF‐8 and bimetallic Zn‐Co ZIF in two different atmospheres, namely, nitrogen and hydrogen atmospheres.^[^ ^42^ ^]^ They reported that the use of a H_2_ atmosphere facilitated the increased formation of CNTs on the surface of nanoporous carbon polyhedrons due to the improved catalytic effect of Co NPs. Compared with their aliphatic analogs (n‐C_6_H_12_ and c‐C_6_H_12_), the nanoporous carbon polyhedrons derived from ZIF‐8 and bimetallic Zn‐Co ZIF crystals were significantly more sensitive to aromatic hydrocarbons, such as benzene and toluene. Among the samples, the ZIF‐67‐derived carbon obtained under hydrogen flow (ZIF‐67‐C/H_2_) showed the best sensing performance toward benzene and toluene, with remarkably high Δ*f* values of 14990 Hz and 15770 Hz, respectively. The exceptional sensitivity and selectivity of ZIF‐67‐C/H_2_ were attributed to the abundant *π*‐rich electrons and mesopores in this sample, which facilitated easier diffusion of these aromatic hydrocarbons into the graphitic CNT interlayers to form π–π interactions. The high selectivity of this ZIF‐67‐derived carbon suggests its potential for the molecular discrimination of aromatic hydrocarbons in urban air and chemical industries, where these compounds are commonly found.

### 4.4 Application of Bimetallic MOFs in QCM Gas Sensors

The incorporation of new secondary metal nodes in bimetallic MOFs can significantly enhance gas adsorption by providing more unsaturated metal sites to interact with gas molecules.^[^ ^5^, ^259^ ^]^ Additionally, this incorporation can create synergistic effects by enhancing charge transfer with gas molecules compared with single‐metal MOFs, as discussed earlier in Section 4.2. By properly tuning the metal ratio, bimetallic MOFs can exhibit increased surface area and porosity compared with their monometallic counterparts. As a result, they can provide more adsorption sites for gas molecules and facilitate more effective diffusion of these molecules during the sensing process. Owing to these advantageous properties, bimetallic MOFs generally exhibit superior gas‐sensing performance compared with their monometallic counterparts when used as sensing layers in QCM gas sensors, as reflected by the studies discussed in this section.

**Figure 17:**
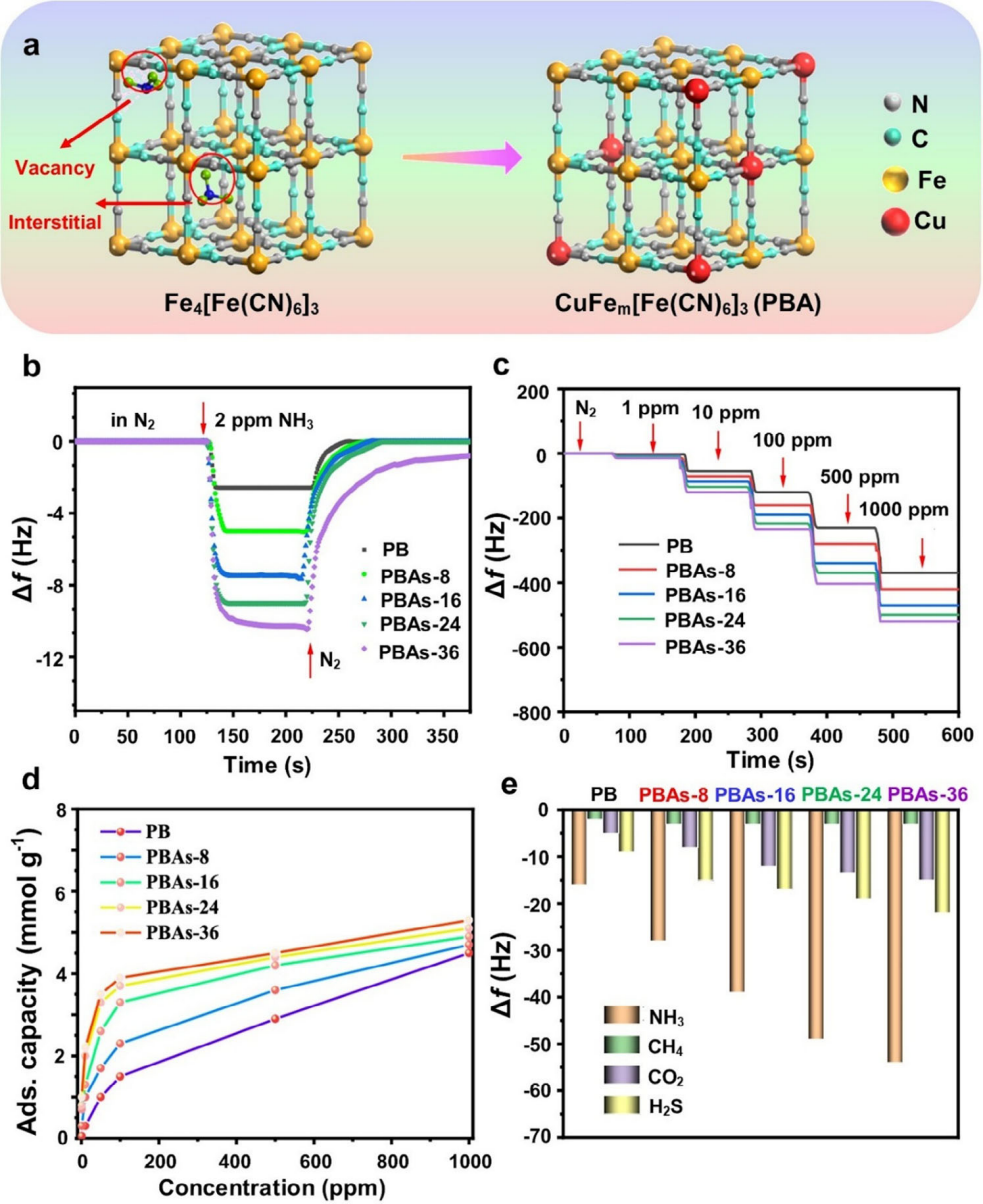
a) Crystal structure of CuFe‐PBAs. b) Adsorption‒desorption cycles of PB, PBAs‐8, PBAs‐16, PBAs‐24, and PBAs‐36 upon exposure to 2 ppm NH_3_ vapor. c) Frequency shifts of PB, PBAs‐8, PBAs‐16, PBAs‐24, and PBAs‐36 to varying concentrations of NH_3_ vapor. d) Adsorption capacity across different NH_3_ concentrations for PB, PBAs‐8, PBAs‐16, PBAs‐24, and PBAs‐36. e) Adsorption capacity of PB, PBAs‐8, PBAs‐16, PBAs‐24, and PBAs‐36 toward 5 ppm of various gases. Reproduced with permission.^[^ ^260^ ^]^ Copyright 2024, American Chemical Society.

Recently, bimetallic CuFe‐PBA nanocubes (**Figure** **17**a) were employed for the detection of NH_3_ vapor via the QCM method.^[^ ^260^ ^]^ Four different CuFe‐PBA samples, namely, PBAs‐8@QCM, PBAs‐16@QCM, PBAs‐24@QCM, and PBAs‐36@QCM, were prepared with reaction times of 8, 16, 24, and 36 h, respectively. Compared with PB (Prussian blue), the PBA‐based samples clearly showed greater sensing performance toward NH_3_ vapor, as shown in Figure 17b. Specifically, the PBA‐8, PBA‐16, PBA‐24, and PBA‐36‐based QCM gas sensors presented high Δ*f* values of 299.4, 352.3, 380.1, and 401.5 Hz, respectively, in response to 100 ppm of NH_3_ vapor (Figure 17c). In comparison, the PB‐based QCM gas sensor displayed a lower Δ*f* value of 230 Hz, confirming the benefit of introducing Cu metal into the PB framework. This trend was consistent with the trend in adsorption per unit mass of the sensing material at various concentrations (Figure 17d). For example, the adsorption capacities of PB, PBAs‐8, PBAs‐16, PBAs‐24, and PBAs‐36 toward 10 ppm of NH_3_ at 1 atm were 0.3, 1.0, 1.3, 1.9, and 2.2 mmol g^−1^, respectively. Among the PBA samples, PBAs‐36@QCM exhibited the highest sensing performance toward NH_3,_ with an excellent detection limit of 3.8 ppb. Furthermore, it showed good selectivity toward 5 ppm of NH_3_ (Δ*f* ≈55 Hz) compared with CH_4_ (Δ*f* ≈3 Hz), CO_2_ (Δ*f* ≈17 Hz), and H_2_S (Δ*f* ≈22 Hz) at the same concentration, as shown in Figure 17e. The adsorption mechanism involves two main interactions: (i) strong interactions between NH_3_ molecules and unsaturated metal sites (Fe^2+^, Fe^3+^, and Cu^2+^) and (ii) analyte‒analyte interactions, where the adsorbed NH_3_ molecules interact with the injected NH_3_ gas through hydrogen bonding. Recently, a mixed DUT‐4(Al)/MIL‐100(Fe) MOF (DUT = Dresden University of Technology) was explored for detecting α‐pinene, a terpenic VOC encountered in indoor air at a trace level.^[^ ^261^ ^]^ This mixed MOF outperformed pure MIL‐100(Fe) for *α*‐pinene sensing, with a sensitivity value of 6 × 10^−4^ log_10_(ppm)^−1^ across α‐pinene concentrations ranging from 0.6 to 100 ppm. Additionally, it exhibited a relatively fast recovery time of between 95 and 120 s when exposed to α‐pinene concentrations of 0.8 to 47 ppm.

**Figure 18:**
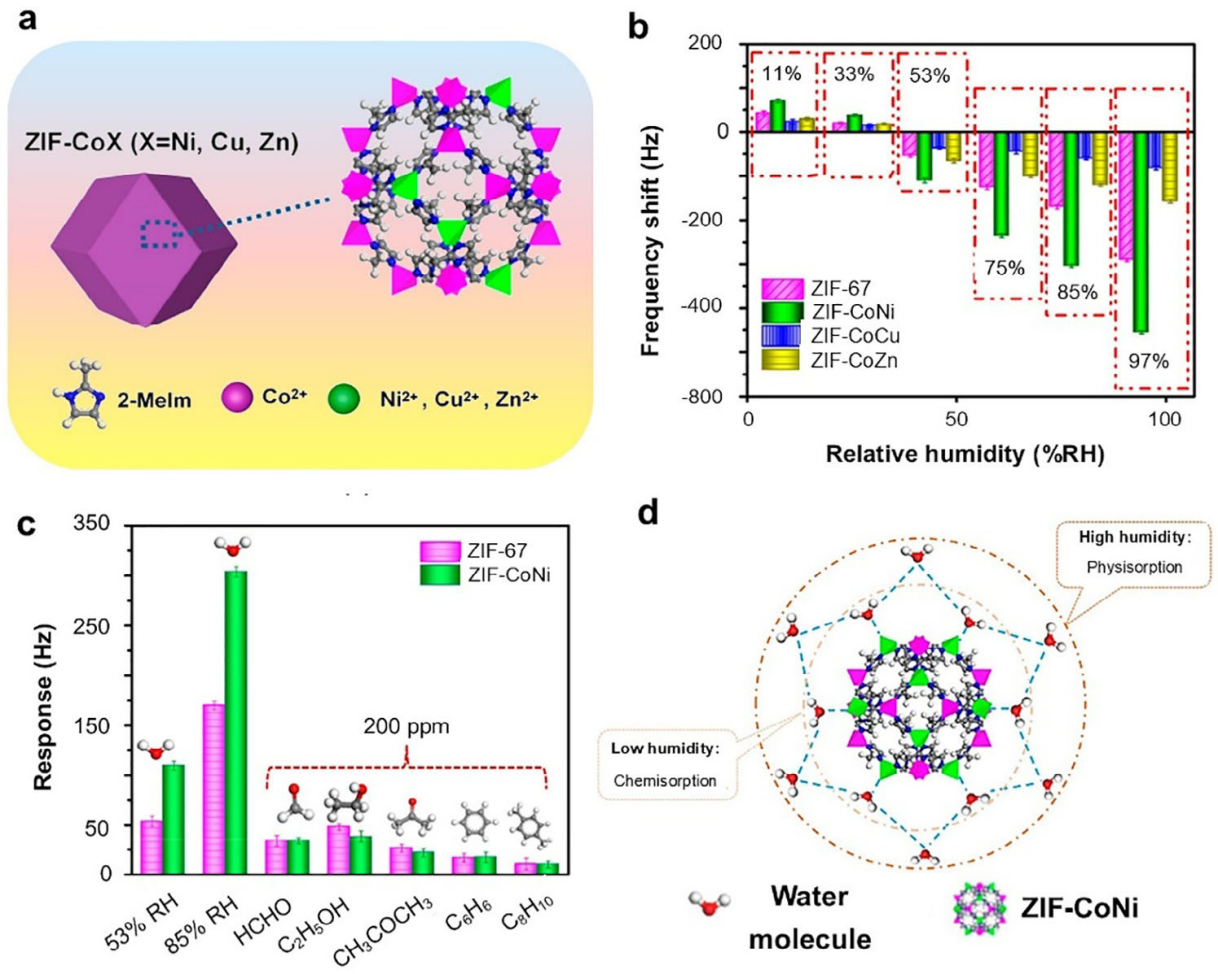
a) Schematic illustration of the synthesis of ZIF‐67 and Co‐based bimetallic ZIF‐CoX (X = Ni, Cu, Zn). b) Frequency shifts of the ZIF‐67, ZIF‐CuNi, ZIF‐CoCu, and ZIF‐CoZn‐modified QCM sensors recorded at different RH levels. c) Selectivity of humidity sensors based on ZIF‐67 and ZIF‐CoNi. d) Schematic diagram illustrating the humidity sensing mechanism of ZIF‐CoNi. Reproduced with permission.^[^ ^214^ ^]^ Copyright 2022, American Chemical Society.

The modulation of the metal centers in bimetallic MOFs is crucial for optimizing their sensitivity toward the target gas. Zhang et al. compared the humidity‐sensing performance of bimetallic ZIFs with different metal centers, including ZIF‐CoNi, ZIF‐CoCu, and ZIF‐CoZn, as shown in **Figure** **18**a.^[^ ^214^ ^]^ The study revealed that the ZIF‐CoNi sample displayed superior sensitivity (455 Hz at 97% RH, average detection sensitivity = 6.10 Hz/%RH) compared with that of the ZIF‐CoCu and ZIF‐CoZn (Figure 18b) samples. To minimize cross‐sensitivity to VOCs, the selectivity of the ZIF‐67‐ and ZIF‐CoNi‐coated QCM sensors was evaluated under 53% RH, 85% RH, and exposure to various VOCs (Figure 18c). At 200 ppm, the measured Δ*f* values for formaldehyde, ethanol, acetone, benzene and toluene were ≈35 Hz (both ZIF‐67 and ZIF‐CoNi), 50 Hz (ZIF‐67) and 40 Hz (ZIF‐CoNi), 30 Hz (ZIF‐67) and 25 Hz (ZIF‐CoNi), 25 Hz (both ZIF‐67 and ZIF‐CoNi), and 17 Hz (both ZIF‐67 and ZIF‐CoNi), respectively. Notably, the ZIF‐CoNi‐coated sensor exhibited a stronger response to water vapor than to these VOCs, indicating its superior selectivity for humidity detection over the ZIF‐67‐coated sensor. Additionally, it also exhibited a short response/recovery time (3 s/4 s, at 97% RH) and good long‐term stability. To elucidate the humidity sensing mechanism, wettability and FTIR analyses were performed, revealing that the enhanced sensitivity of the ZIF‐CoNi‐modified QCM gas sensor was due to the stronger water coordination ability and superhydrophilic nature of Ni^2+^ than those of Co^2+^, Cu^2+^, and Zn^2+^. The corresponding sensing mechanism is illustrated in Figure 18d. In low‐humidity environments, water molecules are chemisorbed on the surface of ZIF‐CoNi through coordination with Ni^2+^. In contrast, at high humidity, excess water molecules in the air lead to physisorption via hydrogen bonding. These findings imply that the hydrophilicity and hydrophobicity of the sensing material can be influenced by the presence of various open metal sites.

Apart from the selection of metal centers, the ratio of these metal centers in bimetallic MOFs plays a crucial role in determining the sensing performance. For example, bimetallic NiCo‐BTC MOFs with different Ni/Co ratios (labeled NC11‐BTC, NC12‐BTC, and NC21‐BTC, with Ni/Co = 1:1, 1:2, and 2:1, respectively) exhibited varying sensing performances toward NH_3_ vapor.^[^ ^13^ ^]^ NC12‐BTC, with a thick plate‐like morphology, presented a greater response (Δ*f* = 153.5 Hz) toward 69.5 ppm of NH_3_ than did NC11‐BTC (Δ*f*  = 61.8 Hz) and NC21‐BTC (Δ*f*  = 45.7 Hz). Furthermore, the response of NC12‐BTC toward NH_3_ vapor (69.5 ppm) increased by 1.6 times (Δ*f* = 245.5 Hz) when the nanoplates were made hollow by adding PVP during the synthesis process. In addition, these hollow NiCo‐BTC nanoplates were found to be several times more sensitive to NH_3_ vapor (69.5 ppm) than pure Ni‐BTC and Co‐BTC. The response of NC12‐BTC toward 69.5 ppm of NH_3_ (Δ*f* = 245.5 Hz) exceeded those observed for other organic vapors at the same concentration, including TMA (Δ*f* = 129.3 Hz), aniline (Δ*f* = 85.8 Hz), formaldehyde (Δ*f* = 40.4 Hz), ethanol (Δ*f* = 55.3 Hz), tetrahydrofuran (Δ*f* = 71.4 Hz), benzene (Δ*f* = 32.2 Hz), carbon tetrachloride (Δ*f* = 30.5 Hz), and water (Δ*f* = 54.4 Hz). Furthermore, the relatively small shifts for aromatic and chlorinated hydrocarbons (Δ*f* = 32.2 Hz for benzene and Δ*f* = 30.5 Hz for carbon tetrachloride) indicated a predominance of physisorption rather than chemisorption, reflecting their weaker interactions with the NiCo‐BTC framework. The good NH_3_‐sensing performance of these hollow NiCo‐BTC nanoplates was attributed to i) the presence of surface carboxyl and hydroxyl groups, which promoted the chemisorption of NH_3_ molecules, and ii) the hollow 2D structure, which provided more adsorption sites for NH_3_ molecules while simultaneously enhancing accessibility to these sites.

**Figure 19:**
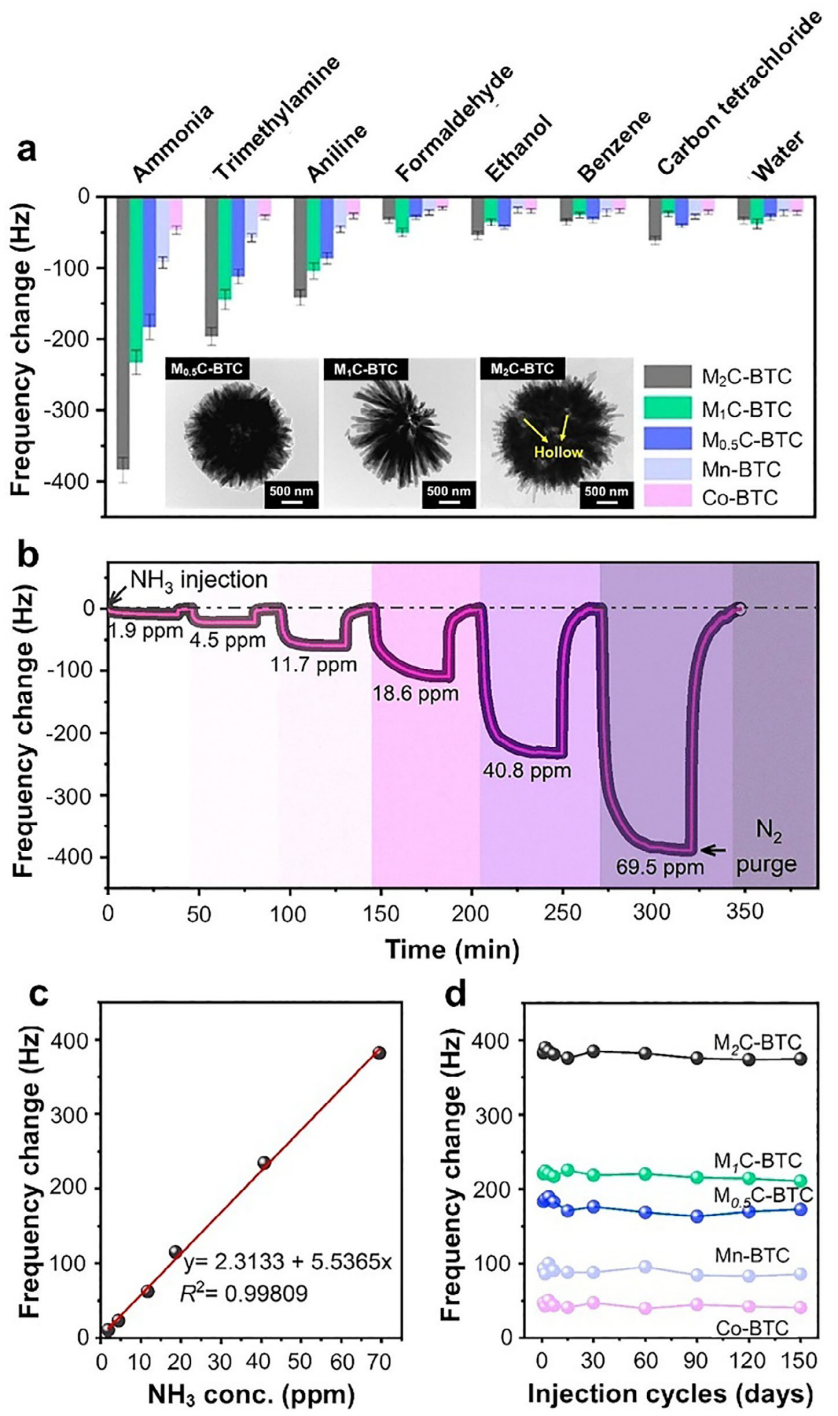
a) Frequency shifts of the different MOF‐modified QCM sensors upon exposure to 69.5 ppm of various vapors (inset: TEM images of the three bimetallic MnCo‐BTC MOFs: M_0.5_C‐BTC, M_1_C‐BTC, and M_2_C‐BTC). b) Dynamic response‒recovery curves of the M_2_C‐BTC‐based QCM sensor upon exposure to different concentrations of NH_3_ at ambient temperature and c) the corresponding linear calibration curve. d) Long‐term stability evaluations of various MOF‐coated QCM sensors toward 69.5 ppm of NH_3_ vapor over a 5‐month period. Reproduced with permission.^[^ ^10^ ^]^ Copyright 2024, Elsevier.

In a follow‐up study, Chowdhury and co‐workers investigated the NH_3_‐sensing performance of bimetallic MnCo‐BTC MOFs with different Mn/Co ratios via the QCM technique.^[^ ^10^ ^]^ Hierarchical hollow MnCo‐BTC spheres (M_2_C‐BTC) synthesized with a Mn/Co weight ratio of 1:2 displayed a higher Δ*f* (383.7 Hz) than nanorod‐assembled flower‐like MnCo‐BTC [M_1_C‐BTC; Mn/Co weight ratio = 1:1] (Δ*f * = 220.9 Hz) and nanoplate‐assembled flower‐like MnCo‐BTC [M_0.5_C‐BTC; Mn/Co weight ratio = 1:2] (Δ*f * = 183.2 Hz) upon exposure to 69.5 ppm NH_3_ vapor (**Figure** **19**a). Additionally, the M*_2_* C‐BTC sensor exhibited 4.2‐ and 8.3‐fold greater responses toward NH_3_ than did pure Mn‐BTC (Δ*f*  = 92.3 Hz) and Co‐BTC (Δ*f*  =  46.1 Hz), respectively. This increase was attributed to the increased number of unsaturated metal sites provided by the bimetallic composition, as well as the hollow structure, which improved the adsorption rate and accessibility of NH_3_ molecules to the active sites. In terms of selectivity, the M_2_C‐BTC‐coated QCM gas sensor showed the highest adsorption uptake (Δ*f* = 383.7 Hz) toward 69.5 ppm of NH_3_, significantly surpassing the responses observed for water vapor (Δ*f* = 31.6 Hz) and various organic vapors at the same concentration. These included TMA (Δ*f* = 196.1 Hz), aniline (Δ*f* = 141.8 Hz), carbon tetrachloride (Δ*f* = 65.3 Hz), ethanol (Δ*f* = 54.0 Hz), benzene (Δ*f* = 34.9 Hz), and formaldehyde (Δ*f* = 32.5 Hz). The dynamic response‒recovery curves of the M_2_C‐BTC‐coated QCM gas sensor showed a gradual increase in the sensor response with increasing NH_3_ concentration (Figure 19b). The corresponding calibration curve revealed a linear relationship between Δ*f* and the NH_3_ concentration (Figure 19c), with a LoD of 1.12 ppm. Furthermore, this bimetallic MOF sensor maintained highly stable responses toward 69.5 ppm of NH_3_ over a 5‐month period, indicating its excellent long‐term stability (Figure 19d). The pronounced selectivity of M_2_C‐BTC toward NH_3_ originated from strong chemisorptive interactions between NH_3_ molecules and unsaturated metal centers within the metal‐BTC framework.^[^ ^262^ ^]^ These findings underscore the strong affinity of metal‐BTC MOFs for basic analytes. The proposed sensing mechanism involved hydrogen bonding between the carboxyl and hydroxyl groups in the bimetallic MOF and NH_3_ molecules, along with chemical adsorption of NH_3_ on the unsaturated metal (Mn and Co) sites. A LaCe‐MOF‐modified QCM gas sensor was previously developed to detect four different gases, namely, formaldehyde, DMA, TMA, and NH_3_.^[^ ^228^ ^]^ The La:Ce ratio had a profound effect on the selectivity, with the 1:1 LaCe‐MOF exhibiting greater selectivity toward amines (TMA and DMA), whereas the 16:9 LaCe‐MOF showed higher selectivity toward formaldehyde. The 1:1 LaCe‐MOF exhibited selectivity values of 3.51, 4.19, 3.14, and 3.08 Hz/(µmoL L^−1^) for TMA, DMA, NH_3_, and formaldehyde, respectively. Theoretical calculations revealed that these gases were physisorbed (rather than chemisorbed) on the bimetallic LaCe‐MOF. To demonstrate real‐world applicability, the LaCe‐MOF‐modified QCM sensor was used to monitor the freshness of salmon meat stored at 4 °C over a 7‐day period. As the salmon decomposed, TMA and DMA gases were released, which were detected through changes in the Δ*f* value, corroborated by measurements of total volatile base nitrogen (TVB‐N) content via Kjeldahl nitrogen analysis. The salmon meat was considered fresh when the Δ*f* value was less than 62 Hz and spoiled when it exceeded this threshold. The sensor effectively detected spoilage, with the Δ*f* value rising from less than 45 Hz on day 0 to over 62 Hz after 5 days of storage. Although the LaCe‐MOF‐based QCM sensor shows strong potential for evaluating the freshness of refrigerated salmon, further enhancements in selectivity toward amines may be needed for broader practical use. Nevertheless, this study highlights the promising application of bimetallic MOFs in food quality monitoring.

**Table** **3** compares the sensing performance of MOF‐based QCM gas sensors with that of other types of gas sensors fabricated using MOFs for various analytes.^[^ ^10^, ^13^, ^46^, ^47^, ^49^, ^91^, ^214^, ^228^, ^232^, ^233^, ^234^, ^236^, ^240^, ^243^, ^244^, ^246^, ^247^, ^248^, ^261^, ^263^, ^264^, ^265^, ^266^, ^267^, ^268^, ^269^, ^270^, ^271^, ^272^, ^273^, ^274^, ^275^, ^276^, ^277^, ^278^, ^279^, ^280^, ^281^, ^282^, ^283^, ^284^, ^285^, ^286^, ^287^, ^288^, ^289^, ^290^, ^291^, ^292^ ^]^ The data indicate that MOF‐based QCM gas sensors can deliver comparable or even superior sensing performance to MOF‐based chemiresistive gas sensors while operating at relatively lower temperatures and offering faster response and recovery times. Moreover, they generally exhibit higher sensitivity and lower detection limits for VOC sensing than MOF‐based capacitive gas sensors do. Compared with MOF‐based optical gas sensors, QCM gas sensors typically offer similar LoDs but demonstrate faster response and recovery characteristics. Overall, current studies suggest that bimetallic MOFs are especially promising for detecting humidity‐ and amine‐based compounds. However, further investigations are needed to assess their performance in detecting a broader range of toxic gases and VOCs, thereby advancing their practical utilization in real‐life gas monitoring applications.

**Table 3:** Comparison of the sensing performance of MOF‐based QCM sensors for various analytes with other types of MOF‐based sensors.

MOF	Sensor type	Analyte	Sensing conditions	Response/Sensitivity	LoD [ppm]	Response/Recovery time [s]	Refs.
ZIF‐67	QCM	Water (humidity)	RT/varied RH	3.87 Hz/%RH (97% RH)	N/A	3/4 (97% RH)	^[^ ^214^ ^]^
HKUST‐1	QCM	Water (humidity)	RT/22‐69% RH	720 Hz (69% RH)	N/A	386/1051 (22‐69% RH)	^[^ ^232^ ^]^
HKUST‐1/CNT (0.5)	QCM	Water (humidity)	20 °C/5‐75% RH	141 Hz/% RH	N/A	250/265 (5‐75% RH)	^[^ ^46^ ^]^
Cu^+^/Cu^2+^‐MOF	QCM	Water (humidity)	RT	1996 Hz (16 ppm)	N/A	–/<120 (16 ppm)	^[^ ^233^ ^]^
Fe‐MIL‐101‐NH_2_	QCM	Water (humidity)	RT/11‐95% RH	650 Hz (95% RH)	N/A	16/15 (11‐95% RH)	^[^ ^240^ ^]^
ZIF‐90	QCM	Acetone	RT	95 Hz (1 ppm)	0.0137	12/17 (1 ppm)	^[^ ^47^ ^]^
HKUST‐1	QCM	Methanol	RT, 0% RH	226.5 Hz (10 ppm)	N/A	N/A	^[^ ^49^ ^]^
[Cu_2_(OH)(2,2′‐bpy)_2_(BTC)·2H_2_O]*n*	QCM	Methanol	RT	≈420 Hz (160 ppm)	N/A	N/A	^[^ ^236^ ^]^
[Co(4,4′‐bpy)(m‐BDC)]*n*	QCM	Methanol	RT	≈1400 Hz (16 ppm)	N/A	N/A	^[^ ^236^ ^]^
MIL‐101(Cr)	QCM	Methanol	30 °C	0.306 Hz ppm^−1^	10.51	8/20 (25 ppm)	^[^ ^243^ ^]^
MIL‐101(Cr)	QCM	Ethanol	30 °C	0.429 Hz ppm^−1^	8.862	10/38 (10 ppm)	^[^ ^243^ ^]^
MIL‐101(Cr)	QCM	Chloroform	30 °C	0.524 Hz ppm^−1^	4.593	4/42 (5 ppm)	^[^ ^243^ ^]^
MIL‐101(Cr)	QCM	2‐Propanol	30 °C	0.648 Hz ppm^−1^	3.056	7/30 (5 ppm)	^[^ ^243^ ^]^
MIL‐101(Cr)	QCM	Pyridine	30 °C	2.793 Hz ppm^−1^	1.603	8/61 (5 ppm)	^[^ ^243^ ^]^
Al(OH)(1,4‐NDC)	QCM	Pyridine	RT	99.7 Hz ppm^−1^	0.04	N/A	^[^ ^244^ ^]^
Ca‐SBF	QCM	Toluene	RT	3240 Hz (100 ppm)	1.0	<25/<10 (100 ppm)	^[^ ^246^ ^]^
Cu_3_(BTC)_2_	QCM	Toluene	60 °C	3000 Hz (100 ppm)	1.0	N/A	^[^ ^247^ ^]^
Cu‐TCPP‐Cu	QCM	Benzene	RT	≈155 Hz (1 ppm)	0.065	8/11 (1 ppm)	^[^ ^248^ ^]^
ZIF‐8	QCM	CO_2_	RT	2.18 Hz/vol.%	N/A	N/A	^[^ ^234^ ^]^
MOF‐14	QCM	p‐Xylene	RT/40% RH	341 Hz (1 ppm)	0.057	8/10 (1 ppm)	^[^ ^263^ ^]^
ZIF‐CoNi	QCM	Water (humidity)	RT/11‐97% RH	6.10 Hz/%RH (97% RH)	N/A	3/3 (97% RH)	^[^ ^214^ ^]^
DUT‐4(Al)/MIL‐100(Fe)	QCM	*α*‐Pinene	30 °C	6.0 × 10^−4^ log_10_(ppm)^−1^	N/A	N/A	^[^ ^261^ ^]^
MnCo‐BTC	QCM	NH_3_	20 °C/43% RH	383.7 Hz (69.5 ppm)	1.12	N/A	^[^ ^10^ ^]^
NiCo‐BTC	QCM	NH_3_	20 °C/47% RH	245.5 Hz (69.5 ppm)	1.53	N/A	^[^ ^13^ ^]^
La‐Ce MOF	QCM	NH_3_	RT/35% RH	184.4 Hz ppm^−1^	0.0160	N/A	^[^ ^228^ ^]^
La‐Ce MOF	QCM	DMA	RT/35% RH	92.9 Hz ppm^−1^	0.00946	N/A	^[^ ^228^ ^]^
La‐Ce MOF	QCM	TMA	RT/35% RH	59.4 Hz ppm^−1^	0.0158	N/A	^[^ ^228^ ^]^
La‐Ce MOF	QCM	Formaldehyde	RT/35% RH	102.6 Hz ppm^−1^	0.0427	N/A	^[^ ^228^ ^]^
ZIF‐67	Chemiresistive	Formaldehyde	150 °C/≈34% RH	13.9 (100 ppm)	N/A	N/A	^[^ ^264^ ^]^
ZIF‐67	Chemiresistive	TMA	75 °C/84% RH	10.3 (100 ppm)	2.0	>1000/>2000 (100 ppm)	^[^ ^265^ ^]^
NiPc‐Ni MOF	Chemiresistive	NH_3_	RT	43‐45% (80 ppm)	0.31	1800/– (80 ppm)	^[^ ^266^ ^]^
NiPc‐Cu MOF	Chemiresistive	H_2_S	RT	98% (80 ppm)	0.019	78/– (80 ppm)	^[^ ^266^ ^]^
NH_2_‐UiO‐66	Chemiresistive	SO_2_	150 °C	21.6% (10 ppm)	1.0	26.8/46.1 (10 ppm)	^[^ ^267^ ^]^
Cu_3_(HITP)_2_	Chemiresistive	NH_3_	RT	≈2.6% (10 ppm)	≈0.5	N/A	^[^ ^268^ ^]^
Cu_3_(HHTP)_2_	Chemiresistive	Methanol	RT	≈9.2% (200 ppm)	N/A	N/A	^[^ ^269^ ^]^
Cu_3_(HHTP)_2_	Chemiresistive	NO	RT	1.8% (80 ppm)	≈2.5	N/A	^[^ ^270^ ^]^
Cu‐HHB	Chemiresistive	H_2_S	RT	978% (100 ppm)	0.083	58.2/– (100 ppm)	^[^ ^271^ ^]^
VNU‐15	Chemiresistive	Acetone	50 °C	1.68 (10 ppm)	N/A	64/166 (10 ppm)	^[^ ^272^ ^]^
Zn‐MOF/GO	Chemiresistive	NH_3_	RT	13.2% (100 ppm)	N/A	102/127 (100 ppm)	^[^ ^273^ ^]^
Ni‐VNU‐74	Chemiresistive	CO	200 °C	1.7 (50 ppm)	N/A	N/A	^[^ ^274^ ^]^
CoPc‐O_8_‐Cu	Chemiresistive	CO	RT	27.4% (80 ppm)	0.53	N/A	^[^ ^91^ ^]^
NiPc‐O_8_‐Cu	Chemiresistive	CO	RT	18.9% (80 ppm)	3.0	N/A	^[^ ^91^ ^]^
ZnCo(NA)	Chemiresistive	Formaldehyde	RT	28.3 (50 ppm)	1.0	17/40 (50 ppm)	^[^ ^275^ ^]^
Ag@UiO‐66	Capacitive	H_2_S	RT	≈90% (100 ppm)	1.0	200/– (100 ppm)	^[^ ^276^ ^]^
RE‐fcu‐MOF	Capacitive	H_2_S	RT	≈14 × 10^−4^ (10 ppm)	0.005	N/A	^[^ ^277^ ^]^
Cu‐BTC (HKUST‐1)	Capacitive	Methanol	RT	≈25% (500 ppm)	47.3	N/A	^[^ ^278^ ^]^
Cu‐BTC (HKUST‐1)	Capacitive	Ethanol	RT	≈8% (500 ppm)	150.5	N/A	^[^ ^278^ ^]^
UiO‐66‐NH_2_	FET	DMMP	RT/50% RH	0.39 mV ppb^−1^	0.0002	N/A	^[^ ^279^ ^]^
Ni‐MOF‐74	Microcantilever	CO	RT	8.7 Hz (40 ppb)	0.01	N/A	^[^ ^280^ ^]^
HKUST‐1	Microcantilever	p‐Xylene	RT	≈60 Hz (50 ppm)	0.4	N/A	^[^ ^281^ ^]^
MOF‐5	Microcantilever	Aniline	RT/60% RH	≈2 Hz (10.8 ppm)	<1.4	108/– (10.8 ppm)	^[^ ^282^ ^]^
ZIF‐8	Optical (long‐period grating)	Ethanol	RT	0.0013 nm ppm^−1^	≈9.8	N/A	^[^ ^283^ ^]^
ZIF‐8	Optical (Fabry‒Perot)	Methanol	RT	0.000177 nm ppm^−1^	N/A	N/A	^[^ ^284^ ^]^
ZIF‐8	Optical (Fabry‒Perot)	Ethanol	RT	0.000412 nm ppm^−1^	N/A	30/48 (768.1 ppm)	^[^ ^284^ ^]^
ZIF‐8	Optical (Fabry‒Perot)	Ethanol	RT	0.018 nm ppm^−1^	5.56	N/A	^[^ ^285^ ^]^
ZIF‐8	Optical (Fabry‒Perot)	Acetone	RT	0.015 nm ppm^−1^	6.67	N/A	^[^ ^285^ ^]^
HKUST‐1	Optical (Fabry‒Perot)	Ethanol	70 °C	0.428 nm ppm^−1^	3.903	459.6/589.8 (24.9 ppm)	^[^ ^286^ ^]^
Pt/MOF‐5	Optical (Fiber‐Bragg grating)	H_2_	RT	0.541 nm/vol.%	N/A	7/8 (2‐4%)	^[^ ^287^ ^]^
Cu‐MOF/PAN	Optical (MZI)	Dimethyl carbonate	28 °C	0.093 nm ppm^−1^	0.053	N/A	^[^ ^288^ ^]^
ZIF‐8	Optical (SPR)	Methanol	RT	N/A	2.5	N/A	^[^ ^289^ ^]^
ZIF‐93	Optical (SPR)	n‐Butanol	RT	N/A	73	N/A	^[^ ^289^ ^]^
ZIF‐8	Optical (SERS)	Benzene	RT	N/A	540	N/A	^[^ ^290^ ^]^
MIL‐100(Fe)	Optical (SERS)	Toluene	RT	N/A	2.5	N/A	^[^ ^291^ ^]^
Au@ZIF‐8	Optical (SERS)	Benzaldehyde	RT	N/A	0.00032	N/A	^[^ ^292^ ^]^

*VNU, Vietnam National University; NA, Nicotinic acid; NiPc, Nickel phthalocyanine; NiNPc, Nickel naphthalocyanine; RE, Rare earth; DMMP, Dimethyl methylphosphonate; GO, Graphene oxide; PAN, polyacrylonitrile; MZI, Mach‒Zehnder interferometer.

## 5 Conclusions and Future Perspective

In the past decade, research on bimetallic MOFs has grown tremendously. Compared with monometallic MOFs, bimetallic MOFs offer several advantages because of the synergistic effects between the two metal centers, which can lead to enhanced structural stability, increased surface area, and improved functional performance. In the synthesis of bimetallic MOFs, the selection of two metals with similar characteristics, such as Coulombic charge, coordination number, ionic radius, and overall behavior, is crucial to increase the likelihood of their simultaneous and uniform incorporation into the framework. Although numerous studies have reported the fabrication of bimetallic MOFs with varying compositions and morphologies, limited efforts have been made to determine the precise metal arrangements within their structures (e.g., whether the metal ions are mixed within the same SBUs or segregated into distinct SBUs). Identifying these arrangements is critical for establishing clear correlations between the metal distribution, material properties, and sensing performance. Furthermore, additional experimental investigations into the thermal and chemical stability of bimetallic MOFs are needed to improve their feasibility for real‐world sensing applications.

From our literature survey, many bimetallic MOFs have been synthesized via hydrothermal or solvothermal methods, which require high temperatures and pressures and therefore involve high energy input. To increase the commercial feasibility of bimetallic MOFs for practical sensing applications, the development of scalable and energy‐efficient methods for their synthesis at ambient temperature and pressure is highly desirable. Moreover, the synthesis and optimization of bimetallic MOFs often require extensive trial‐and‐error experiments, which can be costly, time‐consuming, and inefficient. The integration of robotics with machine learning (ML) presents a promising solution by enabling automated synthesis and high‐throughput screening of bimetallic MOFs. ML algorithms can accelerate the optimization process by learning from prior experimental data and predicting optimal synthesis conditions (e.g., metal ratios, reaction temperatures, and reaction times). In addition, regression models and other ML tools can be employed to predict the properties of bimetallic MOFs on the basis of their composition and structure, allowing researchers to prioritize the most promising candidates. ML can also reveal the relationships among the structure, composition, and sensing performance of bimetallic MOFs toward various organic vapors. By leveraging these capabilities, ML can significantly reduce the time and cost associated with conventional synthesis methods, thereby accelerating the discovery and development of high‐performance, stable bimetallic MOFs for real‐world sensing applications.

Monitoring hazardous gases, including toxic gases and VOCs, is critical for protecting human health and the environment. QCM gas sensors offer the advantages of low‐temperature operation, low cost, simple fabrication, and ease of miniaturization. The recent emergence of MOFs as sensing materials for QCM gas sensors is driven by their exceptional structural and chemical properties. First, MOFs possess high surface areas and inherent porosity, providing abundant adsorption sites for gas molecules and facilitating efficient gas diffusion. In turn, these features enhance sensor sensitivity and enable rapid response and recovery. Second, their tunable pore sizes enable a molecular sieving effect, thereby improving selectivity toward specific target gases. Ideally, the pore size should be slightly larger than the target gas molecule to achieve both sensitive and selective detection. Additionally, the presence of diverse functional groups in MOFs allows for specific chemical interactions with gas molecules (e.g., hydrogen bonding, van der Waals forces, and π–π interactions), which further increase their sensing performance. In addition to pore size, other structural features of MOFs, such as particle size and morphology, can be tuned by modifying synthesis conditions (e.g., reaction temperature, reaction time, and metal‐to‐ligand ratio) to optimize sensitivity and selectivity. Generally, MOFs with smaller particle sizes are preferred, as they reduce the diffusion distance for gas molecules during sensing. Moreover, low‐dimensional MOFs, particularly 2D structures such as nanosheets, offer a greater surface area and porosity, which enhances gas adsorption and accelerates diffusion, leading to enhanced sensitivity and response‐recovery properties. Notably, the thickness of 2D MOF nanosheets can be precisely controlled to optimize the detection of specific analytes. This effect was demonstrated in a recent study on Cu‐TCPP‐Cu MOF nanosheets, where variations in nanosheet thickness resulted in different responses toward benzene, toluene, ethylbenzene, and p‐xylene.

The incorporation of secondary metal nodes in bimetallic MOFs offers several advantages, including (i) the introduction of additional unsaturated metal sites that promote stronger gas‒framework interactions, (ii) enhanced charge transfer and chemical interactions compared with those of monometallic MOFs, and (iii) increased SSA and porosity when the metal ratio is optimally tuned. Owing to these benefits, bimetallic MOFs have demonstrated superior sensing performance over their monometallic counterparts in QCM sensors for detecting water vapor (humidity) and various organic vapors, including α‐pinene, NH_3_, and amine‐based compounds (TMA and DMA).^[^ ^10^, ^13^, ^228^, ^261^ ^]^ In terms of sensing mechanisms, humidity detection via MOF‐based QCM sensors (including those based on bimetallic MOFs) is driven primarily by hydrogen bonding between water molecules and the functional groups of organic ligands within the porous framework.^[^ ^233^, ^240^ ^]^ In contrast, the detection of aromatic hydrocarbons (e.g., benzene and toluene) predominantly relies on π–π interactions with the aromatic components of the MOF structure.^[^ ^246^ ^]^

Our literature survey revealed that the majority of studies have focused on QCM gas sensors based on pure MOFs, with significantly fewer reports exploring bimetallic MOF‐based systems. Given the superior properties and sensing performance of bimetallic MOFs, future research should prioritize the development of high‐performance QCM sensors using these materials. Furthermore, although some theoretical and experimental studies have explored the role of different metal centers in bimetallic MOFs for gas detection, research in this area remains limited. Therefore, additional theoretical modeling and in situ experimental investigations are needed to gain deeper insights into the underlying sensing mechanisms. This understanding will be essential for the rational design of bimetallic MOFs with tunable selectivities toward specific target gases, thereby helping minimize cross‐sensitivity issues.

The direct utilization of bimetallic MOFs (without postsynthetic modifications or heat treatments) in QCM gas sensors can simplify fabrication and reduce costs for practical gas monitoring applications. To further enhance their real‐world applicability, a portable e‐nose incorporating a bimetallic MOF microarray paired with ML algorithms can be developed to improve sensitivity and enable more accurate discrimination of target gases from interfering species. A notable example is the recently developed UiO‐66‐based QCM microarray sensor, which has been successfully used for fruit ripeness indexing.^[^ ^293^ ^]^ Furthermore, bimetallic MOFs can be integrated with other sensing materials, such as semiconducting metal oxides or carbon nanotubes (CNTs), to further increase their sensitivity, selectivity, and long‐term stability for hazardous gas detection in real‐world environments.

## Conflict of Interest

The authors declare no conflict of interest.
